# Microbial fingerprinting of marine water masses in an Antarctic and hydrographically complex area

**DOI:** 10.1186/s12915-026-02621-8

**Published:** 2026-05-23

**Authors:** Mireia Mestre, Alicia Prior, Camila Marín-Arias, Daniel R. Rodríguez-Solís, Rafael Laso-Pérez, Emilio Alarcón, Valeska Vásquez-Lepio, Humberto E. González, Ramiro Logares, Jesse McNichol, Jed Fuhrman, Camila Fernandez, Mark J. Hopwood, Juan Höfer

**Affiliations:** 1 National Museum of Natural Sciences (MNCN-CSIC), Madrid, Spain; 2 COPAS-Coastal Center, Universidad de Concepción (UdeC), Concepción, Chile; 3 Centro de Investigación en Dinámica de Ecosistemas Marinos de Altas Latitudes (IDEAL), Valdivia, Chile; 4 Programa de Magister en Oceanografía, Escuela de Ciencias del Mar, Pontificia Universidad Católica de Valparaíso, Valparaíso, Chile; 5 GEMA, Center for Genomics, Ecology & Environment, Universidad Mayor, Santiago, Chile; 6 Institute of Marine Sciences (ICM-CSIC), Barcelona, Spain; 7 Department of Biological Sciences, University of Southern California, Los Angeles, USA; 8 Department of Biology, St. Francis Xavier University, Antigonish, Canada; 9 Laboratory of Microbial Oceanography, Banyuls Sur Mer, CNRS UMR7621 France; 10 Southern University of Science and Technology (SUSTech), Shenzhen, China; 11 Escuela de Ciencias del Mar, Pontificia Universidad Católica de Valparaíso, Valparaíso, Chile

**Keywords:** Microbial fingerprinting, Water masses, Southern Ocean, Antarctica, Marine ecosystems, Microbial communities, Biogeography

## Abstract

**Background:**

Microorganisms are ubiquitous in marine ecosystems, yet the influence of water masses on their distribution remains underexplored, particularly in extreme environments such as Antarctica. This study examines microbial communities in the Gerlache-Bismarck Strait, a region with complex hydrography. We analyzed both prokaryotic and eukaryotic diversity alongside multiple oceanographic and biogeochemical variables. Water samples were collected from 1 to 400 m depth across three size fractions: pico- (0.2–3 µm), nano- (3–20 µm), and microparticles (20–200 µm).

**Results:**

Our results indicate that water mass composition is a primary driver of microbial community assembly. Surface water masses included Antarctic Surface Waters (AASW), Glacially Modified Waters (GMW), and Transitional Zonal Waters influenced by the Bellingshausen Sea (TBW), whereas intermediate and deep layers comprised Transitional Zonal Waters influenced by the Weddell Sea (TWW) and Circumpolar Deep Waters (CDW). This study provides direct evidence of close links between microorganisms and water masses, showing that marine microbial communities are shaped not only by local conditions but also by water masses circulation. We use the concept of “microbial fingerprinting of water masses” establishing microbial communities as ecological indicators and providing framework applicable to other marine systems.

**Conclusions:**

Microbial fingerprints provided insight into the role of the water masses in biogeochemistry and food webs of this area (e.g., the ammonia-oxidizing archaea Nitrosopumilaceae in the CDW, and the phototroph *Chrysochromulina simplex* in the GMW). This information is useful to predict how future changes in ocean circulation and microbial distribution may alter ecosystem services in this critical region.

**Supplementary Information:**

The online version contains supplementary material available at 10.1186/s12915-026-02621-8.

## Background

Marine microorganisms are key components of marine food webs, biogeochemical cycles, and carbon cycling processes. Substantial progress has been made in recent decades in characterizing microbial communities in the Southern Ocean, but some aspects of their diversity, spatial patterns, and functional roles remain to be studied in more detail [1–4].

Coastal areas of the Southern Ocean are highly productive [5], in contrast to their offshore counterparts, which are characterized as high-nutrient-low-chlorophyll regions (HNLC). In particular, the coastal waters of the Western Antarctic Peninsula fuel massive phytoplankton blooms and sustain high levels of primary productivity [6, 7], supporting large stocks of krill, marine mammals, and birds [8]. Ecosystems in this area are undergoing significant changes due to human impacts and climate change: Antarctic human activities are concentrated in this area, which experiences the highest levels of ship traffic, fishery exploitation, tourism, and scientific activity in Antarctica [9], and the surrounding maritime area is recognized as one of the regions most affected by climate change [10]. Climate change is altering the physical drivers of productivity in the Southern Ocean [11–13], which subsequently affects marine biota, from microorganisms to whales [14]. In the Western Antarctic Peninsula, the marine area is experiencing a rapid increase in the average annual temperature, increasing stratification of the water column during warmer months, loss of sea ice, retreat of glaciers and ice shelves, and increased runoff. These shifts are driving significant ecosystem changes [15–18], with observed impacts on phytoplankton and microbial dynamics due to warming surface waters and enhanced glacial meltwater input [19–22].

A marine protected area has been proposed for the Western Antarctic Peninsula [23], where the Gerlache Strait, in particular, has been presented as the zone that most requires the implementation of conservation actions [24, 25]. The Gerlache Strait is a 200 km long strait that is connected in the north to the Bransfield Strait, and to the south with the Bismarck Strait. The Gerlache-Bismarck Strait is a particularly productive area [26–28] where krill and whales aggregate [29–32]. The hydrography of the Gerlache-Bismarck Strait is complex because of the multiple geographic components (islands, channels, glaciers, and straits) and the confluence of different currents and water masses [33–36]. These physical dynamics, in turn, determine the distribution of nutrients [37], phytoplankton [18, 27, 38], and zooplankton [33]. However, information concerning the diversity and distribution of microorganisms inhabiting the Gerlache-Bismarck Strait is still scarce.

Microorganisms include two evolutionarily distinct groups: unicellular eukaryotes (protists) and prokaryotes (bacteria and archaea). In Antarctica, pelagic microbial communities include eukaryotes (phytoplankton and other groups such as dinoflagellates, ciliates, and flagellates, see e.g., [3, 39]), bacteria (dominated by Gammaproteobacteria, Alphaproteobacteria, and Bacteroidota) and archaea (which includes Thermoplasmatota, formerly within the Euryarchaeota; and Crenarchaeota, also known as Thermoproteota) [1, 4, 40, 41]. In the Gerlache Strait, different phytoplankton communities display contrasting distributions, where cryptophytes and *Phaeocystis* sp. tend to be the most abundant groups, while diatoms, though being less abundant, dominate primary production [27, 42–44]. Crenarchaeota dominates among other archaea [45, 46] while information on bacterial diversity remains scarce. In summary, microbial studies in the Gerlache Strait have primarily focused on phytoplankton community composition and have predominantly relied on traditional microscopy-based taxonomic analyses. Technologies such as high-throughput DNA sequencing have the potential to unveil hidden microbial diversity that is normally underestimated by more traditional methodologies [3], and studies in the Gerlache-Bismarck Strait implementing high-throughput DNA sequencing are still lacking.

In this study, we investigated the diversity of microbial communities (both prokaryotic and eukaryotic, from 0.2 to 200 µm in size) present along the Gerlache-Bismarck Strait during summer. We included contrasting areas, such as those influenced by freshwater inputs coming from melting glaciers and those that feature an inflow of seawater from the open ocean. Aiming to bring together oceanography and microbiology, we integrated microbial distributions with an analysis of environmental variables and the different water masses present in the Gerlache-Bismarck Strait. Our results indicate a tight connection between microbial communities and water masses, where each water mass harbored specific communities. Building on previous evidence of water-mass–specific microorganisms, we introduce the concept of “microbial fingerprint of water masses” (i.e., a specific microbial assemblage for a water mass), and we propose a methodology to explore it. These microbial fingerprints provided information about the role of the different water masses in regional biogeochemical and ecological processes, highlighting microorganisms-water mass linkages as key dynamic features of the ecosystem functioning in the Gerlache-Bismarck Strait. This information will help to predict how future changes in ocean circulation and microbial diversity and distribution may alter ecosystem services in this critical region.

A Spanish translation of the abstract is provided in Additional file 1.

## Results

### Oceanographic context

**Fig. 1:**
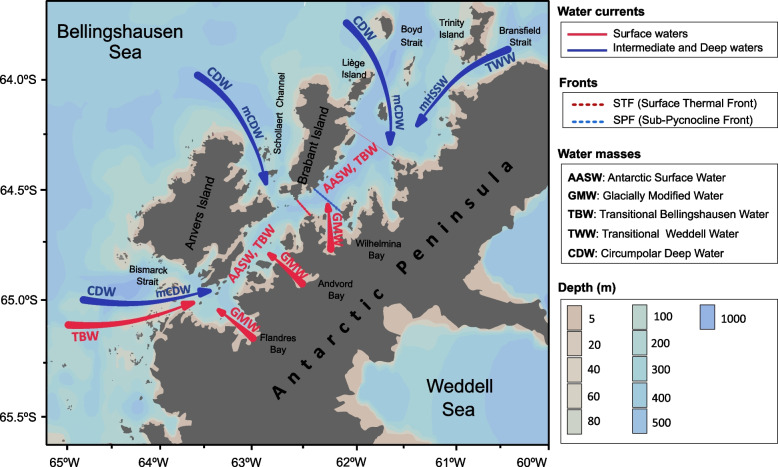
Gerlache-Bismarck Strait oceanography. Map of the Gerlache-Bismarck Strait indicating the major currents, water masses, and fronts in the area. The arrows indicate the direction of the major currents, which are usually linked to the water masses flow. Dashed lines indicate where the fronts are normally found. Information about major currents, water masses and fronts were obtained from: [18, 33–35, 37, 53, 54]. The arrow of the TBW was defined based on literature in a nearby area [54]

The Gerlache-Bismarck Strait is surrounded at the east by the Antarctic Peninsula and at the west by Anvers and Brabant Islands (Fig. 1). Oceanographic studies in this area have been conducted primarily during the spring–summer, when it is sea ice–free. Similarly, our sampling was conducted in February 2020, after the sea ice in this region had retreated by September–October 2019 [47]. Seawater in the Gerlache-Bismarck Strait (Fig. 1) originates mainly from the Bellingshausen Sea (entering mainly through the Gerlache and Bismarck straits, and Schollaert Channel) and the Weddell Sea (entering through the Bransfield Strait). The dominant current is the Gerlache Strait Current, which flows NE transporting most of the Gerlache Strait waters (including surface and deep waters) towards the Bransfield Strait [33, 36, 48]. The opposite current is the Bransfield Strait Current, which moves waters from the Bransfield Strait towards the Gerlache Strait [18, 34]. Water currents in the Gerlache Strait are linked to the ACC/APCC (Antarctic Circumpolar Current/Antarctic Peninsula Coastal Current) transport system [18, 34].

During summer, there is a permanent pycnocline at approximately 100 m which separates surface and intermediate water layers [35]. Two main fronts have been described in the Gerlache Strait: one above the pycnocline, the surface water thermal front (STF), and another below the pycnocline, the sub-pycnocline front (SPF). The STF is located close to the Schollaert Channel, and is permanent during the austral summer [35]. The SPF has a variable location along the strait, but is typically found at depths between 100 and 400 m depth [35]. In this region of Antarctica, the water column is commonly divided into surface (0 m—100 m), intermediate (100 m—300 m), and deep waters (below 300 m to the seafloor) [35].

Surface waters in the Gerlache Strait (from 0 to 100 m) have variable characteristics and are affected by multiple processes throughout the year, such as sea ice formation and melting, water mixing, wind forcing, and solar heating. In summer, surface water is continuously mixed [35]. Surface waters include cold waters, such as Transitional Zonal Waters with Bellingshausen Sea influence (TBW) and Antarctic Surface Waters (AASW) [37]. Surface waters also evidence Glacially Modified Waters (GMW), which originates from melting glaciers and typically forms a thin (20—30 m) layer at the surface extending from inner fjords and bays into the strait [49–51].

Intermediate waters in the Gerlache-Bismarck Strait (from 100 to 300 m) are characterized by a mixing of different waters coming from the Bellingshausen and Weddell seas [18], which include Circumpolar Deep Waters (CDW), Transitional Zonal Waters with Weddell Sea Influence (TWW), and High Salinity Shelf Water (HSSW). Circumpolar Deep Water (CDW) is a relatively warm, salty, and low-oxygenated water mass [52, 53]. CDW enters the area through the Bismarck Strait (Palmer Deep Canyon), Schollaert Channel, and north of Liège Island. CDW dominates deeper waters in the Bismarck Strait, which is the most important CDW path into the Gerlache Strait [35]. CDW transforms into modified-CDW (mCDW) when it enters the Gerlache-Bismarck Strait [18, 37]. CDW can be divided into Upper (UCDW) and Lower (LCDW, normally between 800 and 1000 m) CDW [53], and CDW flowing from the Bellingshausen Sea into the Gerlache-Bismarck Strait has been described either as LCDW [37] or UCDW [52]. TWW water mass is generated in the Weddell Sea and goes through the Bransfield Strait right next to the Antarctic Peninsula towards the northern end of the Gerlache Strait. TWW is found between 300 and 700 m depth [37, 54]. The intermediate and deep-water masses in the Gerlache Strait from the Weddell Sea have been reported as a modified version of the HSSW (mHSSW in [35]; HSSW-derived in [18]). The mHSSW enters the Gerlache Strait as bottom water and is characterized by low temperature, high salinity, and high oxygen concentration [52]. This water mass extends to the southernmost point of the Western Antarctic Peninsula in the central area of the Gerlache Strait (around the Schollaert Channel), becoming scarce in the southern Gerlache Strait, and negligible in the Bismarck Strait due to bathymetric barriers. Generally, in the northern part of the Gerlache Strait, mCDW intrudes the TWW [35, 37, 52], forming the sub-pycnocline front (SPF) [35], which separates the older and warmer waters coming from the Bellingshausen Sea entering from the Bismarck Strait from the younger and colder ones coming from the Weddell Sea [18, 35, 52].

A summary of the main oceanographic features of the Gerlache-Bismarck Strait, together with their abbreviations and thermohaline characteristics are provided in Additional file 2: Tables S1 and S2.

### The environmental context

**Fig. 2:**
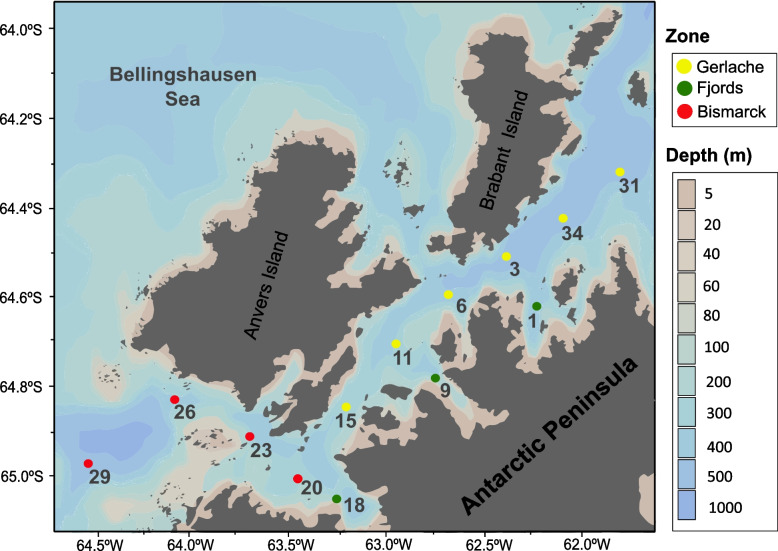
Stations sampled. Samples were collected from three different zones: the Gerlache Strait zone (yellow), that is, the central part of the Gerlache Strait; the Fjords zone (green), that is, the stations influenced by local meltwater outflow coming from glaciers, including three bays, Wilhelmina Bay, Andvord Bay, and Flandres Bay (from north to south); and the Bismarck Strait zone (red), including the stations that are closer to the open sea (Bellinghausen Sea)

Sampling included 13 oceanographic stations along the Gerlache-Bismarck Strait (Fig. 2, Additional file 2: Table S3), including depths from 1 to 400 m and 3 fjords: Wilhelmina Bay, Andvord Bay, and Flandres Bay. Environmental variables exhibited high heterogeneity across stations and depths (Additional file 2: Figures S1-S6). Nevertheless, some general patterns emerged: variability in surface waters mirrored that of the deep layers, and certain parameters increased from north to south (e.g., chlorophyll), while others decreased (e.g., salinity). In particular, for surface waters (i.e., 1 m depth), the levels of oxygen increased towards the south (from 8.17 to 8.96 mg mL^−1^) and the same trend was evident with chlorophyll (from 1.10 to 2.06 mg m^−3^), whereas salinity decreased (from 33.14 to 32.94 practical salinity). In the middle parts of the Gerlache-Bismarck Strait (stations 11 and 15), we found lower temperature (1.18 ºC) and chlorophyll (~ 1.4 mg m^−3^) and higher salinity (33.79 practical salinity), dissolved silicate (66.27 µM), dissolved nitrate (21–22 µM) and dissolved phosphate (1.9 µM). When comparing the inshore fjords, chlorophyll concentrations were lowest in Wilhelmina Bay (0.09 mg m⁻^3^) and higher in Andvord Bay (0.51 mg m⁻^3^) and Flandres Bay (1.00 mg m⁻^3^). Similarly, dissolved nutrient concentrations were lower in Wilhelmina Bay (nitrate: 16.39 μM; phosphate: 1.33 μM) than in Andvord Bay (nitrate: 20.27 μM; phosphate: 1.61 μM) and Flandres Bay (nitrate: 19.34 μM; phosphate: 1.63 μM). In terms of vertical variability, from the surface to the deeper waters, we observed a decreasing pattern in the mean values of oxygen (from 8.22 to 5.78 mg mL^−1^), temperature (from 1.57 to 0.18 ºC), chlorophyll (from 1.33 to 0.03 mg m^−3^) and in situ pH (from 8.04 to 7.93), whereas increases were observed for salinity (from 33.41 to 34.49 practical salinity), dissolved nitrate (from 20.35 to 27.25 µM), dissolved phosphate (from 1.77 to 2.27 µM) and dissolved silicate (from 57.68 to 71.98 µM).

**Fig. 3:**
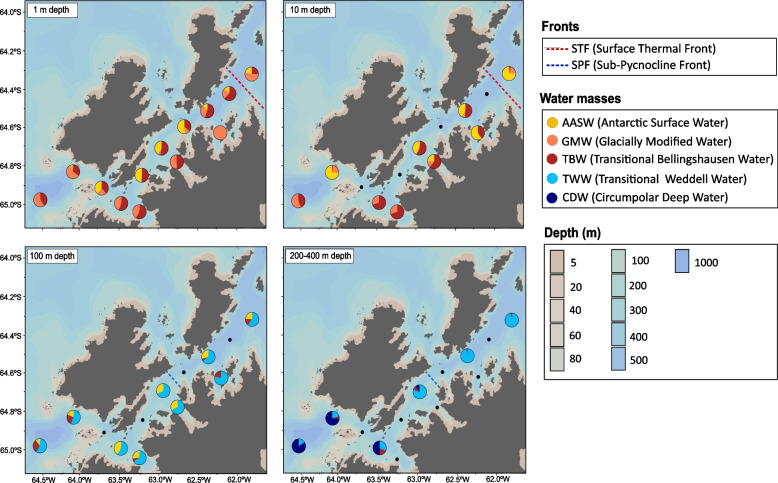
Water masses present in the Gerlache-Bismarck Strait, at each station sampled and depth. Each pie chart represents a station and colors represent different water masses. The dashed lines represent the areas where fronts were detected: the red line represents the Surface Thermal Front (STF) and the blue one the Sub-Pycnocline Front (SPF)

Based on the physical properties of the seawater, we detected the summer pycnocline at a depth of approximately 100 m, the STF in the northern part of the Gerlache-Bismarck Strait (around stations 31 and 34), and the SPF in the middle part of the strait (between stations 6 and 11) (Additional file 2: Figure S1). Five water masses were present: AASW, GMW, TBW, TWW and CDW (Fig. 3). Surface waters were dominated by AASW, GMW and TBW, where GMW was more evident within fjords, particularly in Wilhelmina Bay. In the intermediate and deep waters, the most relevant water masses included TWW and CDW, where TWW dominated in the northern part and CDW in the south (Fig. 3, Additional file 2: Table S1).

### Microbial diversity and composition

We observed high heterogeneity and contrasting values of richness among stations, depths, and size fractions (Additional file 2: Figures S7, S8, S9). The highest microbial diversity values appeared in surface waters close to Bismarck Strait (station 23), whereas the lowest values were found at the northernmost stations (stations 31 and 34). There was an increase in diversity close to the Schollaert Channel (stations 3, 6, and 11), which decreased towards the southern stations. Regarding the size fractions (Additional file 2: Figure S9), we observed that, in general, diversity increased with size fraction at 1 m, decreased with size fraction at 10 and 100 m, while in deeper waters, the intermediate size fraction 3—20 μm had the lowest diversity.

In total, we obtained 13,148 ASVs from prokaryotes, comprising 13,055 bacterial and 93 archaeal ASVs, as well as 399 ASVs from eukaryotes, including 182 from fungi. In terms of number of sequences, bacteria dominated community composition at all stations, fractions, and depths (98.32% of sequences), followed by eukaryotes (1.47%) and archaea (0.21%). Fungi (included within eukaryotes) corresponded to 0.95% of total sequences. The most dominant phylum of bacteria was Bacteroidota, followed by Proteobacteria (composed mainly by Alpha- and Gamma- proteobacteria), and Planctomycetota. The most abundant phylum of eukaryotes was Fungi, followed by Cryptophyta, Pseudofungi and Haptophyta, and the most abundant phylum of archaea was Crenarchaeota, followed by Nanoarchaeota and Thermoplasmatota. The contribution of each taxa varied among stations, water masses, depths, and size fractions (Additional file 2: Figures S10-S15): Certain groups were highly abundant in surface waters, such as Bacteroidota, and some became more dominant with depth, such as Acidobacteria and Planctomycetota. We also observed differences among size fractions; some microorganisms appeared more frequently in the smallest size fractions, such as specific groups of Alphaproteobacteria, whereas others were more common in the medium and large size fractions, such as Bacillariophyta (Ochrophyta), Dinoflagellata, and Apicomplexa. Other microorganisms, such as Basidiomycota (phylum Fungi), appeared at a similar frequency across all three size fractions.

We assessed the variability of microbial communities among stations, depth, water masses and size fractions, by testing each main factor independently and, according to the PERMANOVA analysis, most factors were statistically significant, where the proportion of the total variation explained was relatively low (station, R^2^ = 0.11939, *p*-value = 0.213; depth, R^2^ = 0.10028, *p*-value = 0.001; water mass, R^2^ = 0.10437, *p*-value = 0.001; size fraction, R^2^ = 0.04026, *p*-value = 0.001). We also observed an overlap between the effects of depth and water masses on microbial communities when tested together (PERMANOVA with marginal effects: depth R^2^ = 0.0227, *p* = 0.075; water masses R^2^ = 0.0268, *p* = 0.361), indicating that, in this area, both factors are closely correlated (in the Gerlache Strait, water masses are situated at specific depths). As water masses accounted for a larger proportion of the explained variability, and because water masses integrate multiple hydrographic properties including depth, water mass composition was retained as an explanatory variable for subsequent analyses.

### Influence of the environment on microbial communities

**Fig. 4:**
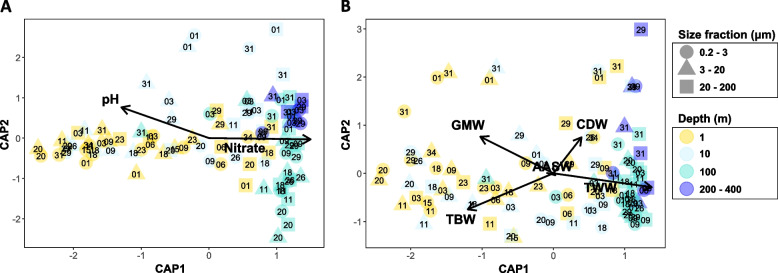
Microbial community variability explained by (**A**) environmental variables and (**B**) water masses. The dbRDA ordination exhibits the links between axes and the variables constraining microbial communities. Samples are numbered by station, shaped by size fraction, and colored by depth. Abbreviations in (**A**) include pH and Nitrate. Abbreviations in (**B**) represent: AASW (Antarctic Surface Waters), GMW (Glacially Modified Waters), TBW (Transitional Zonal Waters with Bellingshausen Sea influence), TWW (Transitional Zonal Waters with Weddell Sea Influence), CDW (Circumpolar Deep Waters). Details about the dbRDA ordination and the selection of variables can be found in the Materials and Methods and Results sections

The different zones (Gerlache Strait, fjords, and Bismarck Strait) were not relevant for explaining the variability in the microbial communities (PERMANOVA: R^2^ = 0.02196, *p*-value = 0.171). However, microbial communities were different when comparing each side of the fronts (Surface Thermal Front, PERMANOVA: R^2^ = 0.03367, *p*-value = 0.011; Sub-Pycnocline Front, PERMANOVA: R^2^ = 0.03047, *p*-value = 0.005). The subset of environmental variables that better explained the variability in community composition (selected with the *bioenv* function from *vegan* package) were nitrate and pH (Fig. 4A, Additional file 2: Figure S16). Different combinations of these variables were relevant for each size fraction (Additional file 2: Figure S17). The water masses that better explained the variability in community composition included AASW, GMW, TBW, TWW, and CDW (none were discarded by the *bioenv* function) (Fig. 4B), even when the different size fractions were considered separately (Additional file 2: Figure S18). Variability in microbial communities (dbRDA, *capscale* function) was better explained by the mixing of water masses (adjusted R^2^ = 0.051) than by the environmental variables (adjusted R^2^ = 0.042) (Fig. 4), revealing that water mass legacy had a great influence on microbial communities. A permutational ANOVA test confirmed the significant effect of the water masses on the structure of microbial communities (*p*-value < 0.05).

Altogether, some generalities can be described regarding the influence of the environment on microbial communities: above 100 m, microorganisms were linked to surface water masses (such as AASW, GMW, and TBW), and were affected by pH, a proxy of local productivity. In contrast, below 100 m, microbial communities were linked to intermediate and deep-water masses (TWW and CDW) and were influenced by higher nitrate concentrations, a proxy of CDW intrusions (Fig. 4).

### Microbial fingerprinting of the water masses

**Fig. 5:**
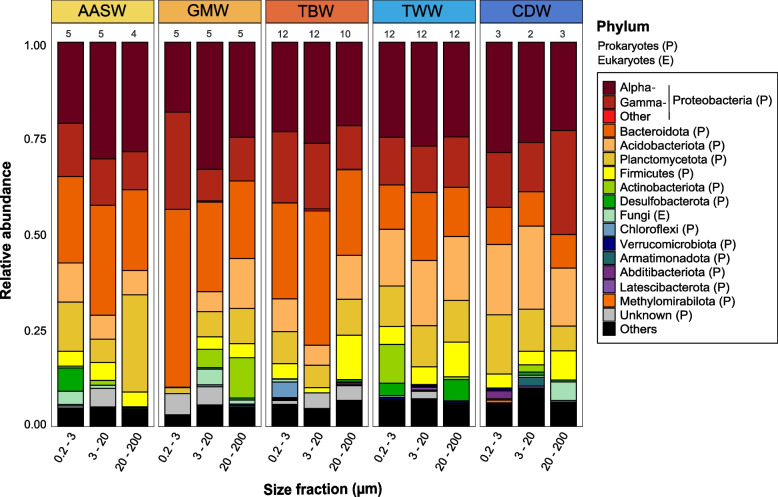
Community composition of each water mass. Bar plot showing the 15 most abundant taxa at the phylum level across each water mass and size fraction. The category “Others”, includes low abundant taxa and low abundant ASVs (< 1% total abundance). Numbers above bars indicate the number of samples included in each bar. Abbreviations of water masses: AASW (Antarctic Surface Waters), GMW (Glacially Modified Waters), TBW (Transitional Zonal Waters with Bellingshausen Sea influence), TWW (Transitional Zonal Waters with Weddell Sea Influence), CDW (Circumpolar Deep Waters)

**Fig. 6:**
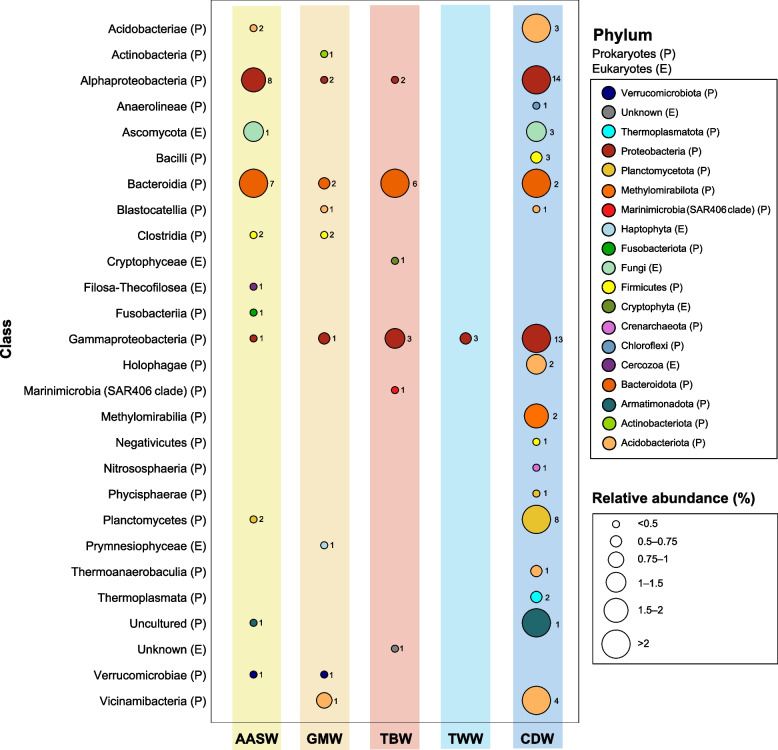
Indicator microorganisms of each water mass. Indicator microorganisms are calculated at the ASVs level, represented at the Class taxonomic levels and colored by Phylum. The size of the circles is proportional to the relative abundances of indicator ASVs in each water mass. Numbers next to the circles indicate the number of indicators ASVs included. Abbreviations of water masses include: AASW (Antarctic Surface Waters), GMW (Glacially Modified Waters), TBW (Transitional Zonal Waters with Bellingshausen Sea influence), TWW (Transitional Zonal Waters with Weddell Sea Influence), CDW (Circumpolar Deep Waters)

**Fig. 7:**
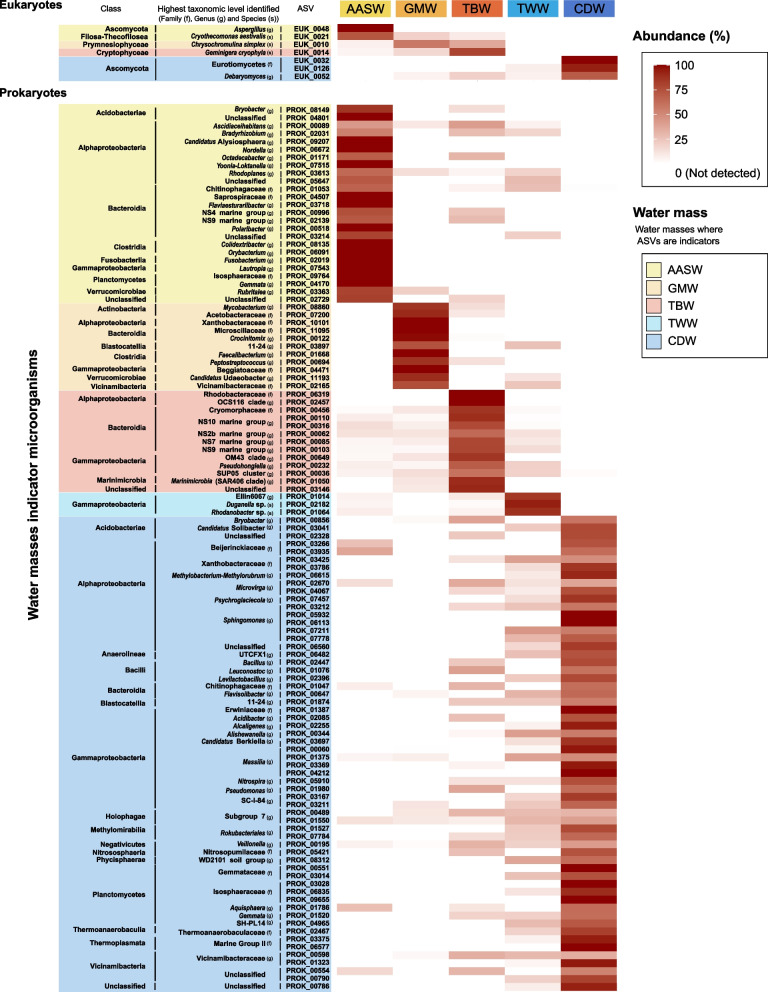
Distribution of indicator microorganisms across water masses. The heatmap shows the indicator taxa of each water mass and their relative presence across all water masses. For each indicator microorganism, the taxonomic class, the highest resolved taxonomic rank (family, genus, or species), and the corresponding ASV identifier are shown. Relative abundances of each indicator microbe were scaled so that their individual contributions sum to 100%. Color intensity represents the proportional abundance of each ASV within a given water mass, with darker (more saturated) shades indicating higher relative abundance and white indicating absence (non-detection). Water mass abbreviations: AASW (Antarctic Surface Waters), GMW (Glacially Modified Waters), TBW (Transitional Zonal Waters influenced by the Bellingshausen Sea), TWW (Transitional Zonal Waters influenced by the Weddell Sea), and CDW (Circumpolar Deep Waters)

Although each water mass harbored specific microbial communities, the phyla Bacteroidota, Proteobacteria (mainly classes Alpha- and Gamma- proteobacteria), and Planctomycetota were dominant in all water masses (Fig. 5, Additional file 2: Figure S19, S20, S21, S22). Each water mass contained a specific list of indicator microorganisms (Figs. 6 and 7, Additional file 2: Tables S4, S6). Indicator microorganisms were diverse, but each water mass harbored specific taxa. The water mass with the highest number of indicator microorganisms was CDW (*N* = 63), followed by AASW (*N* = 27), suggesting that these water masses harbor multiple and very specific niches that are not present in the other water masses.

**Fig. 8:**
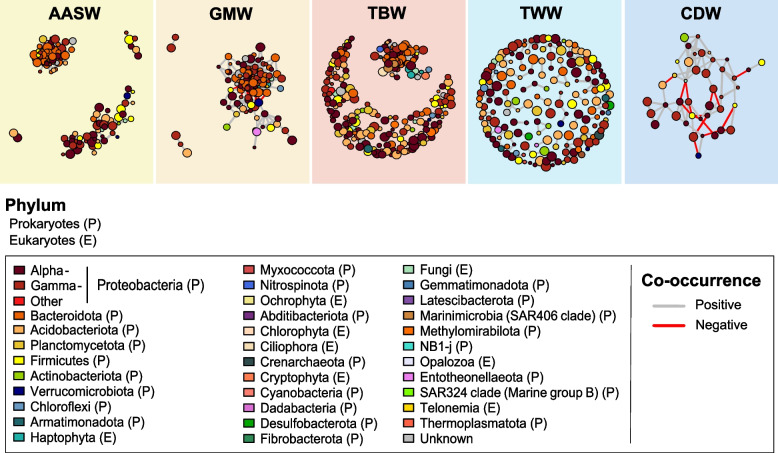
Co-occurrence and co-exclusion networks of each water mass. Nodes correspond to ASVs, which are colored by phylum (and Proteobacteria by class). Gray lines indicate positive interactions between different nodes, whereas red lines represent negative interactions. The size of the node represents the relative abundance of each node; the larger the node, the more abundant it is. Network parameters can be found in Additional file 2: Table S8. Details about the network construction can be found in the Materials and Methods section. Abbreviations of water masses include: AASW (Antarctic Surface Waters), GMW (Glacially Modified Waters), TBW (Transitional Zonal Waters with Bellingshausen Sea influence), TWW (Transitional Zonal Waters with Weddell Sea Influence), CDW (Circumpolar Deep Waters)

Each water mass was composed of a different core microbiome (Additional file 2: Table S5, S7). The core microbiome of the different water masses was generally dominated by abundant groups, such as Bacteroidota and Proteobacteria, with the genus *Methylobacterium* being part of the core in all water masses. In surface water masses, the core microbiome was dominated by Proteobacteria, and the genera *Polaribacter* and *Sulfitobacter* (both Proteobacteria) were present in nearly all surface water masses (AASW, GMW, and TBW). The core microbiome of intermediate and deep waters was dominated by Proteobacteria, with the order Burkholderiales present in all deep-water masses (TWW, CDW). Microbial co-occurrence networks (Fig. 8) were composed principally of abundant taxa, such as Alpha- and Gamma- proteobacteria, Bacteroidota, and Acidobacteriota (Fig. 5). Each water mass harbored a distinctive and, in general, highly cohesive microbial network (Fig. 8, Additional file 2: Table S8), where potential interactions within each water mass were higher than those among water masses (modularity values for each individual water mass were lower than those calculated for all samples, except for TWW). Co-occurrence network parameters (Additional file 2: Table S8) revealed that AASW and GMW had higher values of average degree, clustering, and density, and lower values of modularity and average distances than CDW, TBW and TWW water masses. The water mass with a less cohesive community was TWW. Together, these results indicate that microbial communities in AASW and GMW were more complex and interconnected than those in CDW, TBW and TWW, and that AASW and GMW may harbor more coupled and stable communities than CDW, TBW and TWW. In particular, the microbial fingerprint of each water mass can be summarized as:

AASW was dominated by the phyla Proteobacteria (mainly Alpha-) and Bacteroidota, and Planctomycetota were more dominant in larger size-fractions. The most abundant eukaryotic phyla were Fungi and Apicomplexa. The core microbiome of AASW consisted of 3 ASVs, which belong to the Classes Alphaproteobacteria (Rhizobial *Methylobacterium* and Rhodobacteral *Sulfitobacter*) and Gammaproteobacteria (Pseudomonadales), which together occupied a relevant part (15.72% of sequences) of the community. The indicator microorganisms of AASW included 27 ASVs, two of which belonged to higher-level taxa (phyla) not founded in other water masses: the eukaryotic phylum Cercozoa (*Cryothecomonas aestivalis,* Order Cryomonadida) and the prokaryotic phylum Fusobacteriota (Genus *Fusobacterium*, Order Fusobacteriales). 

GMW was dominated by Bacteroidota, and Proteobacteria (mainly Alpha- and Gamma-), where Gammaproteobacteria were more dominant in the smaller size-fractions. The most abundant eukaryotic phyla were Haptophyta and Fungi. The core of the GMW consists of 6 ASVs, including multiple phyla (Bacteroidota, Firmicutes, Proteobacteria, Verrucomicrobiota, Actinobacteriota, and Acidobacteriota), accounting for a considerable percentage (26.03% of sequences) of the community. The indicator microorganisms of GMW included a total of 12 ASVs, two of which belong to higher-level taxa (phyla) not found in other water masses: the phylum Haptophyta (*Chrysochromulina simplex*, Order Prymnesiales) and the phylum Actinobacteriota (*Mycobacterium* sp., Order Corynebacteriales).

TBW was dominated by the phyla Bacteroidota and Proteobacteria (mainly Alpha-). The most abundant eukaryotic phyla were Cryptophyta and Fungi. The core of the TBW consisted of 11 ASVs, all of them Bacteroidota or Proteobacteria, which accounted for a substantial percentage (26.16% of sequences) of the community. The indicator microorganisms of TBW included 14 ASVs, one of which belonged to higher-level taxa (phyla) not found in other water masses: the candidate phylum Marinimicrobia (SAR406) and the phylum Cryptophyta (*Geminigera cryophila,* Order Cryptomonadales).

TWW water mass was dominated by the phyla Proteobacteria (mainly Alpha-), Bacteroidota, Planctomycetota, and Acidobacteriota, with the most abundant phyla of eukaryotes being Dinoflagellata and Fungi. The core microbiome consisted of 4 ASVs, all of which belong to the phylum Proteobacteria and cover 17.48% of the sequences. The indicator microorganisms of TWW water masses included a total of 3 ASVs, which all correspond to the phylum Proteobacteria and to the families Nitrosomonadaceae, Rhodanobacteraceae, and Oxalobacteraceae.

CDW water mass was dominated by the phyla Proteobacteria (mainly Alpha-) and Acidobacteria, with the most abundant eukaryotic phylum being Fungi. The core of the CDW consisted of 6 ASVs, which correspond to the phyla Proteobacteria and Firmicutes, accounting for an abundant part (20.46% of sequences) of the microbial community. The indicator microorganisms of CDW included 63 ASVs, all of which were prokaryotes, and included four phyla not found in the other water masses: one phylum of bacteria (Chloroflexi, Methylomirabilota), and two of archaea (Thermoplasmatota, Crenarchaeota).

## Discussion

Here, we analyzed the community composition and distribution of microorganisms (prokaryotes and eukaryotes) present in the Gerlache-Bismarck Strait, and their relationship with local oceanography. Sampling was conducted during the summer, the season when most research in this region occurs, allowing our results to be contextualized within previous studies. The Strait is influenced by multiple surface and intermediate-deep water masses and currents, each with different physical and chemical characteristics. These features play a key role in shaping microbial diversity and community assembly, with microorganisms being characteristic of a given water mass (their microbial fingerprint). Microorganisms from the fingerprint informed about the role of water masses in food webs and biogeochemical processes and therefore to its contribution to the functioning of the ecosystem. The microbial fingerprints reported here are based on a single season and location, with their temporal and spatial variability remaining to be explored in future studies.

### The oceanographic context

Water masses distribution and variability in the Gerlache-Bismarck Strait regulate several processes, including those affecting phytoplankton communities and food webs [42, 52], carbonate system and ocean acidification [18, 55, 56], and are key to understanding shelf-ocean interactions and the rates of tidewater glacier melting [34].

In this study, we observed that in surface waters (from 1 to 10 m), AASW and TBW dominated along the strait, which is consistent with previous studies [35, 37, 54]. We also detected the presence of GMW waters, extending from the fjords towards the center of the Gerlache Strait and in the Bismarck Strait. GMW originates from iceberg calving, basal melting and meltwater runoff [49–51], mainly during summer months [57]. Future increases in GMW are expected due to ongoing glacial loss accelerated by climate change, a process observed over recent decades [58]. This influx, which is also composed of meteoric water, may enhance primary productivity in coastal regions by supplying organic matter and essential elements, such as iron (Fe) and manganese (Mn) [59–62] when light is not a limiting factor for phytoplankton growth [63]. The Surface Water Thermal Front in the Gerlache-Bismarck Strait separates warmer surface waters in the northern part from the colder surface waters in the south and is regularly found close to the Schollaert Channel (front described in [35]). However, we detected this front northward near the entrance of the northern Gerlache Strait, indicating that the position of this front may vary interannually.

In the intermediate and deep waters (from 100 to 400 m), we identified TWW and mCDW water masses. TWW waters enter from the northern part of Gerlache-Bismarck Strait (e.g., [37, 54]), and mCDW water masses enter mainly from the southern part of the Gerlache-Bismarck Strait (e.g., [35, 53]). mCDW dominates the southern part of the Gerlache-Bismarck Strait (coinciding, e.g., with [35]), and its presence decreases northeastward and almost disappears in the middle part of the Gerlache Strait. In contrast, TWW dominates deep waters at the northern stations and decreases its influence on the Bismarck Strait. The deep waters of the Gerlache Strait are separated by the Sub-Pycnoclyne Front (described in [35]), whose position varies interannually from more northern to more southern locations [35]. In our case, as the mCDW practically disappeared in the middle-south part of the Gerlache-Bismarck Strait, we located the Sub-Pycnoclyne Front in this area near the Schollaert Channel.

In the Western Antarctic Peninsula and the Gerlache-Bismarck Strait, water masses and their physical–chemical properties are linked to specific biogeochemical characteristics [37, 64]. Silicate, nitrate, and phosphate had higher concentrations in the southern part of the Gerlache Strait because of the inflow of the CDW [37]. These high values recorded in February 2020 (silicate > 80 mmol kg^−1^; nitrate > 25 mmol kg^−1^) coincided with previous records in the same area by [37] and [65] during December and January 1995—1996.

Chlorophyll in the surface waters of the Gerlache Strait has been described as being high in various locations, including the northern part [28, 42, 55], the central part [66], and the southern part [28]. We observed high values linked to different entrances to the Gerlache-Bismarck Strait, and the highest values in the south. These contrasting results may be linked to temporal changes (intra-annual, inter-annual, or long-term changes) in phytoplankton dynamics [6, 19, 38, 67] and physical properties [36] that occur in the Western Antarctic Peninsula and the Gerlache-Bismarck Strait. The spatial distribution of phytoplankton groups in the Gerlache-Bismarck Strait and the links between phytoplankton and the environment are discussed below. The average chlorophyll concentration in the surface waters of the strait was 0.81 (mg m^−3^), with values up to 2.5 mg m^−3^. These values were comparable to those recorded by [68] in the same area during the same season (December 1995 and early January 1996) but lower than those recorded by [66] (December 1958 and January 1959) and [69] (from December 1986 to March 1987), reaching 25 mg Chl *a* m^−3^ in the northern part of the Gerlache Strait. The potential for phytoplankton biomass in the waters of the Southern Ocean is in the range of 25—50 mg Chl *a* m^−3^ (discussed in 26), due to high macronutrient availability. As the values we obtained were lower, the biomass obtained in our stations was likely constrained by light [63].

### Distribution of eukaryotes in the Gerlache-Bismarck Strait

Eukaryotic organisms in the Gerlache-Bismarck Strait have primarily been studied in summer and by microscopy, focusing mainly on phytoplankton (e.g., [27, 28, 44, 66, 70–73]). Additional studies have included nanoflagellates [74] and planktonic protists [75]. The same studies revealed that phytoplankton in the strait exhibit a basic assemblage (composed of flagellates, cryptophytes, dinoflagellates, prasinophytes, and haptophytes), particular blooms (which may be comprised of diatoms, cryptophytes, prasinophytes, or dinoflagellates), and north–south distributions (where cryptophytes dominate in the north and large diatoms in the south). However, due to global warming, diatoms seem to be decreasing in abundance, while cryptophytes seem to be increasing and moving southward, thus altering primary production, the efficiency of carbon sequestration and food webs [19, 38, 43, 76, 77]. The success of cryptophytes over diatoms is attributed to their capacity to survive in warm, stratified and low-salinity environments with high radiation [42, 77], conditions that are becoming more frequent in the Western Antarctic Peninsula.

This is, as far as we know, the first study which incorporates high-throughput DNA sequencing to study microbial eukaryotes in the Gerlache-Bismarck Strait, allowing for a detailed detection and identification of the different species present in the strait, including those that cannot be easily detected under a microscope, such as the picoeukaryotes. In surface waters, we recorded a heterogeneous distribution of Bacillariophyta (diatoms) and Cryptophyta all along the strait, suggesting that cryptophytes may be expanding their presence in southern waters, reaching further south than previously recorded, while diatoms may be experiencing a reduction in their favorable habitats as, at least in February 2020, Bacillariophyta were principally restricted to areas with glacial influence (e.g., Andvord and Flandres Bays).

Information on eukaryotes in the deep waters of the Gerlache-Bismarck Strait is still scarce, as most studies have focused on phytoplankton in surface waters. In this study, we observed that the diversity of eukaryotes decreased with depth, where diatoms and cryptophytes decreased their presence in deep waters, while other microorganisms, such as dinoflagellates and fungi, increased their presence. We found that dinoflagellates were more abundant at northern stations, coinciding with the results obtained in [74]. In contrast, fungi were especially abundant in the southern stations, particularly in deep waters. Information on marine fungi in marine polar systems is still limited, but previous studies have reported high fungal abundance in Arctic marine waters [78], which aligns with our observations in Antarctica. We also observed that fungi were highly diverse, consistent with previous studies on cultivable Antarctic fungi [79]. In Antarctica, fungi have been detected in deep waters, including meso- and bathypelagic waters [80, 81]. The higher dominance of fungi in deep waters that we observed in the southern part of the Gerlache-Bismarck Strait may be linked to processes of decomposition which dominate in marine deep waters, and to the “fungal shunt” [82].

### Distribution of prokaryotes in the Gerlache-Bismarck Strait

To the best of our knowledge, this is the first comprehensive description of bacterial diversity in the Gerlache-Bismarck Strait. Our approach also includes size-fractionation, encompassing microbial communities and particles from 0.2 to 200 µm. Although in our results the composition of AVSs in the larger fractions may appear comparable to those of smaller size-fractions, these communities likely represent only a minor component of the total prokaryotic assemblage, as abundances decrease with the size-fraction, with proportions of < 10% in the size fractions > 3 μm [83].

Our results revealed that, during summer, surface waters (1 and 10 m) were dominated by the phyla Proteobacteria (mainly Alpha- and Gamma-), Bacteroidota (mainly Flavobacteria), and Planctomycetota, whereas intermediate and deep waters (100—400 m) were dominated by Proteobacteria (mainly Alphaproteobacteria) and Acidobacteriota. Similar groups were found to be dominant in waters close to the Gerlache Strait (in the Bransfield Strait and Bismarck Strait) [84–88] and in open waters from the Southern Ocean (reviewed in [1, 2, 4, 40, 41, 84, 89, 90]).

In our samples, we observed a higher presence of Planctomycetota than in previously mentioned studies. Planctomycetes are more dominant in larger size fractions [83]. As in this study we incorporated three size fractions ranging from 0.2 to 200 µm, our size fractionation approach favored the detection of this group compared to previous studies in Antarctica, which in general did not consider different size fractions. Among archaea, we found that the most abundant group was Crenarchaeota, coinciding with previous works in the same area [45, 46]. In our study, Crenarchaeota were primarily found below 100 m and were mainly associated with the CDW water mass, as was observed in previous studies [45, 89].

### Links between microbial communities and environmental variables

Multiple studies have shown that marine microbial communities in Antarctica are structured by location, depth, and physical and biogeochemical variables [2–4, 20, 40, 41, 90, 91]. In particular, among the physical and biogeochemical variables explored (see e.g., [85–87, 92–96]), temperature is a major driver of Southern Ocean microbial communities (reviewed in [2, 40]). In this study, we observed that in the Gerlache-Bismarck Strait, major drivers include pH and nitrate, proxies for local primary production [97, 98] and CDW intrusions in this area [37, 65]. The same variables were relevant for microbial communities in the nearby area, the Bransfield Strait [86, 99–101], where nitrate was key in determining microbial communities below the photic layers, as we observed in the Gerlache-Bismarck Strait.

Eukaryotes and prokaryotes respond differently to environmental factors [95]. An example of this are the different microorganisms found in two contrasting bays in the Gerlache Strait: Wilhelmina and Andvord Bays. In Wilhelmina Bay, more than half of the glaciers are in retreat and phytoplankton are recurrently dominated by cryptophytes [44]. In contrast, in Andvord Bay, none of the glaciers are currently retreating (reviewed in 44) and diatoms tend to dominate [43]. The higher temperatures that we recorded in Wilhelmina (> 3ºC) represented a marine heatwave [47] and most likely enhanced the role of heterotrophic bacteria, with subsequent impacts on the carbon cycle, as has been previously theorized [102]. In Andvord Bay, we observe the presence of diatoms (Bacillariophyta), similarly to what was observed in previous surveys in the same bay [43, 44, 72, 103].

Besides environmental factors, in this study we tested the effect of water masses in structuring Gerlache-Bismarck Strait marine communities. Despite the fact that environmental variables measured in this study explained a large part of the distribution of microorganisms in the Gerlache-Bismarck Strait, water masses were a more prominent driver of the distribution and assembly of microbial communities. Water masses (as explanatory variables) encompass not only the effect of local biogeochemical and physical parameters on selecting microbial communities, but, as marine waters are in constant movement, water masses also encompass the effect of historical processes acting on microorganisms during their oceanic transport (this is, their legacy effects [104]). Dispersion and selection are two relevant process shaping microbial communities elsewhere [105], and also in Antarctica [84, 95], and oceans are dynamic fluids with constant motion where water masses, together with fronts and currents, determine their structure and function. Altogether, our results indicate that microbial communities may be shaped not only by local environmental conditions but also by shifts occurring during their transport through water masses, as has been predicted by the Microbial Conveyor Belt [106]).

### The microbial fingerprinting of the water masses from the Gerlache-Bismarck Strait

In each of the five water masses, we identified a characteristic microbial community (the microbial fingerprint) defined by a particular community assembly, a characteristic network configuration, specific dominant taxa, indicator microorganisms, and a characteristic core microbiome. The microbial fingerprint of each water mass is detailed in the Results section. The microbial fingerprints of this work had been characterized from samples collected during the summer in a specific area, and their variability should be explored further in future studies. We expect the microbial fingerprint to vary over time, particularly in surface waters, which are more dynamic and in constant exchange with the atmosphere and terrestrial inputs. Among indicator microorganisms, eukaryotes may be more relevant for defining the microbial fingerprint, as many (generally phytoplankton) are cultivable (so there is more information available about them) and are more sensitive to dispersal limitation and environmental selection than prokaryotes (i.e., they disperse less and are more influenced by the characteristics of the environment, and thus the water mass, than prokaryotes), as has been described in Antarctica [95] and elsewhere [107–109].

AASW waters, found in surface waters in the central part of the Gerlache-Bismarck Strait, include the eukaryotic phylum Cercozoa (*Cryothecomonas aestivalis*, Order Cryomonadida) as an indicator microorganism. This nanoflagellate, which feeds on diatoms [110], exhibits a bipolar distribution. Yet, information about this species in Antarctica is scarce. *C. aestivalis* has been detected in the Antarctic Peninsula [92, 111, 112] and is abundant in the Bransfield Strait [112], but there are no records of its presence in the Gerlache Strait, likely due to biases in the microscopy-based methods applied in prior surveys. No previous studies have linked this species to AASW waters, but this species has been associated with sea ice and glacial melt water mixing in seawater from both polar regions [92, 111], which explains its presence in AASW waters.

In TBW waters, situated in surface waters throughout the Gerlache-Bismarck Strait, the Cryptophyta species *Geminigera cryophila* is an indicator microorganism. *G. cryophila* is a fully described cryptophyte species isolated from Antarctic waters and probably has an Antarctic circumpolar distribution [113]. *G. cryophila* is a mixotrophic nanoflagellate, and molecular analysis revealed that this species, in particular, dominates over other Cryptophyta species, at least during summer in the Bransfield Strait [112] and along the Western Antarctic Peninsula [114]. Cryptophyta have been reported to be highly abundant in TBW waters from the Bransfield Strait [101], which is consistent with our observations. Members of the phylum Cryptophyta have increasing abundances in the Western Antarctic Peninsula due to climate change [77].

GMW is associated with melting water coming from glaciers and we found this water mass throughout the Gerlache Strait, with the Haptophyta *Chrysochromulina simplex* as an indicator microorganism. Haptophytes are part of the basic assemblage of phytoplankton in the Gerlache Strait, as described by [44]. The haptophyta *C. simplex* is a nanoplankton phototrophic species, and some studies have suggested that it might be a mixotroph [115]. It has mainly been described in the Arctic and, in Antarctica, *C. simplex* has been found in coastal surface waters from the Bransfield Strait [116], and reaching high abundances [112]. There is little information about the most favorable environments for *C. simplex*, but in the Arctic, this species appears to be tolerant to a wide range of temperature and salinity conditions [117]. This feature probably contributes to the adaptation of this species to the meltwater pulses that constitute the GMW water mass.

In TWW waters, found between 100 and 400 m, indicator microorganisms correspond to the phylum Proteobacteria and to the families Nitrosomonadaceae, Rhodanobacteraceae, and Oxalobacteraceae. Information about these families in the marine waters of Antarctica is sparse, but it is known, for example, that Rhodanobacteraceae are found abundantly in terrestrial environments from the Bransfield Strait in the soil and penguin colonies [118–120]. This suggests that some representatives of this family may be transported from land to the marine environment, potentially through runoff when TWW passes near the tip of the Antarctic Peninsula where there are large colonies of penguins [121, 122]. Therefore, rather than representing taxa intrinsically associated with the TWW microbiome, Rhodanobacteraceae could reflect the interaction of these waters with surrounding terrestrial environments, acting as indicators of the passage of TWW near the Antarctic Peninsula when it goes through the Bransfield Strait.

The CDW water mass, situated below 100 m and extending from Bismarck towards Gerlache Strait includes archaea as indicator microorganisms. In particular, Crenarchaeota from the class Nitrososphaeria and Nitrosopumilaceae family (also known as Thaumarchaeota or Marine Group I archaea). Nitrosopumilaceae are aerobic marine ammonia-oxidizing archaea [123] that frequently appear through the marine water column, especially at subsurface and mesopelagic water depths, since their metabolism is inhibited by light. In Antarctica, Nitrosopumilaceae have been found to be abundant in the deep waters of the Antarctic Peninsula [86], including the Gerlache Strait [45], especially during the summer [89]. In winter, there are evidences that Nitrososphaeria also occur at surface waters [124]. We observed that Nitrosopumilaceae species were characteristic of CDW waters during the summer season, where they most likely play a key role in the nitrogen cycle, concretely on the oxidation of ammonium to nitrite, the first step of nitrification. Besides, we detected in CDW waters the presence of some nitrite-oxidizing bacteria such as *Nitrospira*, which could be participating in the complete nitrification.

### The relevance of the microbial fingerprint of water masses

Links between microorganisms and water masses have been evident for decades: early studies proposed microorganisms as indicators of hydrographic phenomena by measuring variations in microbial abundances through colony-forming unit counts [125]. The method contributed to mapping the circulation and distribution of water masses in the Indian, Pacific, and Atlantic Oceans [126–128]. Contemporary studies, boosted by advances in molecular technology and high-throughput sequencing, have shown that microbial diversity is determined by oceanic structures, such as water masses and fronts [108, 129–134]. These structures directly influence the metacommunity organization of microbial communities and also ecological processes such as dispersion and selection and thus, the biogeography of marine microorganisms [104–106, 135].

In polar regions, earlier studies in the Arctic explored the links among ocean structures and marine microbes [136–139]. In Antarctica, multiple and recent studies have linked microbial communities to specific water masses (e.g., [81, 89, 140–142), investigated their transport through water masses advection [143], and revealed that bacterial community composition is more strongly influenced by dispersal mechanisms through water masses than biogeochemical parameters [144]. In this study, we found that water masses are a crucial factor determining the microbial community composition.

Building on previous evidence that distinct water masses host characteristic microbiomes and to gain deeper insight into the links between water masses and marine microorganisms, we introduced the concept of the microbial fingerprints of water masses and proposed a methodology to explore and define it. Applied to our data, the fingerprint described in this study revealed the contribution of water masses to the Gerlache-Bismarck Strait ecosystem, as the approach effectively identified taxa reflecting the ecology and biogeochemistry processes taking place in the region. For example, *Cryothecomonas aestivalis*, a key species in surface water masses (AASW), feeds on diatoms and thus directly shapes the food web. Another example is the archaea Nitrosopumilaceae, found predominantly in deeper water masses (CDW), which plays a key role in nitrogen cycling and fertilization of the Gerlache-Bismarck Strait.

Our results are consistent with those reported for similar water masses in other regions. However, the microbial fingerprint we describe corresponds to a specific season and area, and its extrapolation to other regions and seasons containing the same water masses requires further studies. Defining a single, universal microbial fingerprint of a specific water mass would require a much larger dataset, encompassing diverse seasons, regions, and unmixed waters. The methodology we propose can be applied to the mentioned meta-analysis, and also to other more specific and regional studies, aiming to elucidate the role water masses in ecological and biogeochemical processes mediated by microorganisms.

In ecologically relevant and climatically sensitive areas, such as the Gerlache-Bismarck Strait, understanding microbial fingerprints linked to water masses can provide critical insights into the present and future functioning of the ecosystem. Any changes in marine systems and ocean circulation, such as those triggered by climate change [145], will affect those microorganisms [146] and change their distribution [147], which in turn may drastically change marine ecosystem dynamics.

## Conclusions

The Gerlache-Bismarck Strait is a productive area that is an important feeding ground for krill, penguins, and whales. In this study, we observed that each water mass had a specific microbial community structure, which we named the “microbial fingerprint of water masses”. The microbial fingerprint of each water mass in the Gerlache-Bismarck strait provides information on the role of the water masses on the biogeochemistry and trophic food webs in this area. Regarding protective measures in the Gerlache Strait and other areas of Antarctica, it is essential to consider not only the macroscopic diversity but also microorganisms and the hydrography they are linked to, along with the expected changes in seawater hydrographic properties due to global change (such as rising temperatures, ocean acidification, freshening as well as changes in currents and fronts), as these will directly affect microbial communities and, in turn, the entire ecosystem functioning.

## Methods

### Sampling

Sampling was carried out in February 2020 (summer in the Southern Hemisphere), onboard the R/V Karpuj. A transect was performed along the full Gerlache-Bismarck Strait (Fig. 2), from northeast off Brabant Island (64,32º S, 61,80º W) to southwest off Anvers Island (64,97º S, 64,52º W), including 13 oceanographic stations (Fig. 2). At each oceanographic station, a CTD (*SBE 25 plus* by Seabird®) was used to assess the oxygen concentration (mg L^−1^) and saturation percentage (% sat.), temperature (°C), potential temperature (°C), and salinity (practical salinity) (see details in [98]). Additionally, seawater from the oceanographic stations was sampled at 1, 10, 100, 200, 240, and 400 m using 5 L Niskin and 10 L Go-Flo bottles in order to study the biogeochemistry and microbial communities (Fig. 2). The measured biogeochemical variables included chlorophyll concentration (mg m^−3^), in situ pH, dissolved silicate (µmol L^−1^), dissolved nitrate (µmol L^−1^), dissolved phosphate (µmol L^−1^), concentration of suspended inorganic particulate matter (ISM, mg L^−1^) and concentration of suspended organic particulate matter (OSM, mg L^−1^). The fraction of organic matter present in suspended matter was defined as %OSM. Biogeochemical variables were sampled and analyzed as per [98] except for ISM and OSM that were processed following [148].

To determine microbial diversity (including microscopic prokaryotes and eukaryotes), 9 L of seawater were filtered using a peristaltic pump (Masterflex™ L/S™, Fisher Scientific). To diminish the impact of clogging and dislodging particles from the filters, we filtered at a very low speed and pressure (flow rate of 50 mL min⁻^1^) and replaced the filters when needed. The filters were submerged in a nucleic acid preserving solution (*RNAlater*, Invitrogen), rapidly froze at −20ºC onboard, and stored at −80 ºC until DNA extraction. The filtration system included a 200 μm mesh followed by 3 polycarbonate filters (pore sizes: 20 μm, 3 μm, and 0.2 μm) arranged sequentially. Using this procedure, we obtained three size fractions corresponding to three sizes of plankton and marine particles: picoplankton and picoparticles (0.2–3 μm), which include free-living prokaryotes and eukaryotes; nanoplankton and nanoparticles (3–20 μm), which consist mainly of larger eukaryotic microorganisms and prokaryotes associated with particles or protists; and microplankton and microparticles (20–200 μm), which include larger eukaryotic microorganisms and prokaryotes associated with larger particles or protists.

### Characterization of water masses mixing

The principal characteristics of the different water masses present in the Gerlache-Bismarck Strait (i.e., AASW, GMW, TBW, TWW and CDW) were obtained from: [33–35, 37, 52–54]. The contribution of each water mass to the seawater properties at each station and depth was determined by applying the mixing triangle method [149] as described in [150]. The method involves analyzing the distribution of water masses using T-S (Temperature-Salinity) diagrams for the considered oceanographic stations. According to this approach, to calculate the mixing ratio percentages, it is essential to define the distinct thermohaline indices characteristic of each water mass within a given triangle (Additional file 2: Table S2 and Figure S23). Finally, for each station and depth, the contribution (in %) of each water mass was obtained, and the dominant water mass at each station and depth was determined as the water mass contributing with a higher % to that particular mixing.

### DNA extraction, Sequencing and Sequence processing

This work includes the study of prokaryotic (bacteria and archaea) and eukaryotic microorganisms through DNA analysis, using primers targeting the 16S rRNA and 18S rRNA genes described by [151]. DNA extraction, library preparation, and sequencing were performed at the IMR (Integrated Microbiome Resource, Halifax, Canada), and protocols were carried out as originally described by [78] and updated as in [152]. DNA extraction was performed using the *QIAGEN PowerSoil Pro kit* on *QIAcubeHT*, and sequencing was performed on an *Illumina MiSeq* platform with a v3 600 cycle kit (2 × 300 bp). Computational analyses of demultiplexed reads were performed based on *QIIME2* [153] and *DADA2* [154] along with a custom bioinformatic pipeline incorporating elements from [155], and [156]. Briefly, primer sequences were removed with *Cutadapt* [157], discarding any sequence pairs that did not contain the forward and reverse primers. Mixed amplicon sequences were then split into 16S and 18S rRNA genes pools using *bbsplit.sh* from the *bbtools* package (https://jgi.doe.gov/data-and-tools/software-tools/bbtools/bb-tools-user-guide/) against curated 16S/18S databases (https://osf.io/e65rs/) derived from *SILVA 132* [158] and *PR2* [159]. Subsequently, parallel denoising of 16S rRNA gene and 18S rRNA gene sequences were conducted to generate ASV (Amplicon Sequencing Variants) tables for subsequent analysis. For both 16S and 18S rRNA gene sequences, the forward read was truncated to 220 bp and the reverse read to 180 bp. 16S reads were merged prior to denoising, whereas for 18S the forward and reverse read were concatenated since sequence lengths are not sufficient for full overlap with 18S rRNA for this primer region. Sequences derived from Metazoa, mitochondrial DNA, and chloroplast DNA were excluded during the processing steps.

### Data analysis

Data analysis was performed using R software (v4.3.2) within RStudio interface [160], and environmental variables together with the geography were visualized using ODV software (Ocean Data View, v5.6.3). To analyze microbial communities, the ASV table was first randomly subsampled (with the *rrarefy* function, *vegan* package) to the number of reads present in the sample with the lowest number of reads (*n* = 4041), reducing from 111 samples to 107 and a total of 13,548 ASVs.

The diversity of each sample was calculated with different diversity indices, those were: the total number of species per sample or richness (index S.Obs), the Shannon index (H’), the Simpson index (D’), and the Pielou index (J’) (evenness). The formulas used for calculating the indices were implemented in R, following [161] and [162]. The specific formulas used are shown in Additional file 2: Table S9. Subsequently, correlation analysis (Additional file 2: Figure S7) was performed among the different indices using the *cor* and *cor.test* functions (*base* package), where we applied the Spearman correlation method. Results were visualized using the *ggpairs* function (*GGally* package), revealing that all indices were generally correlated (R^2^ ≥ 0.4; R > 0.5; *p*-value < 0.001). Among all indices, we chose the richness index (S.Obs) for downstream diversity analyses because of its conceptual simplicity. To determine whether diversity in terms of richness was statistically similar or different among the levels within each factor (size fractions, depths, stations), a Kruskal–Wallis test was conducted using the *kruskal.test* function from the R *base* package.

Low abundant ASVs (also referred to as “rare”), were defined as those representing < 1% of the total abundance of all sequences (considering all samples). Taxonomic composition of microbial communities was visualized with barplots (*tidyverse* and *ggplot2* packages), by selecting the most abundant taxa, at different taxa levels (Phylum, Class and Order levels). The other taxa, together with the rare, were represented as “Others”.

To assess the influence of different factors (such as water fronts, water masses, stations, depths, size fractions and zones) on microbial communities, a series of non-parametric permutational multivariate analysis of variance (PERMANOVA) tests with 999 permutations were conducted (*adonis2* function, *vegan* package). Additionally, a PERMANOVA with marginal effects analysis (by = "margin") was applied to test the overlap among the factors depth and water mass. The effect of the environment (explanatory variables) on microbial communities (response variables) were assessed by considering two types of explanatory variables: environmental variables (including physical and biogeochemical variables) and water masses mixing (i.e., the contribution, in percentage, of each water mass). A simple set of explanatory variables was obtained by first discarding redundancies (we applied Spearman correlation method and the thresholds: R > 0.9; *p*-value < 0.001) (Additional file 2: Figure S6). Then the Bioenv procedure was applied (*bioenv* function, *vegan* package), which identifies the smallest subset of explanatory variables that correlates maximally with microbial community data. For this, distance matrices were constructed (for explanatory variables using Euclidean distances and for response variables using Bray–Curtis distances) and the Spearman-rank-based correlation method was applied. Finally, the effect of selected environmental variables on microbial communities were visualized in a distance-based redundancy analysis (dbRDA) (*capscale* function, *vegan* package)*.* To assess the significance of the selected variables on microbial communities, a permutational ANOVA test was conducted (*anova.cca* function, package *vegan*), with 999 permutations.

To gain a deeper understanding of the structure of the microbial communities present in different water masses, we examined indicator microorganisms, the core microbiome and co-occurrence networks. Indicator species (usually defined as those microorganisms that are abundant in a particular group of samples, and nearly absent in other groups of samples), were identified at the ASV level for each water mass defined as dominant. This was performed using the *multipatt* function (*indicspecies* R package) with 9999 permutations and selecting r.g as species-site group association function. p-values were adjusted post hoc for multiple comparisons using the *p.adjust* function with the False Discovery Rate (FDR) method, to reduce the false positive rate [163]. After identifying the indicator ASVs, an additional filtering step based on relative abundance was applied: Indicator taxa for a specific water mass were only retained if they were either exclusive or showed a higher relative abundance in the samples where that water mass was dominant, compared with the other samples in which those microorganisms appeared. Indicator microorganisms were visually represented at the ASV level in heatmaps and at the Phylum and Class levels in dotplots (option *geom_point*, *ggplot* function, *ggplot2* package). Finally, the core microbiome (i.e., those microorganisms that appear consistently in samples from a given group, regardless of what microorganisms are in other groups) of the water masses was determined using the function *core_members* from the *phyloseq* package. The core microbiome was calculated at the ASV level, considering a minimum abundance of 1%, and a prevalence of 75% among samples of the same water mass. These thresholds align within the range commonly used in similar studies [164]. Co-occurrence networks were explored with the *igraph* R package for network analysis and visualizations. Only those ASVs that appeared in at least three samples were considered. Hypothetical interactions among ASVs were detected through correlations. For this, a correlation matrix between the ASVs was generated by applying the Spearman correlation method and those correlations with a threshold of R > 0.65 were selected. Different co-ocurrence network parameters were calculated (*igraph* package) including number of nodes and edges (*gorder* and *gsize* functions, respectively), average path length (*mean_distance* function), modularity (*cluster_walktrap* and *modularity* function), average degree (*mean* function, *base* package), clustering coefficient (*transitivity* function), and density (*edge_density* function). Networks were visualized using the functions *graph_from_adjacency_matrix*, *delete.vertices*, and *graph_from_data_frame*.

## Supplementary Information

Additional file 1. Constituent parts: Abstract and Title in Spanish.

Additional file 2. Constituent parts: Supplementary Figures and Tables. Table S1. Water masses identified in the Gerlache-Bismarck Strait. Table S2. Water masses and their thermohaline indices. Table S3. Details about the sampling stations. Figure S1. Environmental variables considered in this study represented as vertical profiles. Figure S2. Environmental variables at 1 m depth. Figure S3. Environmental variables at 10 m depth. Figure S4. Environmental variables at 100 m depth. Figure S5. Environmental variables at intermediate and deep depths. Figure S6. Correlations (Spearman method) among the different environmental variables included in this study. Figure S7. Results of the correlations (Spearman method) conducted between the different alpha diversity indices. Figure S8. Dotplot showing richness values (number of ASVs) along the Gerlache-Bismarck Strait, for each station, size fraction and depth. Figure S9. Boxplot showing richness values (number of ASVs) for each size fraction and depth. Figure S10. Barplot depicting the microbial community composition (represented at the Phylum level) of the Gerache-Bis marck Strait, at each depth and size-fraction. Figure S11. Barplot depicting the microbial community composition (represented at the Class level) of the Gerache-Bismarck Strait, at each depth, and size fraction. Figure S12. Barplot showing the microbial community composition (represented at the Phylum level) of the Gerache-Bismarck Strait, at each station, depth, and size-fraction. Figure S13. Barplot showing the microbial community composition (represented at the Class taxonomic level) of the Gerache-Bismarck Strait, at each station, depth, and size fraction. Figure S14. Heatmaps revealing the relative abundance of Eukaryotes (at Phylum and Class level) at different depths and at each size fraction. Figure S15. Heatmap revealing the relative abundance of Prokaryotes (at Class and Order level) at different depths and at each size fraction. Figure S16. Distance-based redundancy analysis (dbRDA) showing the influence of (A, B) biogeochemical variables and (C, D) water masses on microbial community composition. Figure S17. Distance-based redundancy analysis (dbRDA) showing the influence of biogeochemical variables on microbial community composition, for each size-fraction. Figure S18. Distance-based redundancy analysis (dbRDA) showing the influence of water masses on microbial community composition, for each size-fraction. Figure S19. Bar plot showing the microbial community composition (represented at the Class level) in each water mass, and size fraction. Figure S20. Bar plot showing the microbial community composition (represented at the Order level) in each water mass, and size fraction. Figure S21. Heatmap revealing the relative abundance of Eukaryotes (at Phylum and Class level) for each water mass. Figure S22. Heatmap revealing the relative abundance of Prokaryotes (at Class and Order level) for each water mass. Table S4: List of indicator microorganisms for each water mass. Table S5: List of the core microbes of each water mass. Table S6: DNA sequence of the indicator microbes. Table S7: DNA sequence of the core microbes. Table S8. Different metrics of co-occurrence networks calculated for each of the water masses considered in this study. Figure S23. Temperature-Salinity (T-S) diagram with water mass cores included. Table S9. Formulas used for calculating alpha diversity.

## Data Availability

The datasets generated and/or analysed during the current study are available. DNA sequencing data (16S and 18S rDNA amplicon sequences) are available in the NCBI repository under accession ID PRJNA1259996 [165] and environmental and oceanographic data are available in the PANGAEA repository [166].

## Abbreviations

ACC/APCC: Antarctic Circumpolar Current / Antarctic Peninsula Coastal Current
AASW: Antarctic Surface Waters
ASVs: Amplicon Sequence Variants
CDW: Circumpolar Deep Waters
CTD: Conductivity–Temperature–Depth
DNA: Deoxyribonucleic acid
GMW: Glacially Modified Waters
HNLC: High-Nutrient, Low-Chlorophyll regions
HSSW: High Salinity Shelf Water
ISM: Inorganic Suspended Particulate Matter
LCDW: Lower Circumpolar Deep Water
mCDW: Modified Circumpolar Deep Water
mHSSW: Modified High Salinity Shelf Water
OSM: Organic Suspended Particulate Matter
SPF: Sub-Pycnocline Front
STF: Surface Water Thermal Front
TBW: Transitional Zonal Waters influenced by the Bellingshausen Sea
TWW: Transitional Zonal Waters influenced by the Weddell Sea
UCDW: Upper Circumpolar Deep Water
