# Supplementary material for: Microbial fingerprinting of marine water masses in an Antarctic and hydrographically complex area
Source: BMC Biol. 2026 May 23;24:144. doi: 10.1186/s12915-026-02621-8 (PMC13295705; doi:10.1186/s12915-026-02621-8)
Supplement: Supplementary file 1 — Additional file 1. Constituent parts: Abstract and Title in Spanish. [file 12915_2026_2621_MOESM1_ESM.pdf]

Additional File 1:
Abstract in Spanish

Publisher's note:
This translation in Spanish was submitted by the authors and we reproduce it as supplied. It has not been peer reviewed. Our editorial processes have only been applied to the original abstract in English, which should serve as reference for this article. This translated abstract is published under the same licence as the article.

TITLE/TÍTULO:

Huella microbiana de las masas de agua marinas en una región antártica hidrográficamente compleja

ABSTRACT/RESUMEN:
Antecedentes: Los microorganismos se encuentran ampliamente distribuidos en los ecosistemas marinos. Sin embargo, todavía se conoce poco sobre la influencia de las masas de agua en su distribución, especialmente en ambientes extremos como la Antártida. Este estudio examina las comunidades microbianas en el Estrecho Gerlache-Bismarck (Antártida), una región caracterizada por tener una hidrografía compleja. En este estudio se analizó la diversidad de procariotas y eucariotas y su vínculo con diversas variables oceanográficas y biogeoquímicas. Para ello, se recolectaron muestras de agua entre 1 y 400 m de profundidad, y se consideraron tres fracciones de tamaño: pico- (0,2–3 µm), nano- (3–20 µm) y micropartículas (20–200 µm).

Resultados: Los resultados revelaron que las características de las masas de agua son uno de los principales determinantes del ensamblaje de las comunidades microbianas. Las masas de agua superficiales detectadas en este estudio incluyeron: Aguas Superficiales Antárticas (AASW), Aguas Modificadas por Glaciares (GMW) y Aguas Zonales Transicionales influenciadas por el Mar de Bellingshausen (TBW). En capas intermedias y profundas se detectaron: Aguas Zonales Transicionales influenciadas por el Mar de Weddell (TWW) y Aguas Profundas Circumpolares (CDW). Este estudio aporta evidencia directa del estrecho vínculo entre los microorganismos y las masas de agua, y muestra que las comunidades microbianas marinas están estructuradas no solo por las condiciones locales, sino también por la circulación oceánica. Además, en este estudio desarrollamos el concepto de "huella microbiana de las masas de agua", mostrando que las comunidades microbianas pueden servir como indicadores ecológicos. El desarrollo de este enfoque proporciona, además, un marco de estudio transferible a otros sistemas marinos.

Conclusiones: Las huellas microbianas aportaron información sobre el papel de las masas de agua en la biogeoquímica y en las redes tróficas de esta región (por ejemplo, las arqueas oxidantes de amonio Nitrosopumilaceae en las CDW y el fotótrofo *Chrysochromulina simplex* en las GMW). Esta información resulta útil para predecir cómo cambios futuros en la circulación oceánica y en la distribución microbiana podrían alterar los servicios ecosistémicos de esta región crítica.
