# Supplementary material for: Microbial fingerprinting of marine water masses in an Antarctic and hydrographically complex area
Source: BMC Biol. 2026 May 23;24:144. doi: 10.1186/s12915-026-02621-8 (PMC13295705; doi:10.1186/s12915-026-02621-8)
Supplement: Supplementary file 2 — Additional file 2. Constituent parts: Supplementary Figures and Tables. Table S1. Water masses identified in the Gerlache-Bismarck Strait. Table S2. Water masses and their thermohaline indices. Table S3. Details about the sampling stations. Figure S1. Environmental variables considered in this study represented as vertical profiles. Figure S2. Environmental variables at 1 m depth. Figure S3. Environmental variables at 10 m depth. Figure S4. Environmental variables at 100 m depth. Figure S5. Environmental variables at intermediate and deep depths. Figure S6. Correlations (Spearman method) among the different environmental variables included in this study. Figure S7. Results of the correlations (Spearman method) conducted between the different alpha diversity indices. Figure S8. Dotplot showing richness values (number of ASVs) along the Gerlache-Bismarck Strait, for each station, size fraction and depth. Figure S9. Boxplot showing richness values (number of ASVs) for each size fraction and depth. Figure S10. Barplot depicting the microbial community composition (represented at the Phylum level) of the Gerache-Bis marck Strait, at each depth and size-fraction. Figure S11. Barplot depicting the microbial community composition (represented at the Class level) of the Gerache-Bismarck Strait, at each depth, and size fraction. Figure S12. Barplot showing the microbial community composition (represented at the Phylum level) of the Gerache-Bismarck Strait, at each station, depth, and size-fraction. Figure S13. Barplot showing the microbial community composition (represented at the Class taxonomic level) of the Gerache-Bismarck Strait, at each station, depth, and size fraction. Figure S14. Heatmaps revealing the relative abundance of Eukaryotes (at Phylum and Class level) at different depths and at each size fraction. Figure S15. Heatmap revealing the relative abundance of Prokaryotes (at Class and Order level) at different depths and at each size fraction. Figure S16. Distanc [file 12915_2026_2621_MOESM2_ESM.pdf]

Additional File 2:
SUPPLEMENTARY Material

for

Microbial Fingerprinting of Marine Water Masses in an Antarctic and Hydrographically Complex Area

BMC Biology
2026

AUTHORS:

Mireia Mestre, Alicia Prior, Camila Marín-Arias, Daniel R. Rodríguez-Solís, Rafael Laso-Pérez, Emilio Alarcón, Valeska Vásquez-Lepio, Humberto E. González, Ramiro Logares, Jesse McNichol, Jed Fuhrman, Camila Fernandez, Mark J. Hopwood, Juan Höfer

Supplementary Table 1. Water masses identified in the Gerlache–Bismarck Strait, along with their corresponding abbreviations.

**Abbreviation**	**Oceanographic feature**
STF	Surface water Thermal Front
SPF	Sub-Pycnocline Front
AASW	Antarctic Surface Waters
TBW	Transitional Zonal Waters with Bellingshausen Sea influence
GMW	Glacially Modified Waters
TWW	Transitional Zonal Waters with Weddell Sea Influence
CDW	Circumpolar Deep Waters

Supplementary Table 2. Water masses and their thermohaline indices (*T, S*). Values were obtained from: (1) Dierssen, HM, Smith, RC, Vernet, M. 2002. Glacial meltwater dynamics in coastal waters west of the Antarctic peninsula. *Proceedings of the National Academy of Sciences*, 99: 1790-1795; (2) García, MA, Castro, CG, Ríos, AF, Doval, MD, Rosón, G, Gomis, D, López, O. 2002. Water masses and distribution of physico-chemical properties in the Western Bransfield Strait and Gerlache Strait during Austral summer 1995/96. *Deep Sea Research Part II: Topical Studies in Oceanography*, 49: 585-602; (3) Parra, RRT, Laurido, ALC, Sánchez, JDI. 2020. Hydrographic conditions during two austral summer situations (2015 and 2017) in the Gerlache and Bismarck straits, northern Antarctic Peninsula. *Deep Sea Research Part I: Oceanographic Research Papers*, 161: 103278

**Water masses**	**Temperature (ºC)**	**Salinity (practical salinity)**	**References**
Glacier Modified Waters (GMW)	3.10-3.20	32.50-32.70	1
Antarctic Surface Water (AASW)	1.00-2.50	33.36-34.11	2,3
Transitional Bellingshausen Water (TBW)	0.10-0.70	33.66-33.91	2
Transitional Weddell Water (TWW)	-0.70-0.30	34.50-34.60	2
Circumpolar Deep Water (CDW)	1.30-1.70	34.50-34.70	2,3

.

Supplementary Table 3. Details about the sampling stations.

Station	Depths [m]	Bottom Depth [m]	Dominant water mass	Sampling date [dd-mm-yyyy]	Latitude [degrees North]	Longitude [degrees East]
01	1, 10, 100	690	GMW	09-02-2020	-64,622283	-62,227017
03	1,10,100, 400	690	TBW	10-02-2020	-64,51105	-62,378617
06	1	617	AASW	10-02-2020	-64,5961	-62,678083
09	1	542	TBW	10-02-2020	-64,781083	-62,745417
11	1, 10, 100, 200	232	TBW	11-02-2020	-64,7046	-62,94385
15	1	258	TBW	11-02-2020	-64,845367	-63,203883
18	1	296	TBW	11-02-2020	-65,051633	-63,25855
20	1, 10, 100, 240	249	TBW	11-02-2020	-65,00665	-63,44775
23	1	347	AASW	12-02-2020	-64,911633	-63,697133
26	1, 10, 100, 400	474	GMW	12-02-2020	-64,829983	-64,075333
29	1, 10, 100, 400	1013	GMW	12-02-2020	-64,973433	-64,523633
31	1, 10, 100, 400	982	GMW	16-02-2020	-64,3223	-61,803267
34	1	795	TBW	16-02-2020	-64,425067	-62,092117

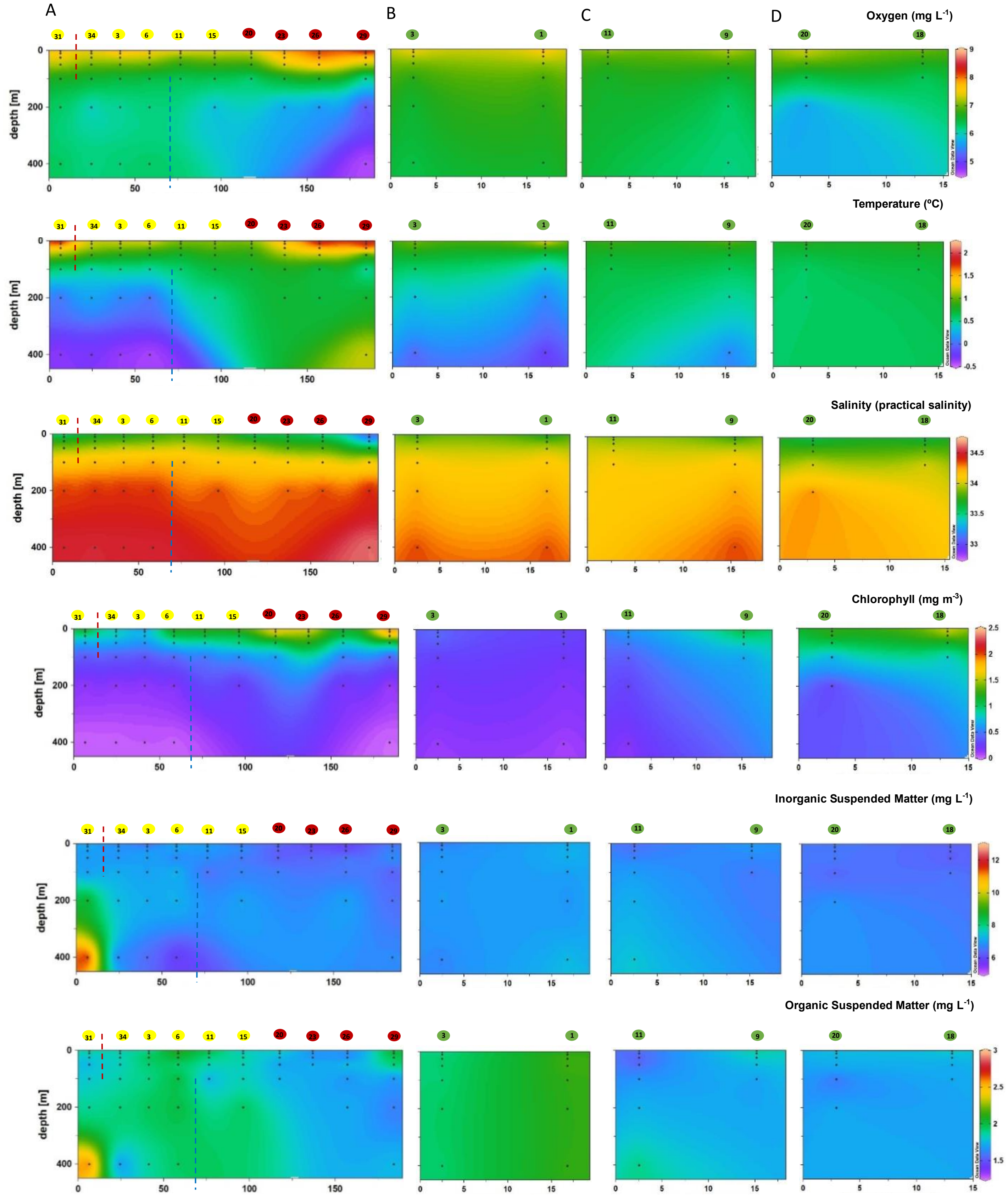
A
B
C
D
Oxygen (mg L-1)
Temperature (ºC)
Salinity (practical salinity)
Chlorophyll (mg m-3)
Inorganic Suspended Matter (mg L-1)
Organic Suspended Matter (mg L-1)
depth [m]

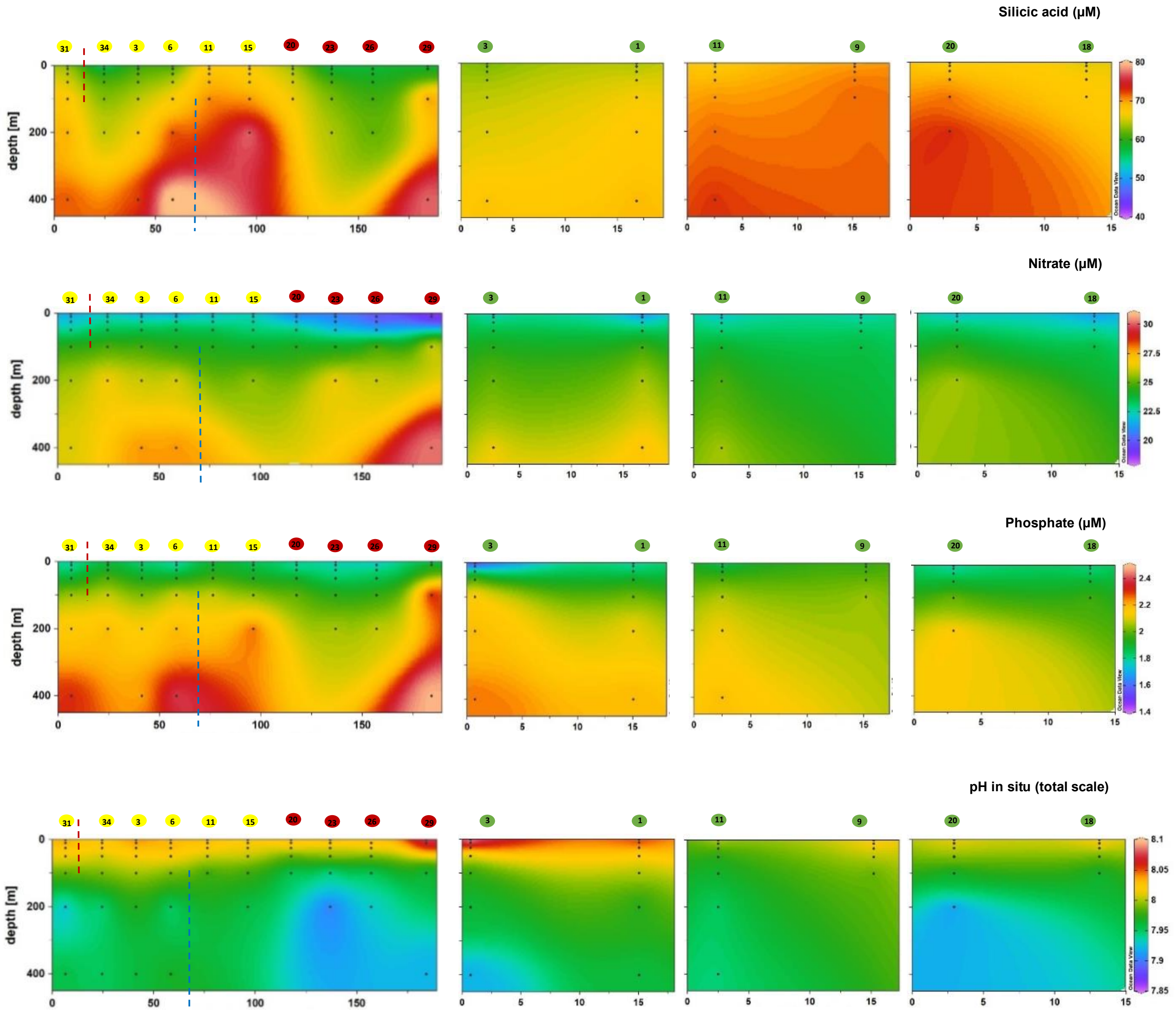

Supplementary Figure 1. Environmental variables considered in this study represented as vertical profiles. Each figure includes 13 vertical profiles (corresponding to 13 oceanographic stations), which are classified as Gerlache zone (yellow), fjords (green) and Bismarck zone (red). Each vertical profile includes 6 samples. Figures include the fronts: surface water thermal front (dashed red line) and the sub-pycnocline front (dashed blue line). The X axis represents the section distance (km). The sections represent (A) the transect including the central Gerlache and Bismarck stations, and the three different fjords (B: Wilhelmina Bay; C: Andvord Bay; D: Flandres Bay). Some stations (3, 11, 20) are duplicated to display differences between the inner and outer part of the fjords. The information regarding different stations is shown in Figure 2.

Environmental variables at 1 m depth

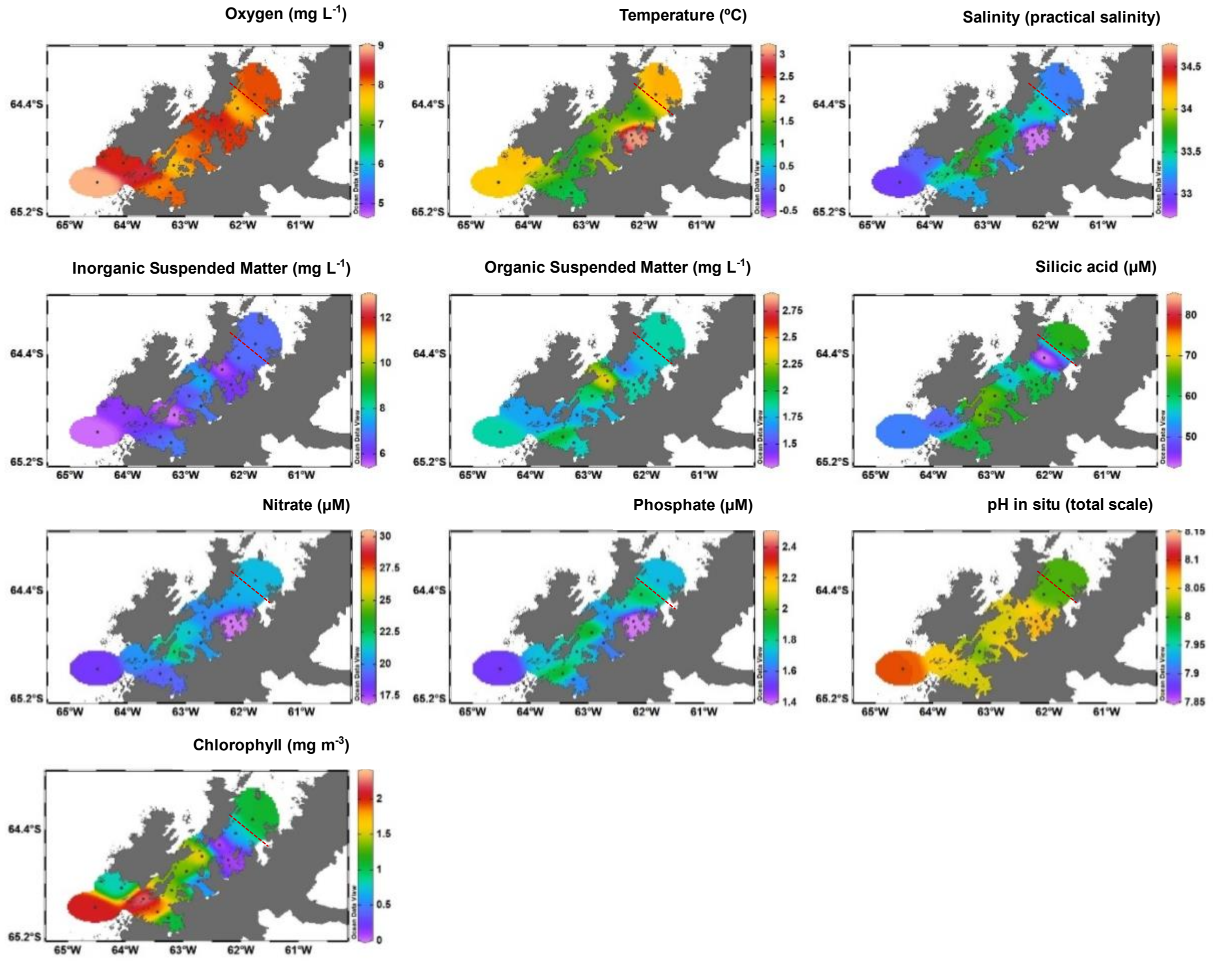

Supplementary Figure 2. Environmental variables at 1 m depth. Figures include the Surface water Thermal Front (dashed red line), situated from 1 to 100 m depth.

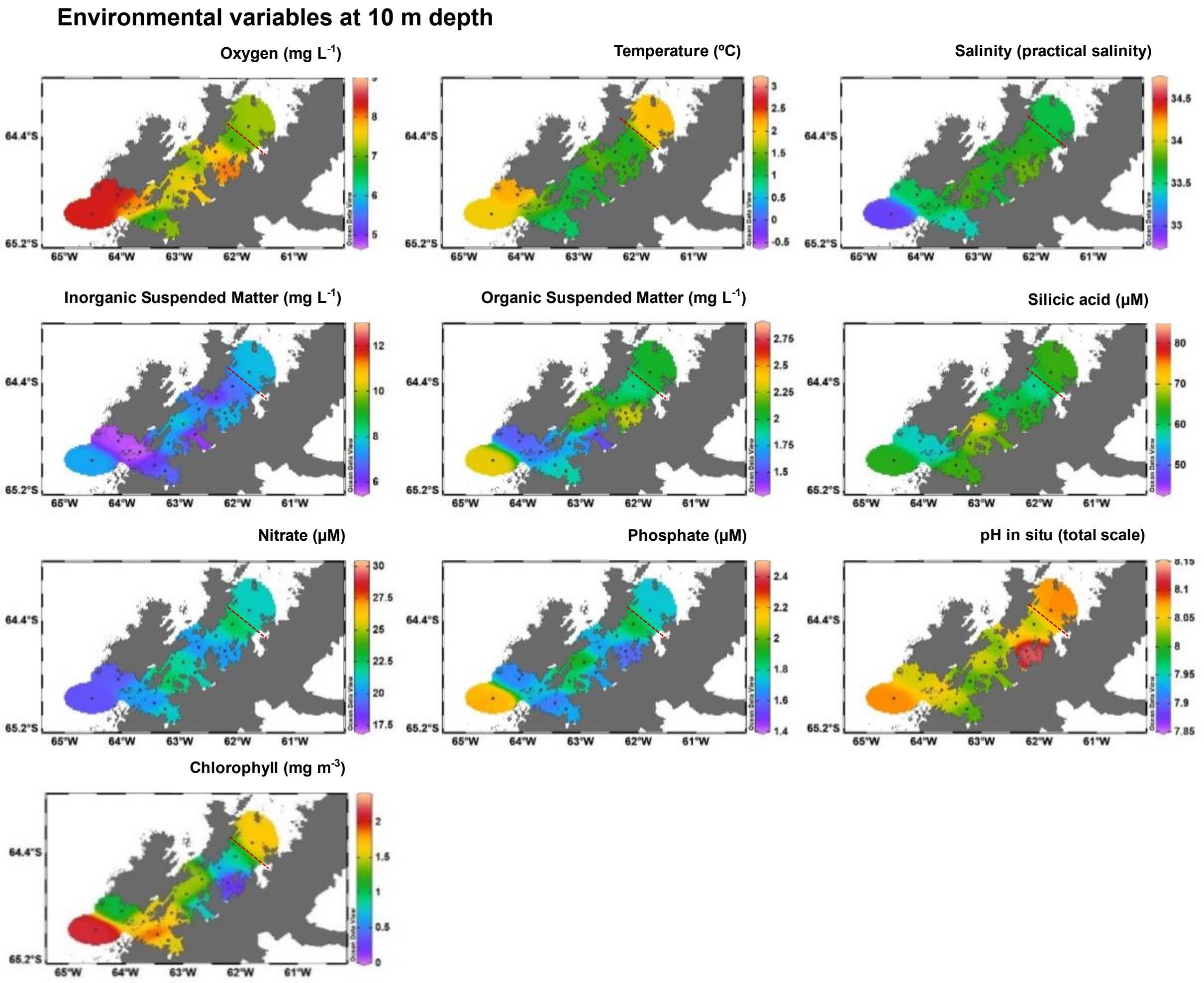

Supplementary Figure 3. Environmental variables at 10 m depth. Figures include the Surface water Thermal Front (dashed red line), situated from 1 to 100 m depth.

Environmental variables at 100 m depth

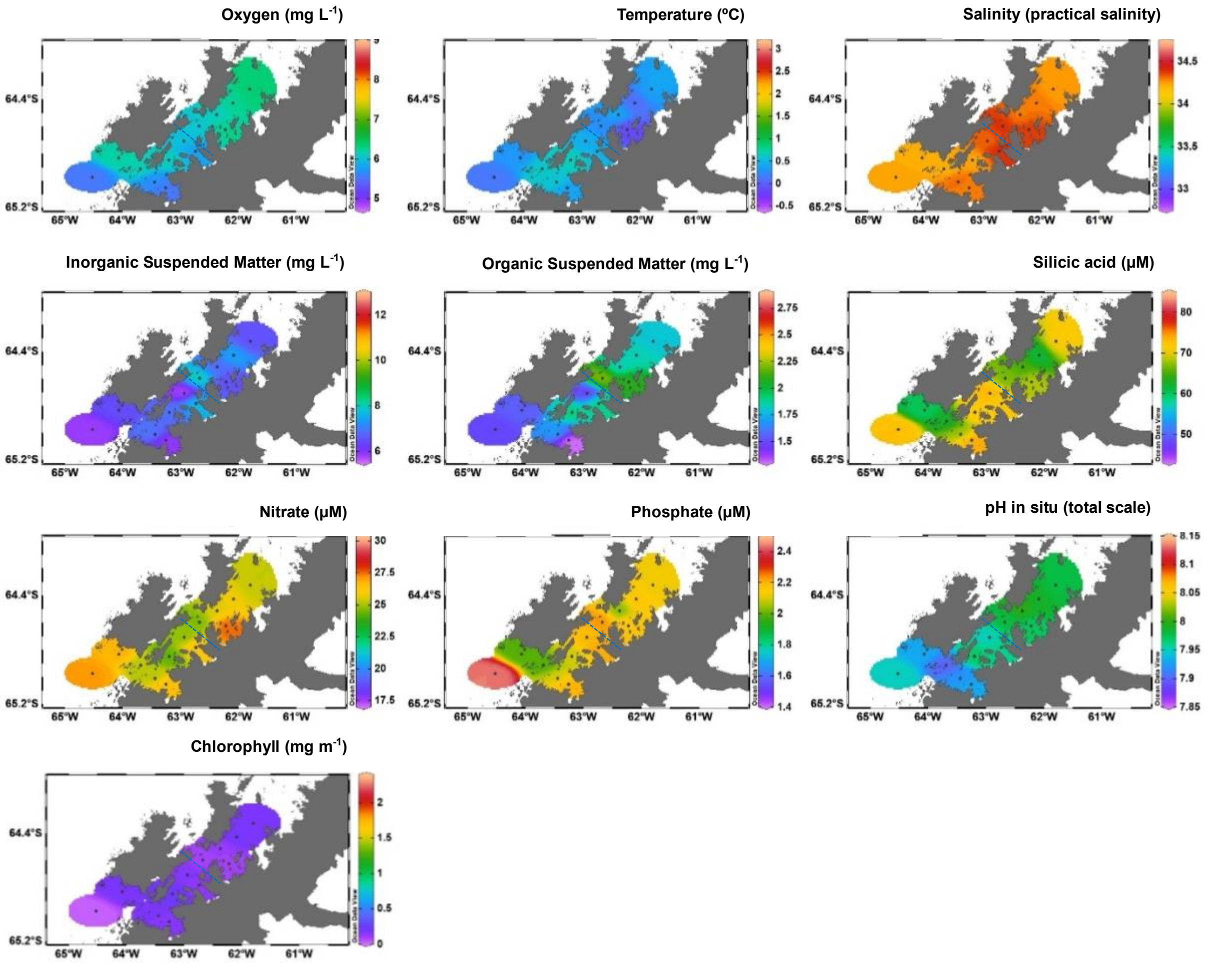

Supplementary Figure 4. Environmental variables at 100 m depth. Figures include the Sub-Pycnocline Front (the dashed blue line), situated below 100 m depth.

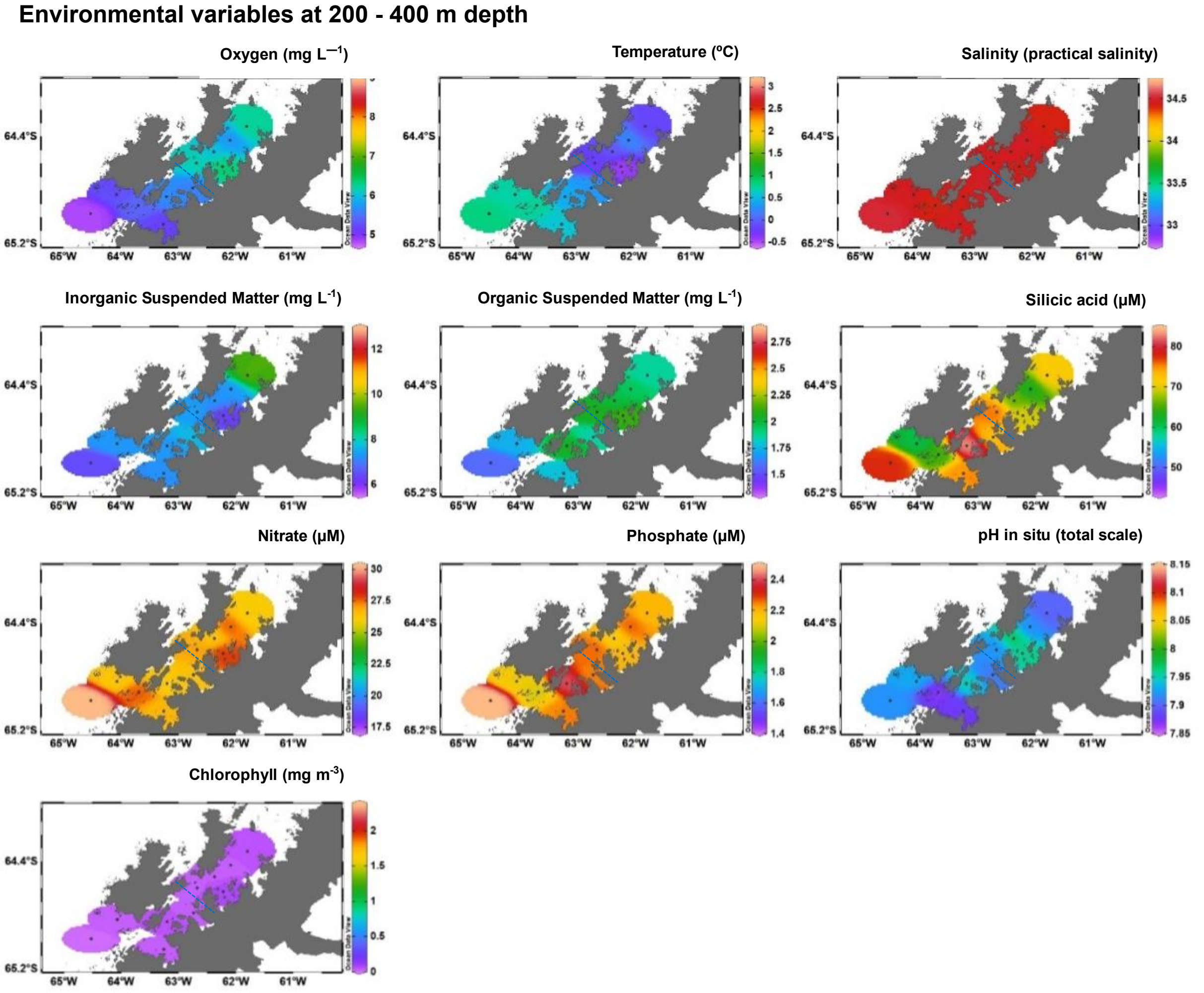

Supplementary Figure 5. Environmental variables at intermediate and deep depths. Figures include the sub-pycnocline front (the dashed blue line) situated below 100 m depth.

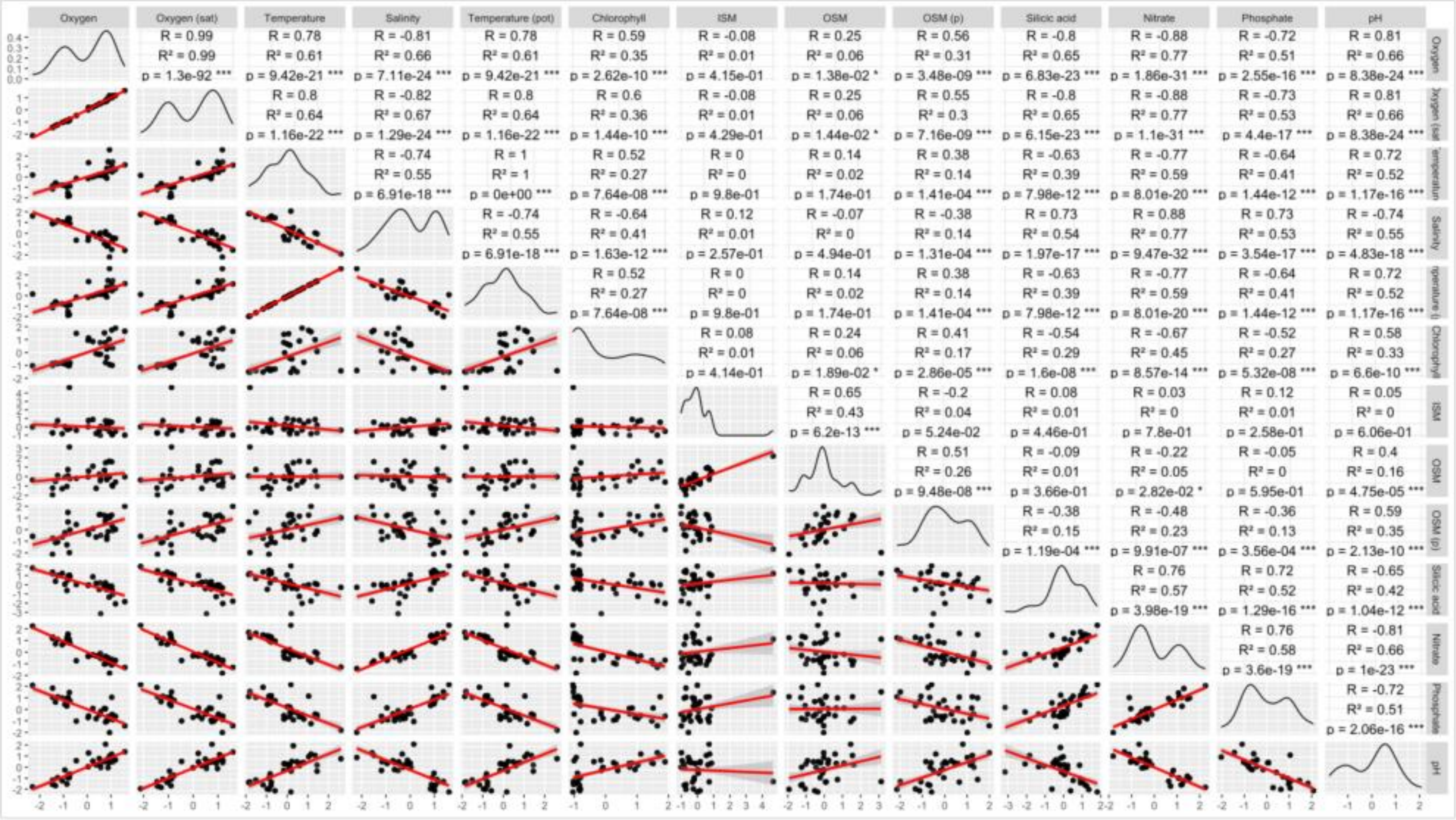

Supplementary Figure 6. Correlations (Spearman method) among the different environmental variables included in this study. Some of the environmental variables (partial pressure of CO_2, potential temperature and oxygen saturation percentage) correlated with other variables (with *in situ* pH, temperature and oxygen concentration, respectively) and were discarded for subsequent analysis (*bioenv* function).

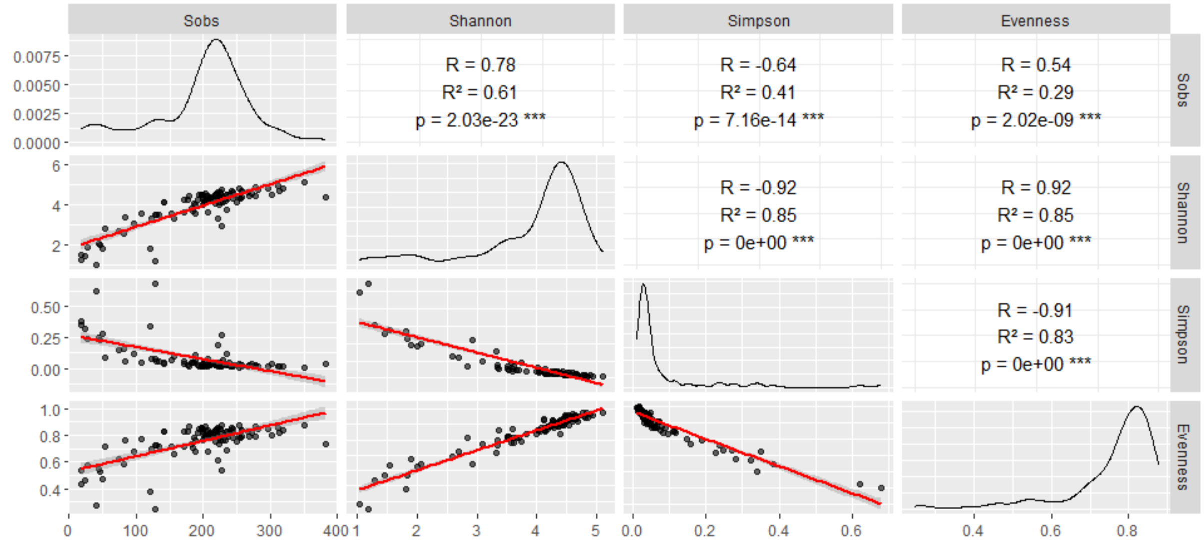

Supplementary Figure 7. Results of the correlations (Spearman method) conducted between the different alpha diversity indices. The upper right section displays the correlation value R (coefficient of correlation), R^2 (coefficient of determination), the p-values, and the significance of the p-values (thresholds considered in this study were 0.001***, 0.01**, and 0.05*).

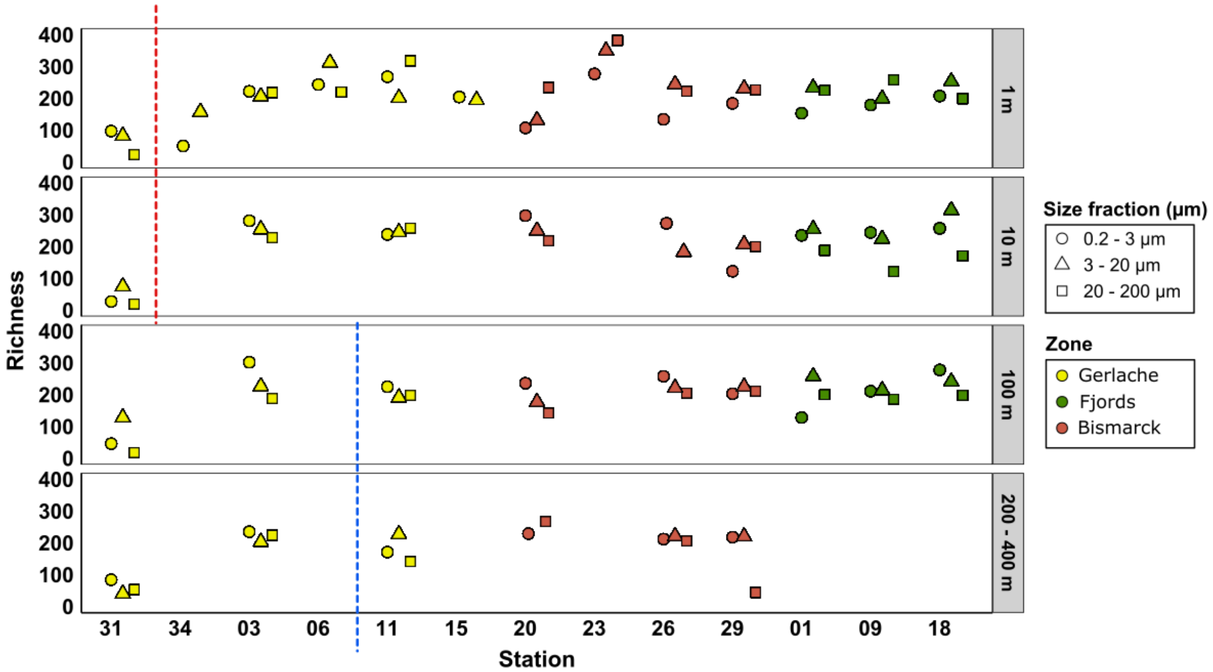

Supplementary Figure 8. Dotplot showing richness values (number of ASVs) along the Gerlache-Bismarck Strait, for each station, size fraction and depth. Stations are classified as Gerlache Strait zone (yellow), fjords (green) and Bismarck Strait zone (red). Figure includes the fronts: surface water thermal front (dashed red line) and the sub-pycnocline front (dashed blue line). A Kruskal-Wallis test revealed differences among size fractions (p-value < 0.001***), stations (p-value < 0.001***) and depths (p-value < 0.001***). Samples from stations 34, 15, 20, and 26 were excluded due to insufficient sequencing depth.

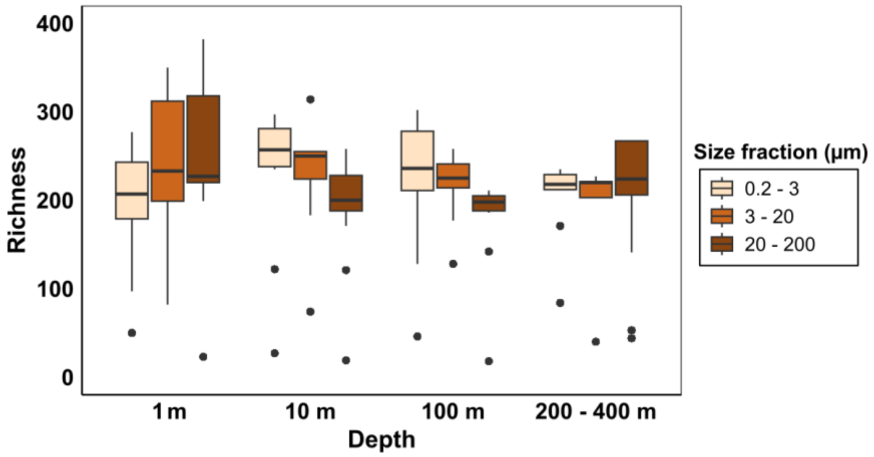

Supplementary Figure 9. Boxplot showing richness values (number of ASVs) for each size fraction and depth. The Kruskal-Wallis test revealed that richness was significantly different among size fractions and depths (p-value < 0.001).

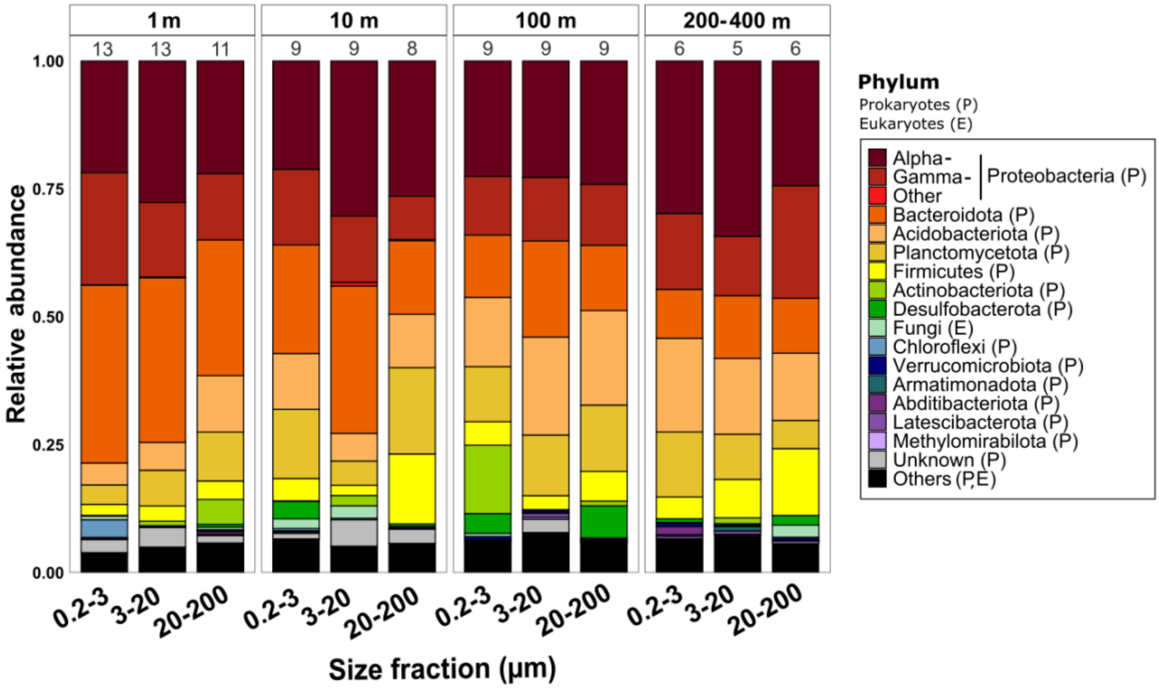

Supplementary Figure 10. Bar plot depicting the microbial community composition (represented at the Phylum level) of the Gerache-Bismarck Strait, at each depth and size-fraction. This plot includes samples from the channel, offshore and fjords. Only the 15 most abundant taxonomic groups (Prokaryotes and Eukaryotes) at the Phylum level are represented. The remaining groups were grouped together as 'Others,' along with those with an abundance of less than 1% per sample. The X-axis represents the size fractions and the Y-axis shows the relative abundance (%) grouped by depth. The number above the bars represent the N number (number of samples included in each bar).

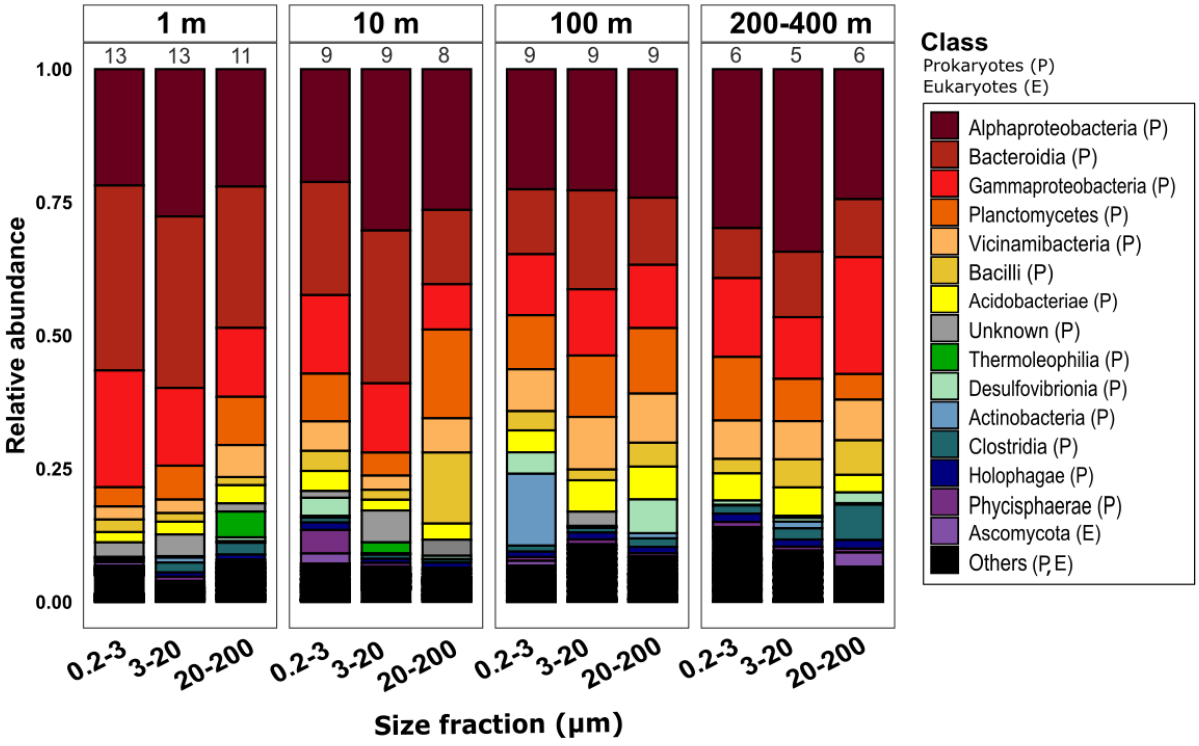

Supplementary Figure 11. Bar plot depicting the microbial community composition (represented at the Class level) of the Gerache-Bismarck Strait, at each depth, and size fraction. This plot includes samples from the Gerlache Strait zone (yellow), Bismarck Strait zone (red) and fjords (green). Only the 15 most abundant taxonomic groups (Prokaryotes and Eukaryotes) at the Class level are represented. The remaining groups were grouped together as 'Others,' along with those with an abundance of less than 1% per sample. The X-axis represents the size fractions and the Y-axis shows the relative abundance (%) grouped by depth.

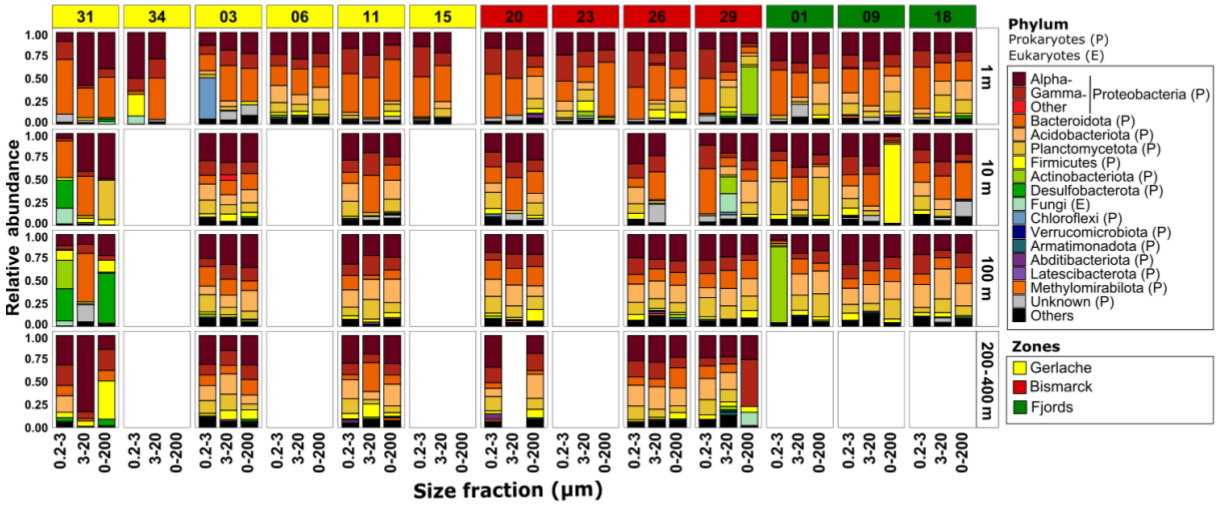

Supplementary Figure 12. Bar plot showing the microbial community composition (represented at the Phylum level) of the Gerache-Bismarck Strait, at each station, depth, and size-fraction. This plot includes samples from the Gerlache Strait zone (yellow), Bismarck Strait zone (red) and fjords (green). Only the 15 most abundant taxonomic groups (Prokaryotes and Eukaryotes) at the Phylum level are represented. The remaining groups were grouped together as 'Others,' along with those with an abundance of less than 1% per sample. The X-axis represents the size fractions and the Y-axis shows the relative abundance (%) grouped by depth. Samples from stations 34, 15, 20, and 26 were excluded due to low sequencing depth.

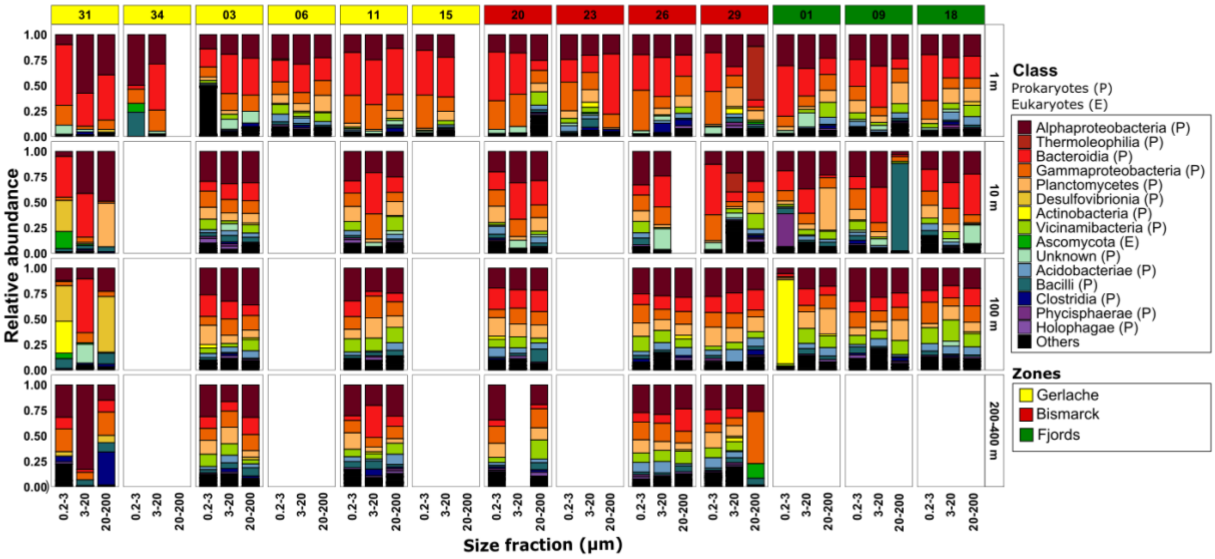

Supplementary Figure 13. Bar plot showing the microbial community composition (represented at the Class taxonomic level) of the Gerache-Bismarck Strait, at each station, depth, and size fraction. This plot includes samples from the Gerlache Strait zone (yellow), Bismarck Strait zone (red) and fjords (green). Only the 15 most abundant taxonomic groups (Prokaryotes and Eukaryotes) at the Class level are represented. The remaining groups were grouped together as 'Others,' along with those with an abundance of less than 1% per sample. The X-axis represents the size fractions and the Y-axis shows the relative abundance (%) grouped by depth. Samples from stations 34, 15, 20, and 26 were excluded due to low sequencing depth.

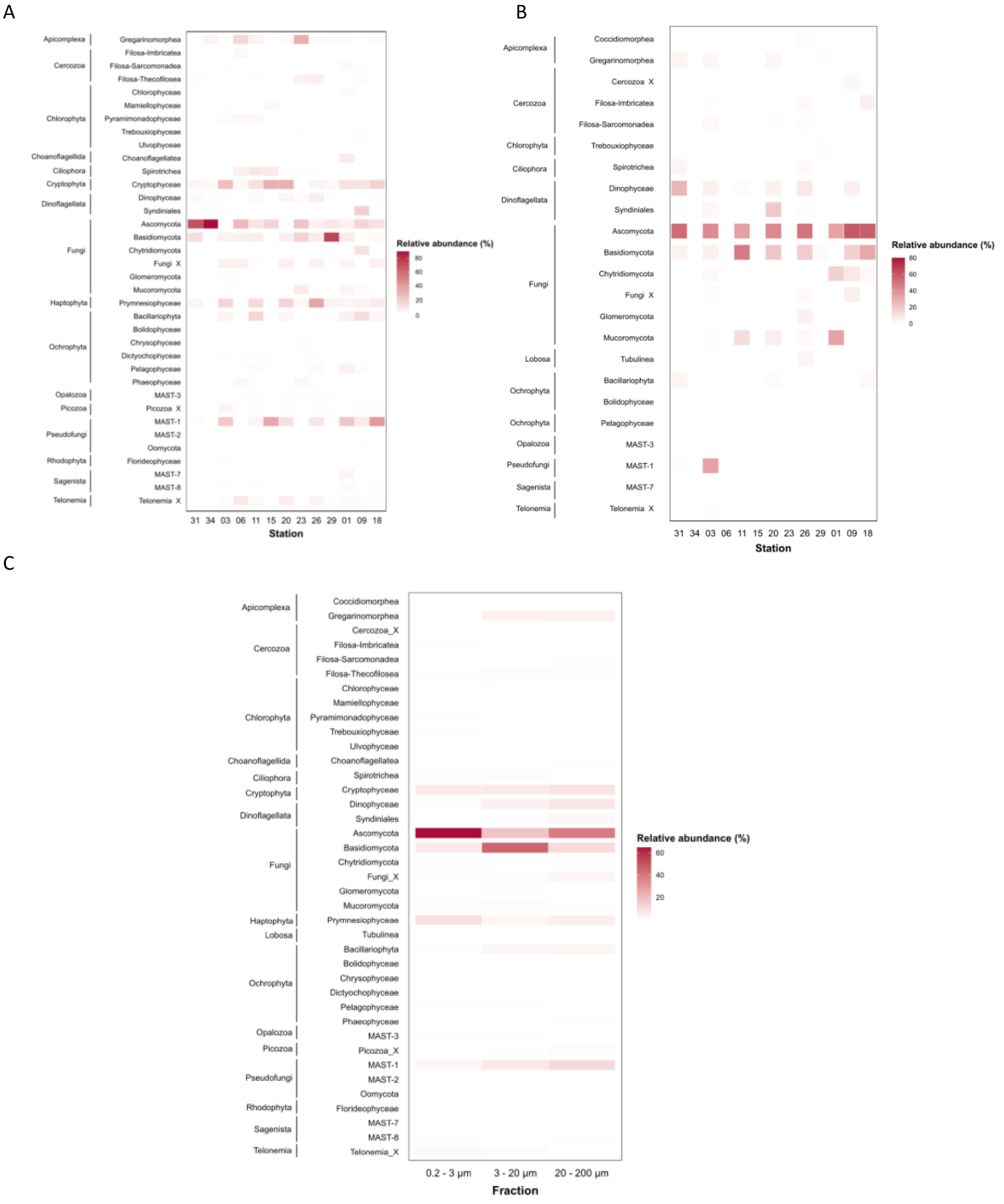

Supplementary Figure 14. Heatmaps revealing the relative abundance of Eukaryotes (at Phylum and Class level) at (A) surface waters (from 1 to 100 m), (B) at deep waters (from 200 to 400 m) and (C) at each size fraction.

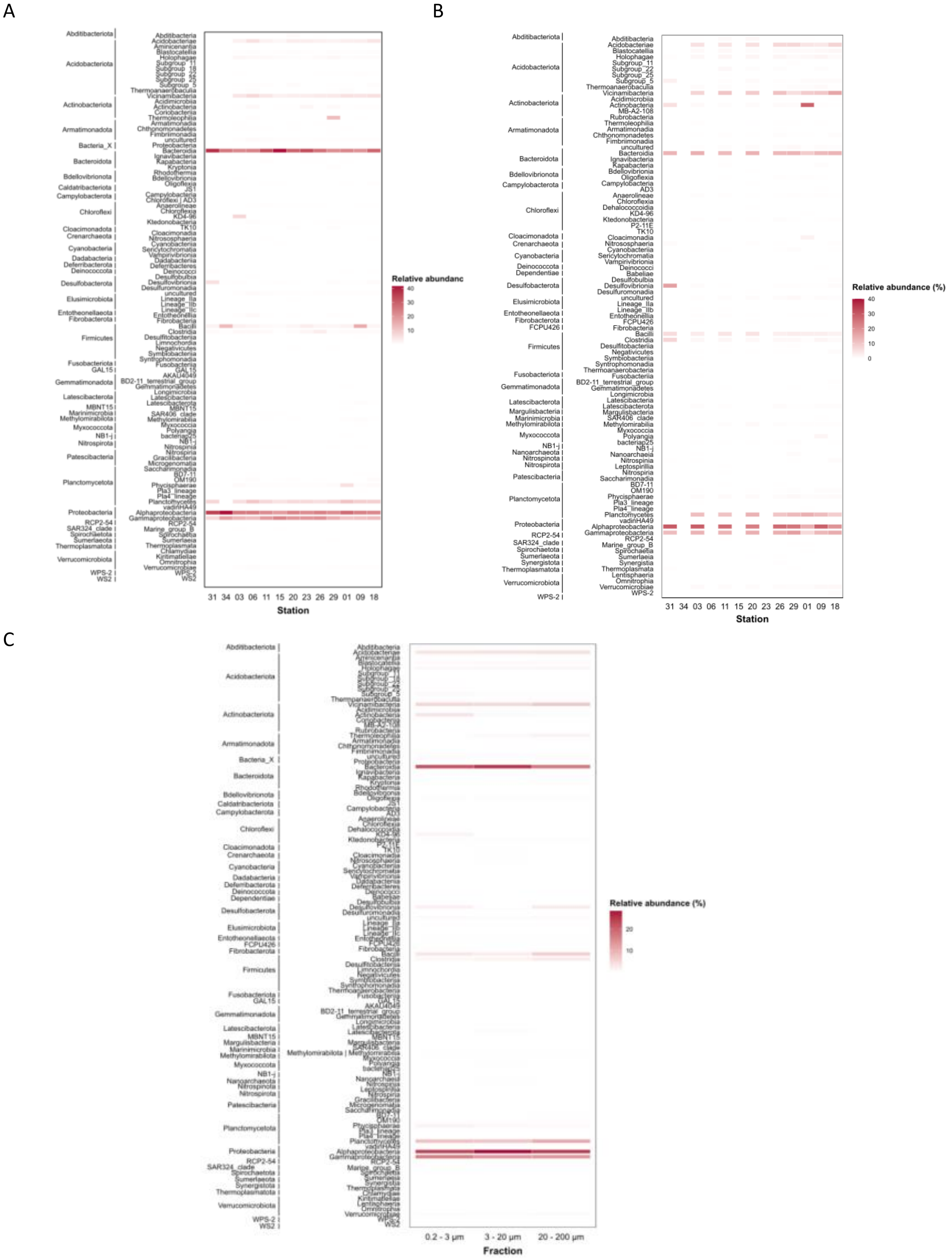

Supplementary Figure 15. Heatmap revealing the relative abundance of Prokaryotes (at Class and Order level) at (A) surface waters (from 1 to 100 m), (B) at deep waters (from 200 to 400 m) and **(C)** at each size fraction.

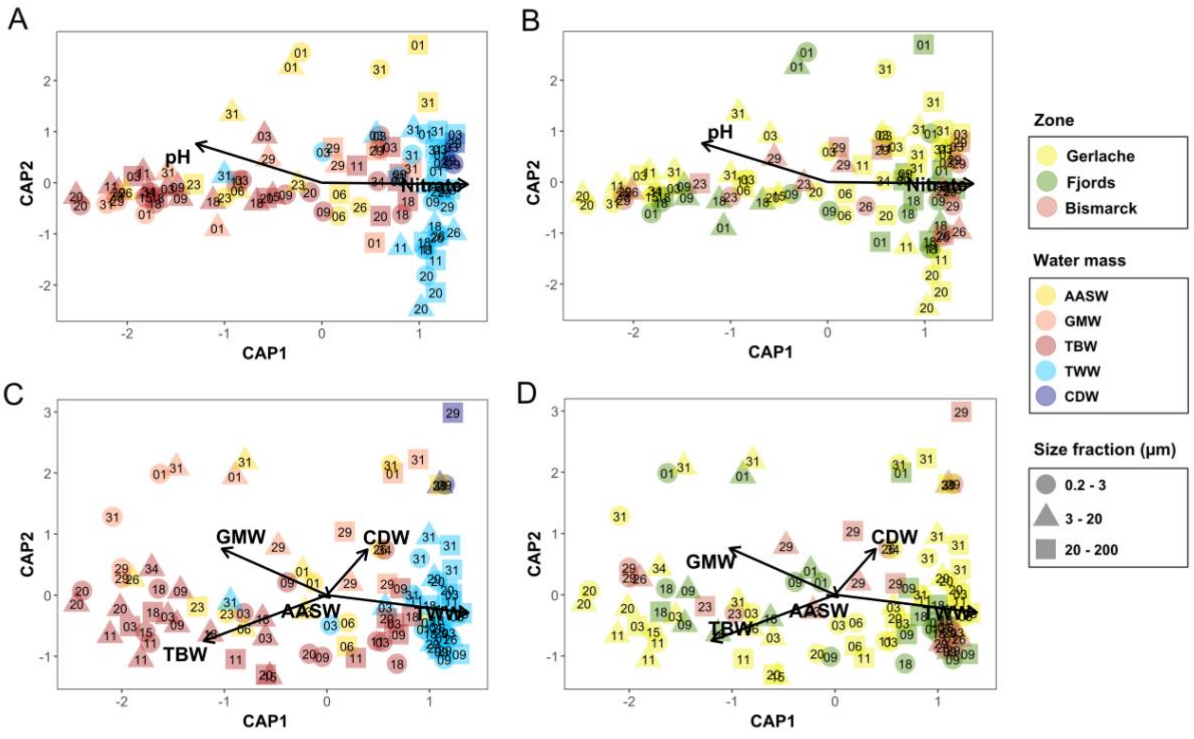

Supplementary Figure 16. Distance-based redundancy analysis (dbRDA) showing the influence of (A, B) biogeochemical variables and (C, D) water masses on microbial community composition. Ordinations are colored by water mass (A, C) and zone (B, D). Details regarding the dbRDA ordination and the selection of variables using the *bioenv* function procedure are included in the Materials and Methods section.

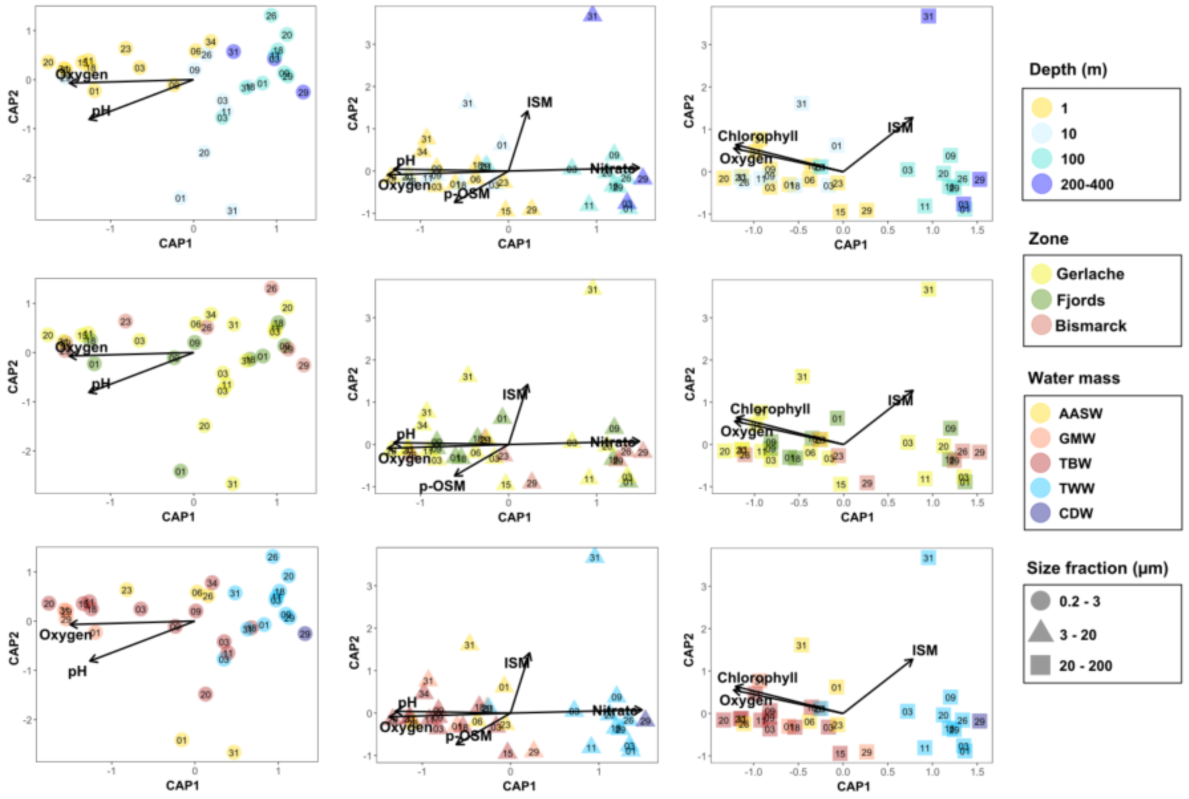

Supplementary Figure 17. Distance-based redundancy analysis (dbRDA) showing the influence of biogeochemical variables on microbial community composition, for each size-fraction. Samples are colored by depth, zone and water mass.

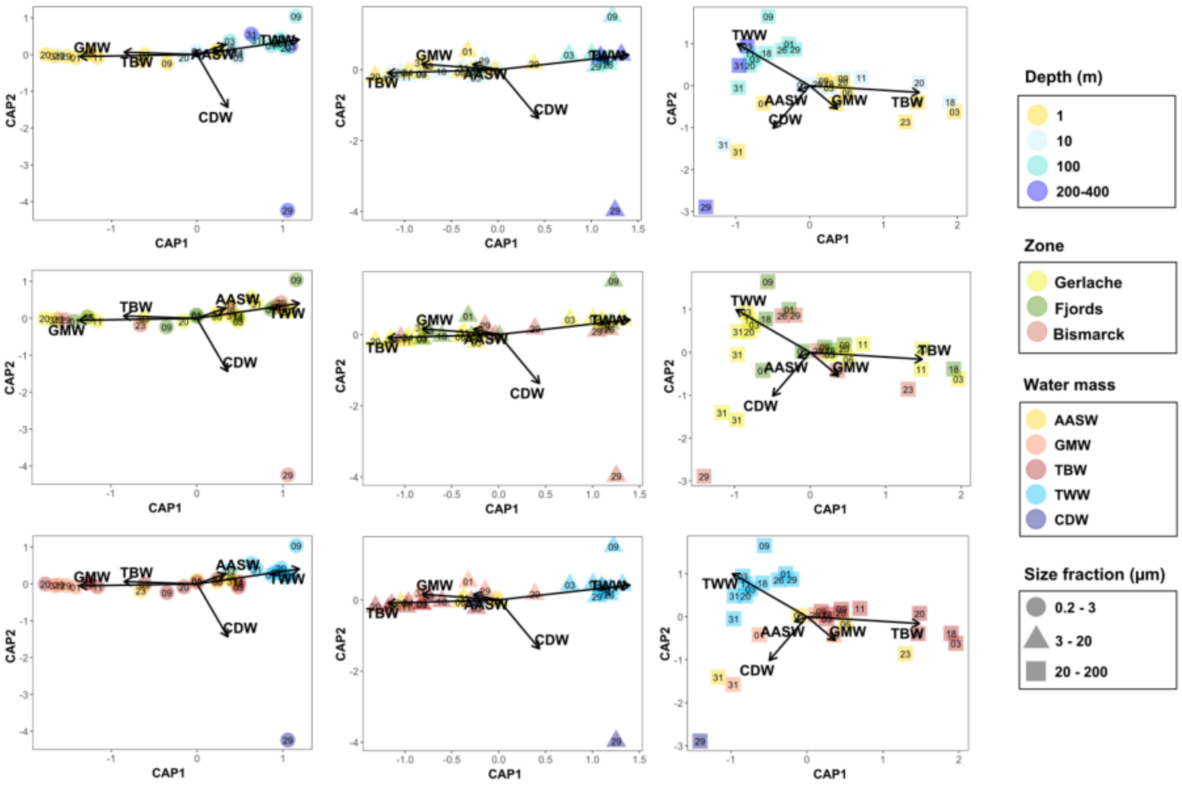

Supplementary Figure 18. Distance-based redundancy analysis (dbRDA) showing the influence of water masses on microbial community composition, for each size-fraction. Samples are colored by depth, zone and water mass.

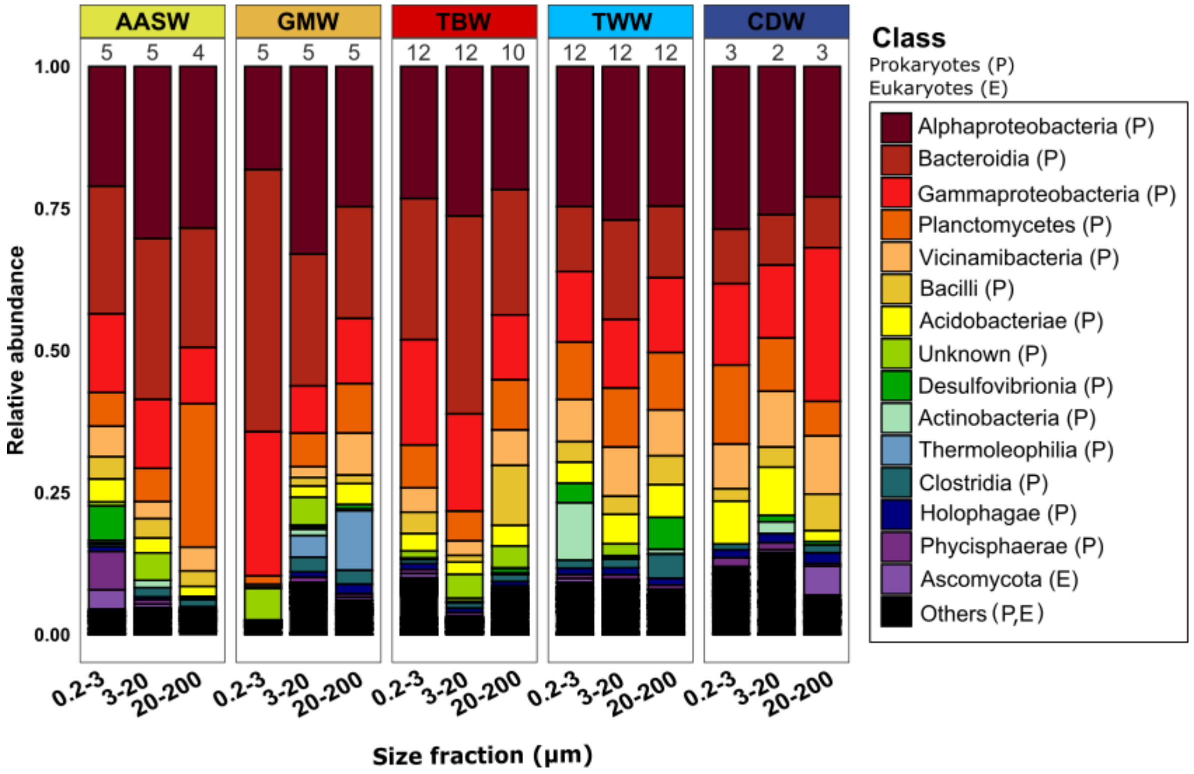

Supplementary Figure 19. Bar plot showing the microbial community composition (represented at the Class level) in each water mass, and size fraction. Only the 15 most abundant taxonomic groups (Prokaryotes and Eukaryotes) at the Class level are represented. The remaining groups were grouped together as 'Others,' along with those with an abundance of less than 1% per sample.

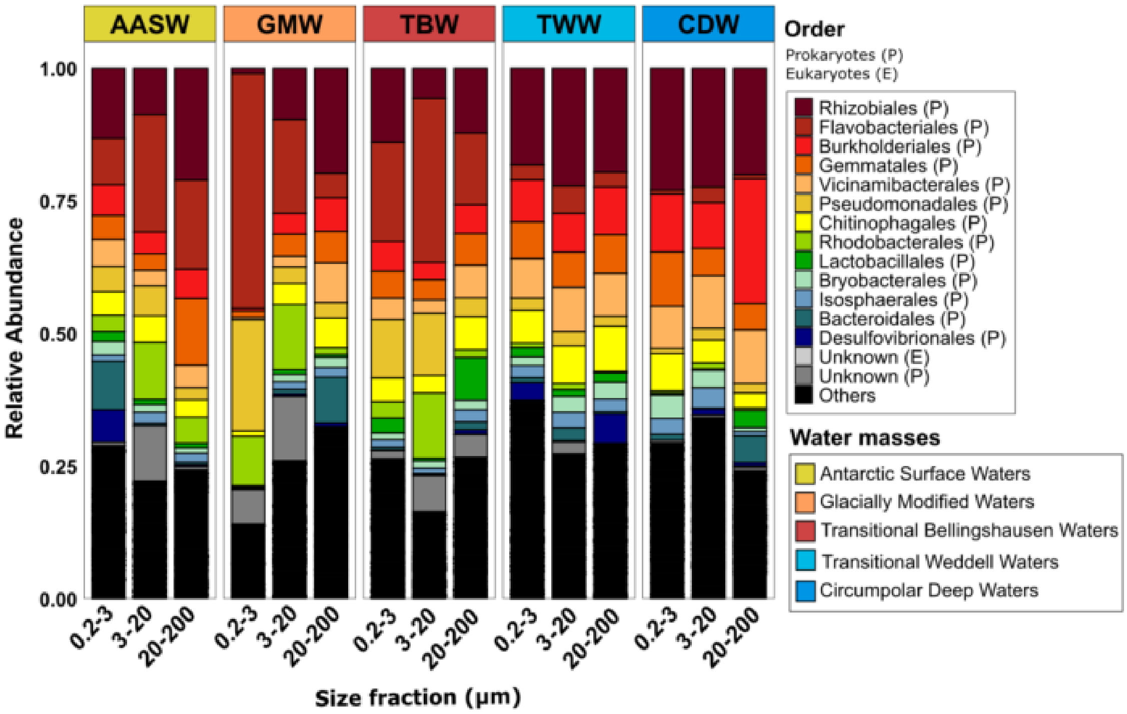

Supplementary Figure 20. Bar plot showing the microbial community composition (represented at the Order level) in each water mass, and size fraction. Only the 15 most abundant taxonomic groups (Prokaryotes and Eukaryotes) at the Order level are represented. The remaining groups were grouped together as 'Others,' along with those with an abundance of less than 1% per sample.

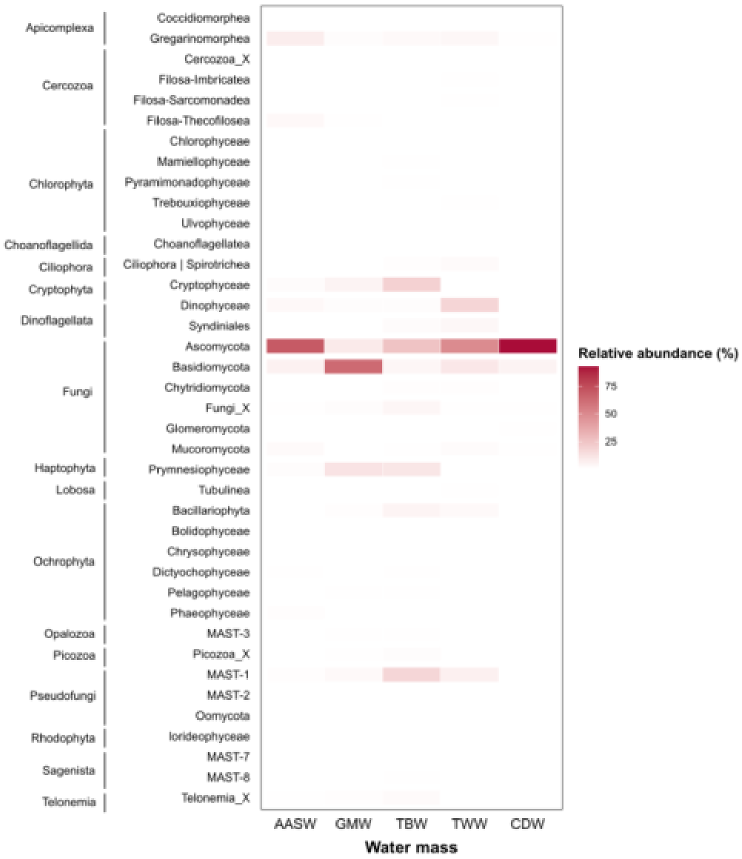

Supplementary Figure 21. Heatmap revealing the relative abundance of Eukaryotes (at Phylum and Class level) for each water mass.

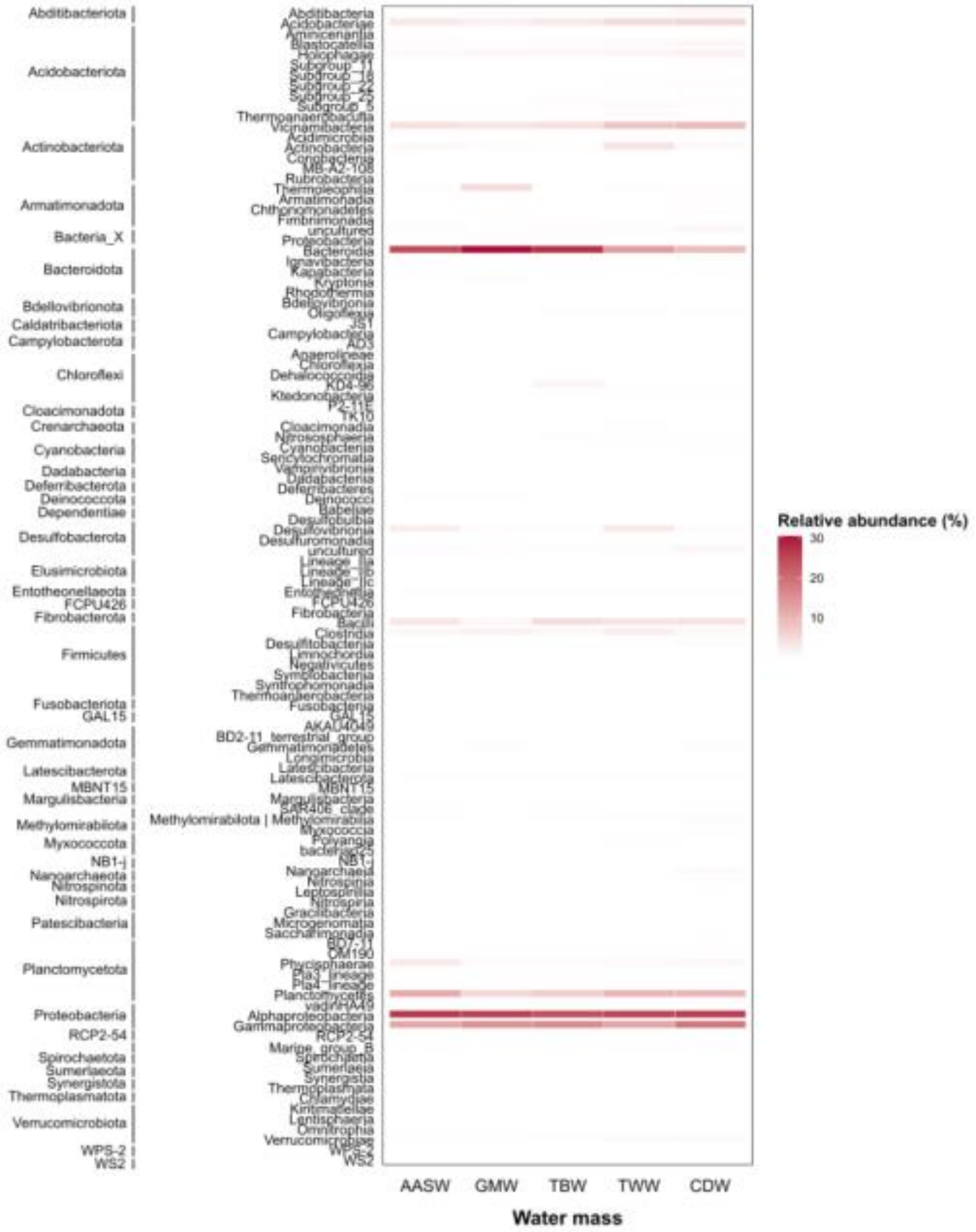

Supplementary Figure 22. Heatmap revealing the relative abundance of Prokaryotes (at Class and Order level) for each water mass.

Supplementary Table 4. List of indicator microorganisms for each water mass.

Water mass	ASV	Rel. Abundance (%)	Phylum, Class, Order, Family, Genus, Species
AASW	PROK_00996	0.3032741	Bacteroidota, Bacteroidia, Flavobacteriales, Flavobacteriaceae, NS4 marine group, Unknown
AASW	PROK_00089	1.138694	Proteobacteria, Alphaproteobacteria, Rhodobacterales, Rhodobacteraceae, *Ascidiaceihabitans*, Unknown
AASW	EUK_0021	0.280528	Cercozoa, Filosa-Thecofilosea, Cryomonadida, Cryothecomonas-lineage, *Cryothecomonas*, *Cryothecomonas aestivalis*
AASW	PROK_09764	0.08661222	Planctomycetota, Planctomycetes, Isosphaerales, Isosphaeraceae, Uncultured, Unknown
AASW	PROK_02031	0.2228458	Proteobacteria, Alphaproteobacteria, Rhizobiales, Xanthobacteraceae, *Bradyrhizobium*, Unknown
AASW	PROK_08135	0.06187353	Firmicutes, Clostridia, Oscillospirales, Oscillospiraceae, *Colidextribacter*, Unknown
AASW	PROK_09207	0.06191184	Proteobacteria, Alphaproteobacteria, Tistrellales, Geminicoccaceae, Candidatus Alysiosphaera, Unknown
AASW	PROK_08149	0.07430341	Acidobacteriota, Acidobacteriae, Bryobacterales, Bryobacteraceae, *Bryobacter*, Unknown
AASW	PROK_06091	0.0989854	Firmicutes, Clostridia, Lachnospirales, Lachnospiraceae, Oribacterium, Uncultured organism
AASW	EUK_0048	1.138614	Fungi, Ascomycota, Pezizomycotina, Eurotiomycetes, Aspergillus, Unknown
AASW	PROK_03214	0.09907121	Bacteroidota, Bacteroidia, Chitinophagales, Uncultured, Uncultured, bacterium episymbiont
AASW	PROK_01171	0.1815781	Proteobacteria, Alphaproteobacteria, Rhodobacterales, Rhodobacteraceae, *Octadecabacter*, Uncultured bacterium
AASW	PROK_03613	0.1155402	Proteobacteria, Alphaproteobacteria, Rhizobiales, Xanthobacteraceae, *Rhodoplanes*, metagenome
AASW	PROK_02019	0.03712412	Fusobacteriota, Fusobacteriia, Fusobacteriales, Fusobacteriaceae, *Fusobacterium*, Unknown
AASW	PROK_04801	0.1855976	Acidobacteriota, Acidobacteriae, Acidobacteriales, Uncultured, Uncultured, Unknown
AASW	PROK_00518	0.4083653	Bacteroidota, Bacteroidia, Flavobacteriales, Flavobacteriaceae, *Polaribacter*, Unknown
AASW	PROK_03718	0.5325077	Bacteroidota, Bacteroidia, Chitinophagales, Chitinophagaceae, *Flaviaesturariibacter*, Uncultured bacterium
AASW	PROK_07543	0.1238237	Proteobacteria, Gammaproteobacteria, Burkholderiales, Burkholderiaceae, *Lautropia*, Uncultured bacterium
AASW	PROK_02729	0.3838534	Armatimonadota, Uncultured, Uncultured, Uncultured, Uncultured, Unknown
AASW	PROK_04507	0.08662294	Bacteroidota, Bacteroidia, Chitinophagales, Saprospiraceae, Uncultured, Uncultured marine
AASW	PROK_06672	0.08661222	Proteobacteria, Alphaproteobacteria, Rhizobiales, Rhizobiales Incertae Sedis, *Nordella*, Uncultured bacterium

AASW	PROK_07515	0.08667657	Rhodobacterales, Proteobacteria, Alphaproteobacteria, Rhizobiales, Rhodobacteraceae, *Yoonia-Loktanella*, Uncultured bacterium
AASW	PROK_04170	0.3219814	Planctomycetota, Planctomycetes, Gemmatales, Gemmataceae, Gemmata, Uncultured bacterium
AASW	PROK_03363	0.0495356	Verrucomicrobiota, Verrucomicrobiae, Verrucomicrobiales, Rubritaleaceae, *Rubritalea*, Uncultured bacterium
AASW	PROK_05647	0.06186587	Proteobacteria, Alphaproteobacteria, Elsterales, Uncultured, Uncultured, Uncultured bacterium
AASW	PROK_02139	0.06187353	Bacteroidota, Bacteroidia, Flavobacteriales, NS9 marine group, NS9 marine group, Uncultured Flavobacterium
AASW	PROK_01053	0.978207	Bacteroidota, Bacteroidia, Chitinophagales, Chitinophagaceae, Uncultured, Unknown
CDW	PROK_05932	0.04950904	Proteobacteria, Alphaproteobacteria, Sphingomonadales, Sphingomonadaceae, *Sphingomonas*, Unknown
CDW	PROK_03369	0.1649893	Proteobacteria, Gammaproteobacteria, Burkholderiales, Oxalobacteraceae, *Massilia*, Unknown
CDW	PROK_06577	0.1113724	Thermoplasmatota, Thermoplasmata, Marine Group II, Marine Group II, Marine Group II, unidentified archaeon
CDW	PROK_01387	0.08664439	Proteobacteria, Gammaproteobacteria, Enterobacterales, Erwiniaceae, Unknown, Unknown
CDW	PROK_04212	0.123793	Proteobacteria, Gammaproteobacteria, Burkholderiales, Oxalobacteraceae, *Massilia*, Unknown
CDW	PROK_09655	0.03712871	Planctomycetota, Planctomycetes, Isosphaerales, Isosphaeraceae, Uncultured, Uncultured bacterium
CDW	PROK_00551	3.069687	Planctomycetota, Planctomycetes, Gemmatales, Gemmataceae, Uncultured, Uncultured bacterium
CDW	PROK_00195	0.3217623	Firmicutes, Negativicutes, Veillonellales-Selenomonadales, Veillonellaceae, *Veillonella*, Unknown
CDW	PROK_03028	0.3835684	Planctomycetota, Planctomycetes, Isosphaerales, Isosphaeraceae, Unknown, Unknown
CDW	PROK_01874	0.3835684	Acidobacteriota, Blastocatellia, 11-24, 11-24, 11-24, Uncultured Acidobacteriales
CDW	PROK_01047	1.138473	Bacteroidota, Bacteroidia, Chitinophagales, Chitinophagaceae, Uncultured, Unknown
CDW	PROK_03266	0.2723446	Proteobacteria, Alphaproteobacteria, Rhizobiales, Beijerinckiaceae, Unknown, Unknown
CDW	PROK_01375	0.3547855	Proteobacteria, Gammaproteobacteria, Burkholderiales, Oxalobacteraceae, *Massilia*, Unknown
CDW	PROK_00647	1.83123	Bacteroidota, Bacteroidia, Chitinophagales, Chitinophagaceae, *Flavisolibacter*, Unknown
CDW	PROK_00554	1.535033	Acidobacteriota, Vicinamibacteria, Vicinamibacterales, Uncultured, Uncultured, Uncultured Acidobacteria
CDW	EUK_0032	0.2478622	Fungi, Ascomycota, Pezizomycotina, Eurotiomycetes, Unknown, Unknown
CDW	PROK_02085	0.6557783	Proteobacteria, Gammaproteobacteria, Gammaproteobacteria Incertae Sedis, Unknown Family, *Acidibacter*, Uncultured Steroidobact
CDW	PROK_02447	0.3341998	Firmicutes, Bacilli, Bacillales, Bacillaceae, Bacillus, Unknown

CDW	PROK_00598	0.9530883	Acidobacteriota, Vicinamibacteria, Vicinamibacterales, Vicinamibacteraceae, Vicinamibacteraceae, Unknown
CDW	PROK_00489	0.8086476	Acidobacteriota, Holophagae, Subgroup 7, Subgroup 7, Subgroup 7, Unknown
CDW	PROK_03425	0.1484965	Proteobacteria, Alphaproteobacteria, Rhizobiales, Xanthobacteraceae, Unknown, Unknown
CDW	PROK_08312	0.06188885	Planctomycetota, Phycisphaerae, Tepidisphaerales, WD2101 soil group, WD2101 soil group, Uncultured soil
CDW	PROK_01550	0.2804586	Acidobacteriota, Holophagae, Subgroup 7, Subgroup 7, Subgroup 7, Unknown
CDW	PROK_03935	0.1856665	Proteobacteria, Alphaproteobacteria, Rhizobiales, Beijerinckiaceae, Unknown, Unknown
CDW	PROK_06113	0.1237777	Proteobacteria, Alphaproteobacteria, Sphingomonadales, Sphingomonadaceae, *Sphingomonas*, Unknown
CDW	PROK_01786	0.494927	Planctomycetota, Planctomycetes, Isosphaerales, Isosphaeraceae, *Aquisphaera*, Unknown
CDW	EUK_0052	0.7065824	Fungi, Ascomycota, Saccharomycotina, Saccharomycetales, *Debaryomyces*, Unknown
CDW	PROK_02670	0.1979953	Proteobacteria, Alphaproteobacteria, Rhizobiales, Beijerinckiaceae, *Microvirga*, Unknown
CDW	PROK_01323	1.683168	Acidobacteriota, Vicinamibacteria, Vicinamibacterales, Vicinamibacteraceae, Vicinamibacteraceae, Unknown
CDW	PROK_00060	43.9315	Proteobacteria, Gammaproteobacteria, Burkholderiales, Oxalobacteraceae, *Massilia*, Unknown
CDW	PROK_02255	0.3960396	Proteobacteria, Gammaproteobacteria, Burkholderiales, Alcaligenaceae, *Alcaligenes*, Unknown
CDW	PROK_03375	0.3960396	Thermoplasmatota, Thermoplasmata, Marine Group II, Marine Group II, Marine Group II, unidentified archaeon
CDW	EUK_0126	0.3219416	Fungi, Ascomycota, Pezizomycotina, Eurotiomycetes, Unknown, Unknown
CDW	PROK_00786	3.217822	Armatimonadota, Uncultured, Uncultured, Uncultured, Uncultured, Uncultured Firmicutes
CDW	PROK_06615	0.2474635	Proteobacteria, Alphaproteobacteria, Rhizobiales, Beijerinckiaceae, Methylobacterium-Methylorubrum, Unknown
CDW	PROK_06835	0.1981179	Planctomycetota, Planctomycetes, Isosphaerales, Isosphaeraceae, Uncultured, Uncultured bacterium
CDW	PROK_03697	0.3219416	Proteobacteria, Gammaproteobacteria, Gammaproteobacteria Incertae Sedis, Unknown Family, Candidatus Berkiella, Unknown
CDW	PROK_03786	0.1485149	Proteobacteria, Alphaproteobacteria, Rhizobiales, Xanthobacteraceae, Uncultured, Unknown
CDW	PROK_07457	0.1484781	Proteobacteria, Alphaproteobacteria, Rhizobiales, Beijerinckiaceae, *Psychroglaciecola*, Unknown
CDW	PROK_06560	0.3711952	Proteobacteria, Alphaproteobacteria, Elsterales, Uncultured, Uncultured, Unknown
CDW	PROK_03167	0.8415842	Proteobacteria, Gammaproteobacteria, Burkholderiales, SC-I-84, SC-I-84, Uncultured beta
CDW	PROK_02467	0.6438831	Acidobacteriota, Thermoanaerobaculia, Thermoanaerobaculales, Thermoanaerobaculaceae, Subgroup 10, Acidobacteria bacterium

CDW	PROK_00790	2.377415	Acidobacteriota, Vicinamibacteria, Vicinamibacterales, Uncultured, Uncultured, Unknown
CDW	PROK_02328	0.9156149	Acidobacteriota, Acidobacteriae, Acidobacteriales, Uncultured, Uncultured, Uncultured Acidobacteriaceae
CDW	PROK_02396	0.1732244	Firmicutes, Bacilli, Lactobacillales, Lactobacillaceae, Levilactobacillus, Unknown
CDW	PROK_03041	0.3467063	Acidobacteriota, Acidobacteriae, Solibacterales, Solibacteraceae, Candidatus Solibacter, Unknown
CDW	PROK_05910	0.2969562	Proteobacteria, Gammaproteobacteria, Burkholderiales, Nitrosomonadaceae, *Nitrosospira*, Unknown
CDW	PROK_01527	1.287129	Methylomirabilota, Methylomirabilia, Rokubacteriales, Rokubacteriales, Rokubacteriales, Uncultured bacterium
CDW	PROK_04067	0.2228826	Proteobacteria, Alphaproteobacteria, Rhizobiales, Beijerinckiaceae, *Microvirga*, Unknown
CDW	PROK_05421	0.2722098	Crenarchaeota, Nitrososphaeria, Nitrosopumilales, Nitrosopumilaceae, Nitrosopumilaceae, Unknown
CDW	PROK_06482	0.2722098	Chloroflexi, Anaerolineae, Anaerolineales, Anaerolineaceae, UTCFX1, Unknown
CDW	PROK_03014	0.4210005	Planctomycetota, Planctomycetes, Gemmatales, Gemmataceae, Uncultured, Uncultured bacterium
CDW	PROK_07778	0.1732244	Proteobacteria, Alphaproteobacteria, Sphingomonadales, Sphingomonadaceae, *Sphingomonas*, Unknown
CDW	PROK_04965	0.2475248	Planctomycetota, Planctomycetes, Planctomycetales, Rubinisphaeraceae, SH-PL14, Unknown
CDW	PROK_00856	1.088839	Acidobacteriota, Acidobacteriae, Bryobacterales, Bryobacteraceae, Bryobacter, Unknown
CDW	PROK_07784	0.2227723	Methylomirabilota, Methylomirabilia, Rokubacteriales, Rokubacteriales, Rokubacteriales, Uncultured bacterium
CDW	PROK_01520	1.658005	Planctomycetota, Planctomycetes, Gemmatales, Gemmataceae, Gemmata, Uncultured bacterium
CDW	PROK_03211	0.569166	Proteobacteria, Gammaproteobacteria, Burkholderiales, SC-I-84, SC-I-84, Unknown
CDW	PROK_01980	0.6930693	Proteobacteria, Gammaproteobacteria, Pseudomonadales, Pseudomonadaceae, *Pseudomonas*, Unknown
CDW	PROK_01076	0.07423905	Firmicutes, Bacilli, Lactobacillales, Lactobacillaceae, Leuconostoc, Unknown
CDW	PROK_03212	0.3474808	Proteobacteria, Alphaproteobacteria, Sphingomonadales, Sphingomonadaceae, *Sphingomonas*, Unknown
CDW	PROK_00344	0.7446016	Proteobacteria, Gammaproteobacteria, Enterobacterales, Alteromonadaceae, *Alishewanella*, Unknown
CDW	PROK_07211	0.1237624	Proteobacteria, Alphaproteobacteria, Sphingomonadales, Sphingomonadaceae, *Sphingomonas*, Unknown
GMW	EUK_0010	0.2878188	Haptophyta, Prymnesiophyceae, Prymnesiales, Chrysochromulinaceae, *Chrysochromulina*, *Chrysochromulina simplex*
GMW	PROK_00122	0.5299768	Bacteroidota, Bacteroidia, Flavobacteriales, Crocinitomicaceae, Crocinitomix, Uncultured Crocinitomix
GMW	PROK_01668	0.07423905	Firmicutes, Clostridia, Oscillospirales, Ruminococcaceae, Faecalibacterium, Unknown

GMW	PROK_04471	0.5196733	Proteobacteria, Gammaproteobacteria, Beggiatoales, Beggiatoaceae, Uncultured, Uncultured bacterium
GMW	PROK_10101	0.06187353	Proteobacteria, Alphaproteobacteria, Rhizobiales, Xanthobacteraceae, Uncultured, Uncultured forest
GMW	PROK_11095	0.06187353	Bacteroidota, Bacteroidia, Cytophagales, Microscillaceae, Uncultured, Uncultured soil
GMW	PROK_07200	0.1113586	Proteobacteria, Alphaproteobacteria, Acetobacterales, Acetobacteraceae, Uncultured, Unknown
GMW	PROK_11193	0.0989854	Verrucomicrobiota, Verrucomicrobiae, Chthoniobacterales, Chthoniobacteraceae, Candidatus Udaeobacter, Unknown
GMW	PROK_00694	0.3340757	Firmicutes, Clostridia, Peptostreptococcales-Tissierellales, Peptostreptococcaceae, *Peptostreptococcus*, Uncultured bacterium
GMW	PROK_08860	0.07425743	Actinobacteriota, Actinobacteria, Corynebacteriales, Mycobacteriaceae, *Mycobacterium*, Unknown
GMW	PROK_02165	0.7671368	Acidobacteriota, Vicinamibacteria, Vicinamibacterales, Uncultured, Uncultured, Unknown
GMW	PROK_03897	0.210396	Acidobacteriota, Blastocatellia, 11-24, 11-24, 11-24, Uncultured Acidobacteria
TBW	PROK_00456	0.286005	Bacteroidota, Bacteroidia, Flavobacteriales, Cryomorphaceae, Uncultured, Uncultured bacterium
TBW	PROK_00103	0.4795891	Bacteroidota, Bacteroidia, Flavobacteriales, NS9 marine group, NS9 marine group, Uncultured Flavobacteriia
TBW	PROK_00110	1.078588	Bacteroidota, Bacteroidia, Flavobacteriales, Cryomorphaceae, NS10 marine group, Uncultured Flavobacteriia
TBW	PROK_00085	1.463046	Bacteroidota, Bacteroidia, Flavobacteriales, NS7 marine group, NS7 marine group, Uncultured bacterium
TBW	PROK_02457	0.06049274	Proteobacteria, Alphaproteobacteria, Parvibaculales, OCS116 clade, OCS116 clade, Unknown
TBW	PROK_00062	0.903456	Bacteroidota, Bacteroidia, Flavobacteriales, Flavobacteriaceae, NS2b marine group, Unknown
TBW	PROK_00649	0.1287224	Proteobacteria, Gammaproteobacteria, Burkholderiales, Methylophilaceae, OM43 clade, Unknown
TBW	PROK_00316	0.6236183	Bacteroidota, Bacteroidia, Flavobacteriales, Cryomorphaceae, NS10 marine group, Uncultured Bacteroidetes/Chlorobi
TBW	PROK_03146	0.08248783	Unknown, Unknown, Unknown, Unknown, Unknown, Unknown
TBW	PROK_00036	0.938887	Proteobacteria, Gammaproteobacteria, Pseudomonadales, Thioglobaceae, SUP05 cluster, Uncultured marine
TBW	PROK_06319	0.05939124	Proteobacteria, Alphaproteobacteria, Rhodobacterales, Rhodobacteraceae, Uncultured, Unknown
TBW	PROK_00232	0.2009114	Proteobacteria, Gammaproteobacteria, Pseudomonadales, Pseudohongiellaceae, *Pseudohongiella*, Unknown
TBW	EUK_0014	0.3972969	Cryptophyta, Cryptophyceae, Cryptomonadales, Cryptomonadales X, *Geminigera*, *Geminigera cryophila*
TBW	PROK_01050	0.115483	Marinimicrobia (SAR406 clade), Marinimicrobia (SAR406 clade), Marinimicrobia (SAR406 clade), Marinimicrobia (SAR406 clad Marinimicrobia (SAR406 clade), Uncultured marine
TWW	PROK_01014	0.4269142	Proteobacteria, Gammaproteobacteria, Burkholderiales, Nitrosomonadaceae, Ellin6067, Unknown

TWW	PROK_02182	0.1773488	Proteobacteria, Gammaproteobacteria, Burkholderiales, Oxalobacteraceae, *Massilia*, *Duganella* sp.
TWW	PROK_01064	0.1273345	Proteobacteria, Gammaproteobacteria, Xanthomonadales, Rhodanobacteraceae, *Rhodanobacter*, *Rhodanobacter* sp.

Supplementary Table 5. List of the core microbes of each water mass.

Water Mass	ASVs	Rel. Abundance (%)	Phylum, Class, Order, Family, Genus, Species
CDW	PROK_00001	17.01	Proteobacteria, Alphaproteobacteria, Rhizobiales, Beijerinckiaceae, *Methylobacterium-Methylorubrum*, Unknown
CDW	PROK_00011	0.92	Firmicutes, Bacilli, Staphylococcales, Staphylococcaceae, *Staphylococcus*, Unknown
CDW	PROK_00046	0.26	Proteobacteria, Gammaproteobacteria, Enterobacterales, Enterobacteriaceae, *Escherichia-Shigella*, Unknown
CDW	PROK_00094	1.24	Proteobacteria, Gammaproteobacteria, Burkholderiales, Oxalobacteraceae, *Massilia*, Unknown
CDW	PROK_00156	0.58	Proteobacteria, Gammaproteobacteria, Burkholderiales, Oxalobacteraceae, *Massilia*, Unknown
CDW	PROK_00173	0.41	Proteobacteria, Gammaproteobacteria, Burkholderiales, Oxalobacteraceae, *Massilia*, Unknown
TWW	PROK_00001	15.28	Proteobacteria, Alphaproteobacteria, Rhizobiales, Beijerinckiaceae, *Methylobacterium-Methylorubrum*, Unknown
TWW	PROK_00046	0.46	Proteobacteria, Gammaproteobacteria, Enterobacterales, Enterobacteriaceae, *Escherichia-Shigella*, Unknown
TWW	PROK_00094	1.03	Proteobacteria, Gammaproteobacteria, Burkholderiales, Oxalobacteraceae, *Massilia*, Unknown
TWW	PROK_00156	0.69	Proteobacteria, Gammaproteobacteria, Burkholderiales, Oxalobacteraceae, *Massilia*, Unknown
GMW	PROK_00001	8.31	Proteobacteria, Alphaproteobacteria, Rhizobiales, Beijerinckiaceae, *Methylobacterium-Methylorubrum*, Unknown
GMW	PROK_00003	5.15	Proteobacteria, Alphaproteobacteria, Rhodobacterales, Rhodobacteraceae, *Sulfitobacter*, Unknown
GMW	PROK_00020	0.97	Bacteroidota, Bacteroidia, Flavobacteriales, Flavobacteriaceae, *Ulvibacter*, Uncultured marine
GMW	PROK_00024	3.93	Bacteroidota, Bacteroidia, Flavobacteriales, Cryomorphaceae, Uncultured, marine metagenome
GMW	PROK_00032	2.93	Bacteroidota, Bacteroidia, Flavobacteriales, Flavobacteriaceae, *Polaribacter*, Unknown
GMW	PROK_00043	4.68	Bacteroidota, Bacteroidia, Flavobacteriales, Cryomorphaceae, *Vicingus*, Uncultured bacterium
AASW	PROK_00001	11.39	Proteobacteria, Alphaproteobacteria, Rhizobiales, Beijerinckiaceae, *Methylobacterium-Methylorubrum*, Unknown
AASW	PROK_00003	2.85	Proteobacteria, Alphaproteobacteria, Rhodobacterales, Rhodobacteraceae, *Sulfitobacter*, Unknown
AASW	PROK_00015	1.46	Proteobacteria, Gammaproteobacteria, Pseudomonadales, Nitrincolaceae, Uncultured, Uncultured marine
TBW	PROK_00001	8.06	Proteobacteria, Alphaproteobacteria, Rhizobiales, Beijerinckiaceae, *Methylobacterium-Methylorubrum*, Unknown
TBW	PROK_00003	2.91	Proteobacteria, Alphaproteobacteria, Rhodobacterales, Rhodobacteraceae, *Sulfitobacter*, Unknown
TBW	PROK_00015	3.86	Proteobacteria, Gammaproteobacteria, Pseudomonadales, Nitrincolaceae, Uncultured, Uncultured marine
TBW	PROK_00020	1.87	Bacteroidota, Bacteroidia, Flavobacteriales, Flavobacteriaceae, *Ulvibacter*, Uncultured marine

TBW	PROK_00024	2.99	Bacteroidota, Bacteroidia, Flavobacteriales, Cryomorphaceae, Uncultured, marine metagenome
TBW	PROK_00027	1.93	Proteobacteria, Pseudomonadales, Gammaproteobacteria, Nitrincolaceae, Uncultured, Uncultured marine
TBW	PROK_00032	1.39	Bacteroidota, Bacteroidia, Flavobacteriales, Flavobacteriaceae, *Polaribacter*, Unknown
TBW	PROK_00036	0.80	Proteobacteria, Gammaproteobacteria, Pseudomonadales, Thioglobaceae, SUP05 cluster, Uncultured marine
TBW	PROK_00045	1.00	Bacteroidota, Bacteroidia, Flavobacteriales, Flavobacteriaceae, NS5 marine group, Unknown
TBW	PROK_00059	0.75	Proteobacteria, Alphaproteobacteria, SAR11 clade, Clade I, Clade Ia, Unknown
TBW	PROK_00094	0.57	Proteobacteria, Gammaproteobacteria, Burkholderiales, Oxalobacteraceae, *Massilia*, Unknown

Supplementary Table 6. DNA sequences of the indicator microbes.

Water mass	ASV	DNA sequence
AASW	PROK_00996	TACGGAGGATCCAAGCGTTATCCGGAATCATTGGGTTTAAAGGGTCCGCAGGCGGTCTTTTAAGTCAGAGGTGAAAGCCTACAGCTCAACTGTAGAACGGCCTTTGAAACTGAAAGACTTGAGTTATTGTGAAGTGGTTAGAATGTGTGGTGTAGCGGTGAAATGCATAGATATCACACAGAATACCAATTGCGAAGGCAGATCACTAACAATATACTGACGCTCATGGACGAAAGCGTGGGGAGCGAACAGGATTAGATACCCTGGTAGTCCACGCCGTAAACGATGGATACTAGCTGTTCGTCTAACATTGGTTAGATGAGTGGCTAAGCGAAAGTGATAAGTATCCCACCTGGGGAGTACGGTCGCAAGACTG
AASW	PROK_00089	TACGGAGGGGGTTAGCGTTGTTCGGAATTACTGGGCGTAAAGCGCACGTAGGCGGATTAGAAAGTTAGAGGTGAAATCCCAGGGCTCAACCCTGGAACTGCCTTTAAAACTCCTAGTCTTGAGTTCGAGAGAGGTGAGTGGAATTCCAAGTGTAGAGGTGAAATTCGTAGATATTTGGAGGAACACCAGTGGCGAAGGCGGCTCACTGGCTCGATACTGACGCTGAGGTGCGAAAGTGTGGGGAGCAAACAGGATTAGATACCCTGGTAGTCCACACCGTAAACGATGAATGCCAGTCGTCGGGTAGTATACTATTCGGTGACACACCTAACGGATTAAGCATTCCGCCTGGGGAGTACGGTCGCAAGATTA
AASW	EUK_0021	TTCCAGCTCCAAGAGCGTATATTAAAGTTGTTGCAGTTAAAAAGCTCGTAGTTGGATTTCTGGTACAACGCGCCTGGCCCGCTTTTAGTGAGTGCCGGTGCGCGTTTGCCATCCTTCTAGAAAACGTTTCTACCTGGGCAACCGGGCCGGACTCGCGATCTAGATCTTTTACTTTGAAAAAATTAGAGTGTTTAAAGCAGGCTTTTGCTGTGAATACATTAAGGATGTCTTCTTTAATCAAGAACGAAAGTTGGGGGATCGAAGACGATCAGATACCGTCGTAGTCTCAACCATAAACTATGCCAACTAGGGATGGGTGAACGTTGTTTTTTTGACTTCATCTGCACCTTATGAGAAATCAAAGTCTTTGGGTTCCGGGGGGAGTATGGTCGCAAGGCTG
AASW	PROK_09764	GACGAACCGTGCGAACGTTATTCGGAATCACTGGGCTTAAAGCGCGTGTAGGCGGCCCGGCACGTCCGGCGCTGAAATCCCCCGGCTCAACCGGGGAAGGGGCGCGGATACGACCGGGCTGGAGGGGGGTAGGGGGAGCCGGAACTCACGGTGGAGCGGTGAAATGCGTTGAGATCGTGAGGAACGCCCGTGGCGAAAGCGGGCTCCTGGACCCCGCCTGACGCTGAGACGCGAAAGCCAGGGGAGCGAACGGGATTAGATACCCCGGTAGTCCTGGCCGTAAACCATGGGCACTGGGTAGGGGGCTCGCCGATGGGCTCCCTGCCGCAGCGAAAGCGTGAAGTGCCCCGCCTGGGGAGTATGGTCGCAAGGCTG
AASW	PROK_02031	TACGAAGGGGGCTAGCGTTGCTCGGAATCACTGGGCGTAAAGGGTGCGTAGGCGGGTCTTTAAGTCAGGGGTGAAATCCTGGAGCTCAACTCCAGAACTGCCTTTGATACTGAGGATCTTGAGTCCGGGAGAGGTGAGTGGAACTGCGAGTGTAGAGGTGAAATTCGTAGATATTCGCAAGAACACCAGTGGCGAAGGCGGCTCACTGGCCCGGTACTGACGCTGAGGCACGAAAGCGTGGGGAGCAAACAGGATTAGATACCCTGGTAGTCCACGCCGTAAACGATGAATGCCAGCCGTTAGTGGGTTTACTCACTAGTGGCGCAGCTAACGCTTTAAGCATTCCGCCTGGGGAGTACGGTCGCAAGATTA
AASW	PROK_08135	TACGTAGGTGGCAAGCGTTATCCGGATTTACTGGGTGTAAAGGGCGTGTAGGCGGGACTGCAAGTCAGGCGTGAAAACCAAGGGCTCAACCCTTGGCCTGCGTTTGAAACTGTAGTTCTTGAGTACTGGAGAGGCAGATGGAATTCCTAGTGTAGCGGTGAAATGCGTAGATATTAGGAGGAACACCAGTGGCGAAGGCGATCTGCTGGACAGCAACTGACGCTAAGGCGCGAAAGCGTGGGGAGCAAACAGGATTAGATACCCTGGTAGTCCACGCTGTAAACGATGGATACTAGGTGTGGGGGGTCTGACCCCCTCCGTGCCGCAGCTAACGCAATAAGTATCCCACCTGGGGAGTACGATCGCAAGGTTG
AASW	PROK_09207	GACGGAGGGTGCTAGCGTTGTTCGGAATCACTGGGCGTAAAGGGCGCGTAGGCGGCCGCCCGCGTCGGACGTGAAAGCCCGGGGCTCAACCCCGGAACGGCGTCCGATACGGGGCGGCTCGAGGCCGGGAGAGGGGGGTGGAATACCCAGTGTAGAGGTGAAATTCGTAGATATTGGGTGGAACACCGGTGGCGAAAGCGGCCCCCTGGCCCGGTCCTGACGCTGAGGCGCGAAAGCGTGGGGAGCAAACAGGATTAGATACCCTGGTAGTCCACGCCCTAAACGATGTGGGCTGGGCGTCGGGGGGCTCGCCCCCCGGTGCCGGAGCTAACGCGGTAAGCCCACCGCCTGGGGAGTACGGCCGCAAGGTTA
AASW	PROK_08149	TACGTAGGCAGCGAGCGTTGTTCGGAGTTACTGGGCGTAAAGCGTGCGTAGGCGGTGGCCTAAGTCTGTTGTGAAATCTCCCGGCTTAACCGGGAGGGTGCGGCGGAAACTGGGTTGCTGGAGTGCGGGAGAGGTAAGCGGAATTCCCGGTGTAGCGGTGAAATGCGTAGATATCGGGAGGAACACCTGCGGTGTAGACGGCTTACTGGACCGTAACTGACGCTGAGGCACGAAAGCGTGGGTAGCAAACAGGATTAGATACCCTGGTAGTCCACGCCCTAAACGATGCATACTTGGTGTGGGCAGTTCAGTCTGTCCGTGCCGAAGCTAACGCGATAAGTATGCCGCCTGGGGAGTACGGTCGCAAGGCTG
AASW	PROK_06091	TACGTAGGGGGCAAGCGTTATCCGGATTTACTGGGTGTAAAGGGAGCGTAGACGGAGTGGCAAGTCTGAAGTGAAAACCCTGGGCTTAACCTGGGGACTGCTTTGGAAACTGTTAATCTAGAGTGTTGGAGAGGTAAGTGGAATTCCTGGTGTAGCGGTGAAATGCGTAGATATCAGGAAGAACACCGGAGGCGAAGGCGGCTTACTGGACAATAACTGACGTTGAGGCTCGAAAGCGTGGGGATCAAACAGGATTAGATACCCTGGTAGTCCACGCTGTAAACGATGAATACTAGGTGTCGGGGTGCAAAGCATCTCGGTGCCGTCGCTAACGCAATAAGTATTCCACCTGGGGAGTACGTTCGCAAGAATG
AASW	EUK_0048	TTCCAGCTCCAATAGCGTATATTAAAGTTGTTGCAGTTAAAAAGCTCGTAGTTGAACCTTGGGTCTGGCTGGCCGGTCCGCCTCACCGCGAGTACTGGTCCGGCTGGACCTTTCCTTCTGGGGAACCTCATGGCCTTCACTGGCTGTGGGGGGAACCAGGACTTTTACTGTGAAAAAATTAGAGTGTTCAAAGCAGGCCTTTGCTCGAATACATTAGCATAGGATGTTTTCATTAATCAGGGAACGAAAGTTAGGGGATCGAAGACGATCAGATACCGTCGTAGTCTTAACCATAAACTATGCCGACTAGGGATCGGGCGGTGTTTCTATGATGACCCGCTCGGCACCTTACGAGAAATCAAAGTTTTTGGGTTCTGGGGGGAGTATGGTCGCAAGGCTG
AASW	PROK_03214	TACGGAGGGTGCAAGCGTTATCCGGATTTACTGGGTTTAAAGGGTGCGCAGGCGGTAATTTAAGTCAGTGGTGAAAGCCTACAGCTCAACTGTAGAACTGCCATTGAAACTGAAATACTTGAGTGTGGTAGAGGTAGGCGGAATGAGTCATGTAGCGGTGAAATGCATAGATATGACTCAGAACACCGATTGCGAAGGCAGCTTACTAAACCATTACTGACGCTCAGGCACGAAAGCGTGGGGAGCGAACAGGATTAGATACCCTGGTAGTCCACGCCCTAAACGATGTTCACTCGATATTTGCGATAAACTGTAAGTGTCCAAGCGAAAGCGTTAAGTGAACCACCTGGGGAGTACGATCGCAAGGTTG
AASW	PROK_01171	TACGGAGGGGGTTAGCGTTGTTCGGAATTACTGGGCGTAAAGCGCACGTAGGCGGACTATTAAGTAGAGGGTGAAATCCCAGGGCTCAACCCTGGAACTGCCTTCTATACTGGTAGTCTTGAGTTCGAGAGAGGTGAGTGGAATTCCGAGTGTAGAGGTGAAATTCGTAGATATTCGGAGGAACACCAGTGGCGAAGGCGGCTCACTGGCTCGATACTGACGCTGAGGTGCGAAAGCGTGGGGAGCAAACAGGATTAGATACCCTGGTAGTCCACGCCGTAAACGATGAATGCCAGACGTTGGGGGGCTTGCCCTTCAGTGTCACACTTAACGGATTAAGCATTCCGCCTGGGGAGTACGGTCGCAAGATTA
AASW	PROK_03613	TACGAAGGGGGCTAGCGTTGCTCGGAATCACTGGGCGTAAAGCGCACGTAGGCGGATTTTTAAGTCAGGGGTGAAATCCTGGAGCTCAACTCCAGAACTGCCTTTGATACTGGGAATCTCGAGTCCGGAAGAGGTGAGTGGAACTGCGAGTGTAGAGGTGAAATTCGTAGATATTCGCAAGAACACCAGTGGCGAAGGCGGCTCACTGGTCCGGTACTGACGCTGAGGTGCGAAAGCGTGGGGAGCAAACAGGATTAGATACCCTGGTAGTCCACGCCGTAAACGATGGATGCTAGCCGTTGGCCGGTTTACCGGTCAGTGGCGCAGCTAACGCTTTAAGCATCCCGCCTGGGGAGTACGGTCGCAAGATTA

AASW	PROK_02019	TACGTATGTCGCAAGCGTTATCCGGATTTATTGGGCGTAAAGCGCGTCTAGGCGGTTTGATAAGTCTGATGTGAAAATGCGGGGCTCAACTCCGTATTGCGTTGGAAACTGTCAAACTAGAGTACTGGAGAGGTGGGCGGAACTACAAGTGTAGAGGTGAAATTCGTAGATATTTGTAGGAATGCCGATGGAGAAGTCAGCTCACTGGACAGATACTGACGCTAAAGCGCGAAAGCGTGGGTAGCAAACAGGATTAGATACCCTGGTAGTCCACGCCGTAAACGATGATTACTAGGTGTTGGGGGTCGAACCTCAGCGCCCAAGCTAACGCGATAAGTAATCCGCCTGGGGAGTACGTACGCAAGTATG
AASW	PROK_04801	TACAGAGGGTGCAAGCGTTGTTCGGAATTATTGGGCGTAAAGGGCGCGTAGGCGGTTCGGCAAGTCTCTAGTGAAATCTCAGGGCTCAACTCTGAGTCTGCTGGGGAAACTGCCGGGCTTGAGTGTGGGAGAGGAGAGTGGAATTTCCGGTGTAGCGGTGAAATGCGTAGATATCGGAAGGAACACCTGTGGCGAAAGCGGCTCTCTGGACCACAACTGACGCTGAGGCGCGAAAGCTAGGGGAGCAAACAGGATTAGATACCCTGGTAGTCCTAGCCCTAAACGATGATTGCTTGCTGTGATCGGTACCCAATCCGATTGTGGCGAAGCTAACGCGATAAGCAATCCGCCTGGGGAGTACGGTCGCAAGGCTG
AASW	PROK_00518	TACGGAGGGTCCGAGCGTTATCCGGAATCATTGGGTTTAAAGGGTCCGCAGGCGGTCAATTAAGTCAGAGGTGAAATCCCATAGCTCAACTATGGAACTGCCTTTGATACTGGTTGACTTGAGTCATATGGAAGTAGATAGAATGTGTAGTGTAGCGGTGAAATGCATAGATATTACACAGAATACCGATTGCGAAGGCAGTCTACTACGTATGTACTGACGCTGAGGGACGAAAGCGTGGGGAGCGAACAGGATTAGATACCCTGGTAGTCCACGCCGTAAACGATGGATACTAGTTGTTGGGCATTAGCTCAGTGACTAAGCGAAAGTGATAAGTATCCCACCTGGGGAGTACGGTCGCAAGACTG
AASW	PROK_03718	TACGGAGGGTGCAAGCGTTATCCGGATTCACTGGGTTTAAAGGGTGCGTAGGAGGGCAGGGAAGTCAGTGGTGAAATCCTAGGGCTTAACCCTAGAACTGCCGTTGATACTCTCTGTCTTGAATGCCGTGGAGGTGAGCGGAATATGTCATGTAGCGGTGAAATGCTTAGATATGACATAGAACACCGATTGCGAAGGCAGCTCGCTACACGAACATTGACTCTGAGGCACGAAAGCGTGGGGATCAAACAGGATTAGATACCCTGGTAGTCCACGCCCTAAACGATGGATACTCGACATACGCGATACACGGTGTGTGTCTGAGCGAAAGCATTAAGTATCCCACCTGGGAAGTACGACCGCAAGGTTG
AASW	PROK_07543	TACGTAGGGTCCAAGCGTTAATCGGAATTACTGGGCGTAAAGCGTGCGCAGGCGGTTTTGTAAGACCGATGTGAAATCCCCGGGCTCAACCTGGGAACTGCATTGGTGACTGCAAGGCTTGAGTGTGTCAGAGGGAGGTGGAATTCCGCGTGTAGCAGTGAAATGCGTAGATATGCGGAGGAACACCGATGGCGAAGGCAGCCTCCTGGGATAACACTGACGCTCATGCACGAAAGCGTGGGGAGCAAACAGGATTAGATACCCTGGTAGTCCACGCCCTAAACGATGTCTACTAGTTGTCGGGAATTAATTTTCTTGGTAACGCAGCTAACGCGGGAAGTAGACCGCCTGGGGAGTACGGTCGCAAGATTA
AASW	PROK_02729	TACGTAGGGGGCAAGCGTTGTCCGGATTTACTGGGCGTAAAGCGCGTGTAGGCGGGAACGAAAGTCCGGTGTGAAAGCCCCCCGCTCAACGGGGGAAGGTCGCTGGAAACTGCGTTTCTTGAGGGCGGCAGAGGGAAGTGGAATTGCTGGTGTAGCGGTGAAATGCGTAGAGATCAGCAGGAACACCCGTGGCGAAGGCGGCTTCCTGGGCCGCGCCTGACGCTGAGACGCGAAAGCGTGGGGAGCGAACGGGATTAGATACCCCGGTAGTCCACGCCGTAAACGATGGTCACTAGGTGTATGGGGTATCGACCCCCCATGGGCCGCAGCTAACGCAATAAGTGACCCGCCTGGGGAGTACGGCCGCAAGGTTG
AASW	PROK_04507	TACGGAGGGTGCAAGCGTTATCCGGAATCACTGGGTTTAAAGGGTGCGTAGGCGGCCTGACAAGTCAGAGGTGAAATACACGAGCTTAACTGGTGAATTGCCTTTGAAACTGTCAAGCTAGAATTATGTTGAGGTTAGCGGAATGAGTCATGTAGCGGTGAAATGCATAGATATGACTTAGAACACCAATTGCGTAGGCAGCTAGCTAGGCATATATTGACGCTGAGGCACGAAAGCGTGGGGAGCGAACAGGATTAGATACCCTGGTAGTCCACGCCCTAAACGATGTTTACTCGATGTTTGGGCTTCGGCTTGAGCATCCAAGGGAAACCATTAAGTAAACCACCTGGGGAGTACGTTCGCAAGAATG
AASW	PROK_06672	TACGAAGGGGGCTAGCGTTGTTCGGAATTACTGGGCGTAAAGCGCACGTAGGCGGGTTGTTAAGTCAGGGGTGAAATCCCGGAGCTCAACTCCGGAACTGCCTTTGATACTGGCAACCTAGAGGCCGGAAGAGGTTAGTGGAATTCCCAGTGTAGAGGTGAAATTCGTAGATATTGGGAAGAACACCAGTGGCGAAGGCGGCTAACTGGTCCGGTACTGACGCTGAGGTGCGAAAGCGTGGGGAGCAAACAGGATTAGATACCCTGGTAGTCCACGCCGTAAACTATGGATGCTAGCCGTCAGCGGGCTTGCCCGTTGGTGGCGCAGCTAACGCATTAAGCATCCCGCCTGGGGAGTACGGTCGCAAGATTA
AASW	PROK_07515	TACGGAGGGGGTTAGCGTTGTTCGGAATTACTGGGCGTAAAGCGCACGTAGGCGGACTAATAAGTTAGAGGTGAAATCCCAGGGCTCAACCCTGGAACTGCCTTTAATACTGTTAGTCTAGAGTTCGAGAGAGGTAAGTGGAATTCCGAGTGTAGAGGTGAAATTCGTAGATATTCGGAGGAACACCAGTGGCGAAGGCGGCTTACTGGCTCGATACTGACGCTGAGGTGCGAAAGTGTGGGGAGCAAACAGGATTAGATACCCTGGTAGTCCACACCGTAAACGATGAATGCCAGACGTCAGGGGGCTTGCCCTTTGGTGTCACACCTAACGGATTAAGCATTCCGCCTGGGGAGTACGGTCGCAAGATTA
AASW	PROK_04170	GACGAACCGTGCGAACGTTGTTCGGAATCACTGGGCTTAAAGGGCGCGTAGGCGGACCGCCAAGTCCGTGGTGAAATCCTCCAGCTCAACTGGAGAACTGCCGCGGATACTGGAGGTCTCGAGGGAGGTAGGGGCAAGCGGAACTGTGGGTGGAGCGGTGAAATGCGTTGATATCCACAGGAACTCCGGTGGCGAAGGCGGCTTGCTGGACCTTTTCTGACGCTGAGGCGCGAAAGCCAGGGGAGCAAACGGGATTAGATACCCCGGTAGTCCTGGCCCTAAACGATGGGTACTAGATAGTAGACTAGACATGGGTTTACTGTCGAAGCAAAAGTGCTAAGTACCCCGCCTGGGGAGTATGGTCGCAAGGCTG
AASW	PROK_03363	TACGAAGGTCCCGAGCGTTATTCGGAATCACTGGGCGTAAAGGGAGCGTAGGCGGCGCGGTAAGTCAGATGTGAAATCTCAGAGCTCAACTCTGAAACTGCATTCGATACTGCCGTGCTAGAGTAATGGAGAGGTAACTGGAATTTTTGGTGTAGCAGTGAAATGCGTAGATATCAAGAGGAAGACCAATGGCGAAAGCAGGTTACTGGACATTTACTGACGCTGAGGCTCGAAGGCTAGGGTAGCGAAAGGGATTAGATACCCCTGTAGTCCTAGCAGTAAACGATGTGCGCTTGGTGTGGGAGGGTTCGACCCCTTCCGTGCCGGAGCTAACGCGTTAAGCGCACCGCCTGGGGAGTACGGTCGCAAGACTA
AASW	PROK_05647	TACGAAGGGGGCGAGCGTTGTTCGGAATTACTGGGCGTAAAGGGCGCGTAGGCGGCGGATCAAGTCGAGTGTGAAAGCCCCGGGCTCAACCTGGGAGGTGCGCTCGAGACTGGTTTGCTTGAGTTCGGGAGAGGAGCGTGGAATTCCCAGTGTAGAGGTGAAATTCGTAGATATTGGGAAGAACACCGGTGGCGAAGGCGGCGCTCTGGACCGAGACTGACGCTGAGGCGCGAAAGCGTGGGGAGCAAACAGGATTAGATACCCTGGTAGTCCACGCGGTAAACGATGTGTGCTAGACGTTGGGGAGCCTAGCTCTTCGGTGTCGCAGCCAACGCTGTAAGCACACCGCCTGGGGAGTACGGCCGCAAGGTTA
AASW	PROK_02139	TACGGAGGATGCAAGCGTTATCCGGATTTATTGGGTTTAAAGGGTCCGTAGGCGGAATTTTAAGTCAGTGGTGAAAGCCTGCAGCTCAACTGTAGAACTGCCATTGATACTGAAATTCTTGAATATATATGAAGTGGGCGGAATGCGTCATGTAGCGGTGAAATGCACAGATATGACGCAGAACACCAATTGCGAAGGCAGCTCACTAATGTATTATTGACGCTGAGGGACGAAAGCATGGGGAGCGAACAGGATTAGATACCCTGGTAGTCCATGCCGTAAACGATGATTACTCGGTATTAGTCTGACTAGTGCCTAAGCGAAAGTGATAAGTAATCCACCTGGGGAGTACGCTCGCAAGAGTG
AASW	PROK_01053	TACGGAGGGTGCAAGCGTTATCCGGATTCACTGGGTTTAAAGGGTGCGTAGGTGGGTTGGTAAGTCAGTGGTGAAATCTCCAGGCTTAACTTGGAAACTGCCATTGATACTATCAATCTTGAATACCGTGGAGGTCAGCGGAATATGTCATGTAGCGGTGAAATGCTTAGATATGACATAGAACACCAATTGCGAAGGCAGCTGGCTACACGAATATTGACACTGATGCACGAAAGCGTGGGGATCAAACAGGATTAGATACCCTGGTAGTCCACGCCCTAAACGATGGATACTCGACATACGCGATATACTGTGTGTGTCTGAGCGAAAGCATTAAGTATCCCACCTGGGAAGTACGATCGCAAGATTG
CDW	PROK_05932	TACGGAGGGGGCTAGCGTTGTTCGGAATTACTGGGCGTAAAGCGCACGTAGGCGGCTTTGTAAGTTAGAGGTGAAAGCCCAGGGCTTAACCCTGGAATTGCCTTTAAGACTGCATCGCTAGAATTGTGGAGAGGTAAGTGGAATTCCGAGTGTAGAGGTGAAATTCGTAGATATTCGGAAGAACACCAGTGGCGAAGGCGACTTACTGGACACATATTGACGCTGAGGTGCGAAAGCGTGGGGAGCAAACAGGATTAGATACCCTGGTAGTCCACGCCGTAAACGATGATGACTAGCTGTCGGGGCGCTTAGCGTTCCGGTGGCGCAGCTAACGCGTTAAGTCATCCGCCTGGGGAGTACGGCCGCAAGGTTA

CDW	PROK_03369	TACGTAGGGTGCAAGCGTTAATCGGAATTACTGGGCGTAAAGCGTGCGCAGGCGGTTTTGTAAGTCTGACGTGAAATCCCCGGGCTTAACCTGGGAATTGCGTTGGAGACTGCAAGGCTAGAATCTGGCAGAGGGGGG TAGAATTCCACGTGTAGCAGTGAAATGCGTAGAGATGTGGAGGAACACCGATGGCGAAGGCAGCCCCCTGGGTCAAGATTGACGCTCATGCACGAAAGCGTGGGGAGCAAACAGGATTAGATACCCTGGTAGTCCACG CCCTAAACGATGTCTACTAGTTGTCGGGTTTTAATTAACTTGGTAACGCAGCTAACGCGTGAAGTAGACCGCCTGGGGAGTACGGTCGCAAGATTA
CDW	PROK_06577	TACCGGCGCCTCAAGTGGTAGTCGCTTTTATTGGGCCTAAAACGTCCGTAGCCGGTTTGGTACATTCGTGGGTAAATCAAGGCGCTCAACGTCTTGAATTCTGCGAGCACGGCCAGACTTGGGACCGGGTGAGGTGTGG GGTACTCTCAGGGTAGGGGTAAAATCCTGTCATCCTGAGAGGACCACCTGTTGCGAAGGCGCCACACTAGAACGGATCCGACGGTCAGGGACGAAGCCTAGGGGCACGAACCGGATTAGATACCCGGGTAGTCCTAGG TGTAAACGCTGTGGACTTGATGTTGGGAGTGCTCCGAGCACCCTCAGTGTCGAAGCGAAGGTGATAAGTCCACTGCCTGGGGAGTACGGTCGCAAGGCTG
CDW	PROK_01387	TACGGAGGGTGCAAGCGTTAATCGGAATTACTGGGCGTAAAGCGCACGCAGGCGGTCTGTTAAGTCAGATGTGAAATCCCCGGGCTTAACCTGGGAACTGCATTTGAAACTGGCAGGCTTGAGTCTCGTAGAGGGGGG TAGAATTCCAGGTGTAGCGGTGAAATGCGTAGAGATCTGGAGGAATACCGGTGGCGAAGGCGGCCCCCTGGACGAAGACTGACGCTCAGGTGCGAAAGCGTGGGGAGCAAACAGGATTAGATACCCTGGTAGTCCAC GCCGTAAACGATGTCGACTTGGAGGCTGTTCCCCTGAGGAGTGGCTTCCGGAGCTAACGCGTTAAGTCGACCGCCTGGGGAGTACGGCCGCAAGGTTA
CDW	PROK_04212	TACGTAGGGTGCAAGCGTTAATCGGAATTACTGGGCGTAAAGCGTGCGCAGGCGGTTTTGTAAGTCTGATGTGAAAGCCCCGGGCTCAACCTGGGAATTGCATTGGAGACTGCAAGGCTTGAATCTGGCAGAGGGGGG TAGAATTCCACGTGTAGCAGTGAAATGCGTAGAGATGTGGAGGAACACCGATGGCGAAGGCAGCCCCCTGGGTCAAGATTGACGCTCATGCACGAAAGCGTGGGGAGCAAACAGGATTAGATACCCTGGTAGTCCACG CCCTAAACGATGTCTACTAGTTGTCGGGTTTTAATTAACTTGGTAACGCAGCTAACGCGTGAAGTAGACCGCCTGGGGAGTACGGTCGCAAGATTA
CDW	PROK_09655	GACGAACCGTGCGAACGTTATTCGGAATCACTGGGCTTAAAGCGCGTGTAGGCGGGCCGGCACGTCGGCCGCTGAAAGCCCCCGGCTCAACCGGGGAAGCGGCGCCGATACGACCGGCCTGGAGGGGGAGAGGGGG GCCTGGAACTCCCGGTGGAGCGGTGAAATGCGTTGAGATCGGGAGGAACGCCCGCGGCGAAAGCGAGGCCCTGGATCCTTTCTGACGCTGAGACGCGAAAGCCAGGGGAGCGAACGGGATTAGATACCCCGGTAGTC CTGGCCGTAAACGATGGGCACCGGGTAGGGGGCTCGCCGATGGGCCCCCTGCCGCAGCGAAAGCGTGAAGTGCCCCGCCTGGGGAGTATGGTCGCAAGGCTG
CDW	PROK_00551	GACGAACCGTGCGAACGTTGTTCGGAATCACTGGGCTTAAAGGGCGCGTAGGCGGGCCGCCGAGTCAGGGGTGAAATCCTCCCGCTCAACGGGAGAACAGCTTCTGATACTGGCGGCCTCGAGGGGGGTAGGGGCAT GCGGAACTTCCGGTGGAGCGGTGAAATGCGTAGATATCGGAAGGAACGCCGGTGGCGAAAGCGGCGTGCTGGACCCTTTCTGACGCTGAGGCGCGAAAGCCAGGGGAGCAAACGGGATTAGATACCCCGGTAGTCCT GGCCCTAAACGATGGGTACTAGGTTGTGGACTGACATGGGTTCACAGCCGAAGCAAAAGTGCTAAGTACCCCGCCTGGGGAGTATGGTCGCAAGGCTG
CDW	PROK_00195	TACGTAGGTGGCAAGCGTTGTCCGGAATTATTGGGCGTAAAGCGCGCGCAGGCGGATTGGTCAGTCTGTCTTAAAAGTTCGGGGCTTAACCCCGTGATGGGATGGAAACTGCCAATCTAGAGTATCGGAGAGGAAAGT GGAATTCCTAGTGTAGCGGTGAAATGCGTAGATATTAGGAAGAACACCAGTGGCGAAGGCGACTTTCTGGACGAAAACTGACGCTGAGGCGCGAAAGCCAGGGGAGCGAACGGGATTAGATACCCCGGTAGTCCTGG CCGTAAACGATGGGTACTAGGTGTAGGAGGTATCGACCCCTTCTGTGCCGGAGTTAACGCAATAAGTACCCCGCCTGGGGAGTACGACCGCAAGGTTG
CDW	PROK_03028	GACGAACCGTGCGAACGTTATTCGGAATCACTGGGCTTAAAGCGCGTGTAGGCGGGGCGGCACGTCGGCCGCTGAAATCCCCCGGCTCAACCGGGGAACGGGCGCCGATACGACCGCCCTGGAGGGGCGTAGGGGGA CCTGGAACTTCCGGTGGAGCGGTGAAATGCGTTGAGATCGGAAGGAACGCCCGTGGCGAAAGCGAGGTCCTGGACGCTGTCTGACGCTGAGACGCGAAAGCCAGGGGAGCGAACGGGATTAGATACCCCGGTAGTCC TGGCCGTAAACGATGGGCACTGGGTAGGGGGCTCGCCGATGGGCTCCCTGCCGGAGCGAAAGCGTGAAGTGCCCCGCCTGGGGAGTATGGTCGCAAGGCTG
CDW	PROK_01874	TACGTAGGTGGCAAGCGTTGTTCGGAATTATTGGGCGTAAAGGGCGCGTAGGCGGTTTGTTAAGTCAGCTGTGAAAGCCCGGGGCTCAACCCCGGAGGGTCAGCTGATACTGGCAGGCTAGAGTACAGAAGAGGTAAC TGGAATTCCCGGTGTAGCGGTGAAATGCGTAGATATCGGGAGGAACACCTGAGGCGAAGGCGGGTTACTGGGCTGATACTGACGCTGATGCGCGAAAGCCAGGGGAGCAAACGGGATTAGATACCCCGGTAGTCCTG GCCCTAAACGATGGATACTTGGTGTGTCGGGTCTTCAAGTCCCGGCGTGCCGGAGCTAACGCGTTAAGTATCCCGCCTGGGGAGTACGGTCGCAAGGCTG
CDW	PROK_01047	TACGGAGGGTGCAAGCGTTATCCGGATTCACTGGGTTTAAAGGGTGCGTAGGCGGGCAGGTAAGTCAGAGGTGAAATCTCCGGGCTTAACCCGGAAACTGCCTTTGATACTATCTGTCTTGAATATGGTGGAGGTAAGC GGAATATGTCATGTAGCGGTGAAATGCTTAGATATGACATAGAACACCCATTGCGAAGGCAGCTTACTACGCCTATATTGACGCTGAGGCACGAAAGCGTGGGGATCAAACAGGATTAGATACCCTGGTAGTCCACGCC CTAAACGATGATTACTCGACATACGCGATACACAGTGTGTGTCTGAGCGAAAGCATTAAGTAATCCACCTGGGAAGTACGACCGCAAGGTTG
CDW	PROK_03266	TACGAAGGGGGCTAGCGTTGCTCGGAATCACTGGGCGTAAAGGGCGCGTAGGCGGATCTTTAAGTCGGGGGTGAAAGCCCAGGGCTCAACCCTGGAATTGCCTTCGATACTGAAGATCTTGAGACCGGAAGAGGTAAG TGGAACTGCGAGTGTAGAGGTGAAATTCGTAGATATTCGCAAGAACACCAGTGGCGAAGGCGGCTTACTGGTCCGGTTCTGACGCTGAGGCGCGAAAGCGTGGGGAGCAAACAGGATTAGATACCCTGGTAGTCCACG CCGTAAACGATGGATGCTAGCCGTTGGCGGGTTTACCCGTCAGTGGCGCAGTTAACGCTTTAAGCATCCCGCCTGGGGAGTACGGTCGCAAGATTA
CDW	PROK_01375	TACGTAGGGTGCAAGCGTTAATCGGAATTACTGGGCGTAAAGCGTGCGCAGGCGGTTTTGTAAGTCTGTCGTGAAATCCCCGGGCTCAACCTGGGAATTGCGATGGAGACTGCAAGGCTGGAGTCTGGCAGAGGGGGG TAGAATTCCACGTGTAGCAGTGAAATGCGTAGAGATGTGGAGGAACACCGATGGCGAAGGCAGCCCCCTGGGTCAAGACTGACGCTCATGCACGAAAGCGTGGGGAGCAAACAGGATTAGATACCCTGGTAGTCCACG CCCTAAACGATGTCTACTAGTTGTCGGGTTTTAATTAACTTGGTAACGCAGCTAACGCGTGAAGTAGACCGCCTGGGGAGTACGGTCGCAAGATTA
CDW	PROK_00647	TACGGAGGGTGCAAGCGTTATCCGGATTCACTGGGTTTAAAGGGTGCGTAGGAGGGCAGGTAAGTCAGTGGTGAAATCTCCGGGCTCAACTCGGAAACTGCCGTTGATACTATCTGTCTTGAATCCCGTGGAGATGAGC GGAATATGTCATGTAGCGGTGAAATGCTTAGATATGACATAGAACACCAATTGCGAAGGCAGCTCGTTACACGAAGATTGACTCTGAGGCACGAAAGCGTGGGGATCAAACAGGATTAGATACCCTGGTAGTCCACGCC CTAAACTATGGATACTCGACATACGCGATACACTGTGTGTGTCTGAGCGAAAGCATTAAGTATCCCACCTGGGAAGTACGACCGCAAGGTTG
CDW	PROK_00554	TACGGGGGGGGCAAGCGTTGTTCGGAATTACTGGGCGTAAAGGGTCGGTAGGTGGCCCGCTAAGTCACACGTGAAATCCCTCAGCTCAACTGGGGAACTGCGTGTGAGACTGGCAGGCTCGAGGGCAGGAGAGGAAC GCGGAATTCCAGGTGTAGCGGTGAAATGCGTAGATATCTGGAGGAACACCGGTGGCGAAGGCGGCGTTCTGGACTGTCTCTGACACTGAACGACGAAAGCCAGGGGAGCAAACGGGATTAGATACCCCGGTAGTCCT GGCCCTAAACGATGAATGCTTGGTGTGACGGGTATCGATCCCTGTCGTGCCGCAGTTAACGCGTTAAGCATTCCGCCTGGGGAGTACGGTCGCAAGGCTG
CDW	EUK_0032	TTCCAGCTCCAATAGCGTATATTAAAGTTGTTGCAGTTAAAAAGCTCGTAGTTGAACCTTGGGTCTGGCTGGCCGGTCCGCCTCACCGCGAGTACTGGTCCGGCTGGACCTTTCCTTCTGGGGAACCTCATGGCCTTCACTG GCTGTGGGGGGAACCAGGACTTTTACTGTGAAAAAATTAGAGTGTTCAAAGCAGGCCTTTGCTCGAATACATTAGCATAAGGACGTTTTCATTAATCAAGAACGAAAGTTAGGGGATCGAAGATGATCAGATACCGTCGT AGTCTTAACCATAAACTATGCCGACTAGGGATCGGGTGTTGTTCTTTTTTTGACGCACTCGGCACCTTACGAGAAATCAAAGTCTTTGGGTTCTGGGGGGAGTATGGTCGCAAGGCTG

CDW	PROK_02085	TACAGAGGGTGCAAGCGTTAATCGGAATTACTGGGCGTAAAGCGTGCGCAGACGGTTGCGTAAGTCAGATGTGAAAGCCCCGGGCTCAACCTGGGAATTGCATTTGAGACTGCGTAGCTAGGGTGCGGAAGAGGGAAGCGGAATTTCCGGTGTAGCGGTGAAATGCGTAGATATCGGAAGGAACACCAGTGGCGAAAGCGGCTTCCTGGTCCAGCACCGACGTTCAGGCACGAAAGCGTGGGGAGCAAACAGGATTAGATACCCTGGTAGTCCACGCCATAAACGATGAGAACTAGACGTTGAAGGGGTAAGCCCTCCAGTGTCGTAGCTAACGCGCTAAGTTCTCCGCCTGGGGAGTACGGCCGCAAGGTTG
CDW	PROK_02447	TACGTAGGTGGCAAGCGTTGTCCGGAATTATTGGGCGTAAAGCGCGCGCAGGTGGTTTCTTAAGTCTGATGTGAAAGCCCACGGCTCAACCGTGGAGGGTCATTGGAAACTGGGGAACTTGAGTGCAGAAGAGGAAAGTGGAATTCCAAGTGTAGCGGTGAAATGCGTAGAGATTTGGAGGAACACCAGTGGCGAAGGCGACTTTCTGGTCTGTAACTGACACTGAGGCGCGAAAGCGTGGGGAGCGAACAGGATTAGATACCCTGGTAGTCCACGCCGTAAACGATGAGTGCTAAGTGTTAGAGGGTTTCCGCCCTTTAGTGCTGCAGCTAACGCATTAAGCACTCCGCCTGGGGAGTACGGTCGCAAGACTG
CDW	PROK_00598	TACAGAGGGGGCAAGCGTTGTTCGGAATTACTGGGCGTAAAGGGCGCGTAGGCGGCCTTCTAAGTCGAACGTGAAATCCCCGGGCTCAACCCGGGAACTGCGTCCGATACTGGGAGGCTTGAATCCGGGAGAGGGATGCGGAATTCCAGGTGTAGCGGTGAAATGCGTAGATATCTGGAGGAACACCGGTGGCGAAGGCGGCATCCTGGACCGGCATTGACGCTGAGGCGCGAAAGCCAGGGGAGCAAACGGGATTAGATACCCCGGTAGTCCTGGCCCTAAACGATGAATGCTTGGTGTGGCGGGTATCGATCCCTGCCGTGCCGAAGCTAACGCATTAAGCATTCCGCCTGGGGAGTACGGTCGCAAGGCTG
CDW	PROK_00489	TACAGAGGGGGCAAGCGTTATTCGGAATTATTGGGCGTAAAGGGCGCGTAGGCGGCTTGGCAAGTGGAAGGTGAAATCCCTCGGCTTAACCGAGGAACTGCCCTCCAAACTGCTTTGCTTGAGGCCGGGAGAGGCGAGTGGAATTCCCAGTGTAGCGGTGAAATGCGTAGATATTGGGAGGAACACCAGTGGCGAAGGCGGCTCGCTGGACCGGTTCTGACGCTGAGGCGCGAAAGCGTGGGGAGCAAACGGGATTAGATACCCCGGTAGTCCACGCTGTAAACGATGAGTGCTTGGTGTAGCGGGTATCGACCCCTGCTGTGCCGAAGTCAACACATTAAGCACTCCGCCTGGGGAGTACGGTCGCAAGGCTG
CDW	PROK_03425	TACGAAGGGGGCTAGCGTTGCTCGGAATCACTGGGCGTAAAGCGCACGTAGGCGGCTTGTTAAGTCAGGGGTGAAATCCTGGAGCTCAACTCCAGAACTGCCTTTGATACTGGCGAGCTTGAGTCCGGGAGAGGTGAGTGGAACTGCGAGTGTAGAGGTGAAATTCGTAGATATTCGCAAGAACACCAGTGGCGAAGGCGGCTCACTGGCCCGGTACTGACGCTGAGGTGCGAAAGCGTGGGGAGCAAACAGGATTAGATACCCTGGTAGTCCACGCCGTAAACGATGGATGCTAGCCGTTGGCGGGTTTACTCGTCAGTGGCGCAGCTAACGCATTAAGCATCCCGCCTGGGGAGTACGGTCGCAAGATTA
CDW	PROK_08312	GACAGAGGTCTCGAGCGTTAGGCGGAATCACTGGGCTTAAAGCGTGTGTAGGCGGACTTCTAAGTACCTTGTGAAATCCCACGGCTTAACCGTGGAACTGCTTGGTATACTGGTTGTCTTGGGTCATCTAGGGGCTACTGGAACAAACGGTGGAGCGGTGAAATGCGTAGATATCGTTTGGAACGCCAATGGTGAAAACAAGTAGCTGGGGATGCACCGACGCTGAGACACGAAAGCCAGGGGAGCGAACGGGATTAGATACCCCGGTAGTCCTGGCTGTAAACGATGTCGACTAGATCGTGGTGGCTCTGACGCCGTCACGGTCGAAGCAAAAGTGCTAAGTCGACCGCCTGGGAAGTACGGTCGCAAGGCTA
CDW	PROK_01550	TACAGAGGGGGCAAGCGTTATTCGGAATTATTGGGCGTAAAGGGCGCGTAGGCGGCGATTCAAGTGGCGGGTGAAATCCCTCGGCTTAACCGAGGAACTGCCTGCCAGACTGGATTGCTTGAGTCCGGGAGAGGTGAGTGGAATTCCCAGTGTAGCGGTGAAATGCGTAGATATTGGGAGGAACACCAGTGGCGAAGGCGGCTCACTGGACCGGAACTGACGCTGAGGCGCGAAAGCGTGGGGAGCAAACAGGATTAGATACCCTGGTAGTCCACGCCGTAAACGATGAGTGCTTGGTGTAGCGGGTATCGACCCCTGCTGTGCCGAAGTCAACACATTAAGCACTCCGCCTGGGGAGTACGGTCGCAAGACTG
CDW	PROK_03935	TACGAAGGGGGCTAGCGTTGCTCGGAATCACTGGGCGTAAAGGGCGCGTAGGCGGCCATTCAAGTCGGGGGTGAAAGCCCAGGGCTCAACCCTGGAATTGCCTTCGATACTGTTTGGCTAGAGACCGGAAGAGGTAAGTGGAACTGCGAGTGTAGAGGTGAAATTCGTAGATATTCGCAAGAACACCAGTGGCGAAGGCGGCTTACTGGTCCGGTTCTGACGCTGAGGCGCGAAAGCGTGGGGAGCAAACAGGATTAGATACCCTGGTAGTCCACGCTGTAAACGATGGATGCTAGCCGTTGGCCAGCTTGCTGGTCAGTGGCGCAGCTAACGCTTTAAGCATCCCGCCTGGGGAGTACGGTCGCAAGATTA
CDW	PROK_06113	TACGGAGGGGGCTAGCGTTGTTCGGAATTACTGGGCGTAAAGCGCACGTAGGCGGCTTTGTAAGTTAGAGGTGAAAGCCCAGGGCTCAACCCTGGAATTGCCTTTAAGACTGCATCGCTTGAACATCGGAGAGGTAAGTGGAATTCCGAGTGTAGAGGTGAAATTCGTAGATATTCGGAAGAACACCAGTGGCGAAGGCGACTTACTGGACGATTGTTGACGCTGAGGTGCGAAAGCGTGGGGAGCAAACAGGATTAGATACCCTGGTAGTCCACGCCGTAAACGATGATGACTAGCTGTCGGGGCGCTTAGCGTTTCGGTGGCGCAGCTAACGCGTTAAGTCATCCGCCTGGGGAGTACGGCCGCAAGGTTA
CDW	PROK_01786	GACGAACCGTGCGAACGTTATTCGGAATCACTGGGCTTAAAGCGCGTGTAGGCGGGCGGGGACGTCGGTCGCTGAAATCCCCCGGCTCAACCGGGGAACGGGCGCCGAAACGCCCCGTCTCGAGGGACGTAGGGGGACCTGGAACTTCCGGTGGAGCGGTGAAATGCGTTGAGATCGGAAGGAACGCCCGTGGCGAAAGCGAGGTCCTGGACGTCTTCTGACGCTGAGACGCGAAAGCTAGGGGAGCGAACGGGATTAGATACCCCGGTAGTCCTAGCCGTAAACGATGGGCACTGGGTAGGGGGCTCGCCGATGGGCTCCCTGCCGCAGCGAAAGCGTGAAGTGCCCCGCCTGGGGAGTATGGTCGCAAGGCTG
CDW	EUK_0052	TTCCAGCTCCAATAGCGTATATTAAAGTTGTTGCAGTTAAAAAGCTCGTAGTTGAACCTTGGGCTTGGTTGGCCGGTCCGCCTTTTTGGCGAGTACTGGACCCAACCGAGCCTTTCCTTCTGGCTAACCTTTCGCCCTTGTGGTGTTTGGCGAACCAGGACTTTTACTTTGAAAAAATTAGAGTGTTCAAAGCAGGCCTTTGCTCGAATATATTAGCATGAAGGACGTTTTCATTAATCAAGAACGAAAGTTAGGGGATCGAAGATGATCAGATACCGTCGTAGTCTTAACCATAAACTATGCCGACTAGGGATCGGGTGTTGTTCTTTTTTTGACGCACTCGGCACCTTACGAGAAATCAAAGTCTTTGGGTTCTGGGGGGAGTATGGTCGCAAGGCTG
CDW	PROK_02670	TACGAAGGGGGCTAGCGTTGTTCGGAATCACTGGGCGTAAAGGGCGCGTAGGCGGCTTTATAAGTCGGGGGTGAAAGCCTGTGGCTCAACCACAGAATTGCCTTCGATACTGTATGGCTTGAGACCGGAAGAGGTTAGTGGAACTGCGAGTGTAGAGGTGAAATTCGTAGATATTCGCAAGAACACCAGTGGCGAAGGCGGCTAACTGGTCCGGTTCTGACGCTGAGGCGCGAAAGCGTGGGGAGCAAACAGGATTAGATACCCTGGTAGTCCACGCCGTAAACGATGAATGCCAGCCGTTGGCGAGCTTGCTCGTCAGTGGCGCAGCTAACGCTTTAAGCATTCCGCCTGGGGAGTACGGTCGCAAGATTA
CDW	PROK_01323	TACAGAGGGGGCAAGCGTTGTTCGGAATTACTGGGCGTAAAGGGCGCGTAGGCGGCCTTCTAAGTCGAACGTGAAATCCCTGGGCTCAACCCAGGAACTGCGTCCGATACTGGAAGGCTTGAATCCGGGAGAGGGATGCGGAATTCCAGGTGTAGCGGTGAAATGCGTAGATATCTGGAGGAACACCGGTGGCGAAGGCGGCATCCTGGACCGGTATTGACGCTGAGGCGCGAAAGCCAGGGGAGCAAACGGGATTAGATACCCCGGTAGTCCTGGCCCTAAACGATGAATGCTTGGTGTGGCGGGTATCGATCCCTGCCGTGCCGAAGCTAACGCATTAAGCATTCCGCCTGGGGAGTACGGTCGCAAGGCTG
CDW	PROK_00060	TACGTAGGGTGCAAGCGTTAATCGGAATTACTGGGCGTAAAGCGTGCGCAGGCGGTTTTGTAAGTCTGTCGTGAAAGCCCCGGGCTTAACCTGGGAATTGCGATGGAGACTGCAAGGCTTGAATCTGGCAGAGGGGGGTAGAATTCCACGTGTAGCAGTGAAATGCGTAGAGATGTGGAGGAACACCGATGGCGAAGGCAGCCCCCTGGGTCAAGATTGACGCTCATGCACGAAAGCGTGGGGAGCAAACAGGATTAGATACCCTGGTAGTCCACGCCCTAAACGATGTCTACTAGTTGTCGGGTCTTAATTGACTTGGTAACGCAGCTAACGCGTGAAGTAGACCGCCTGGGGAGTACGGTCGCAAGATTA
CDW	PROK_02255	TACGTAGGGTGCAAGCGTTAATCGGAATTACTGGGCGTAAAGCGTGTGTAGGCGGTTCGGAAAGAAAGATGTGAAATCCCAGGGCTCAACCTTGGAACTGCATTTTTAACTGCCGAGCTAGAGTATGTCAGAGGGGGGTAGAATTCCACGTGTAGCAGTGAAATGCGTAGATATGTGGAGGAATACCGATGGCGAAGGCAGCCCCCTGGGATAATACTGACGCTCAGACACGAAAGCGTGGGGAGCAAACAGGATTAGATACCCTGGTAGTCCACGCCCTAAACGATGTCAACTAGCTGTTGGGGCCGTTAGGCCTTAGTAGCGCAGCTAACGCGTGAAGTTGACCGCCTGGGGAGTACGGTCGCAAGATTA

CDW	PROK_03375	TACCGGCGCCTCAAGTGGTAGTCGCTTTTATTGGGCCTAAAACGTCCGTAGCCGGTCTGATACATTCGTGGGTAAATCAACCAGCTTAACTGGTTGAATTCTGCGAGGACGGTCAGACTTGGGACCGGGAGAGGTGTGG<br>GGTACGCTCAGGGTAGGGGTAAAATCCTGTCATCCTGAGCGGACCACCTGTTGCGAAGGCGCCACACTAGAACGGATCCGACGGTCAGGGACGAAGCCTAGGGGCACGAACCGGATTAGATACCCGGGTAGTCCTAGG<br>TGTAAACGCTGTGGACTTGATGTTGGGGGTGCTCCGAGCACTCTCAGTGTCGAAGCGAAGGTGATAAGTCCACTGCCTGGGAAGTACGGTCGCAAGGCTG
CDW	EUK_0126	TTCCAGCTCCAATAGCGTATATTAAAGTTGTTGCAGTTAAAAAGCTCGTAGTTGAACCTTGGGTCTGGCTGGCCGGTCCGCCTCACCGCGAGTACTGGTCCGGCTGGACCTTTCCTTCTGGGGAACCTCATGGCCTTCACTG<br>GCTGTGGGGGGAACCAGGACTTTTACTGTGAAAAAATTAGAGTGTTCAAAGCAGGCCTTTGCTCGAATACATTAGCATGCCAAGGACGCTTCCGTTGATCAGGGGCTAAGGTTAGGGGATCGAAGACGATTAGATACCG<br>TCGTAGTCTTAACAGCAAACTATGCCGACTAGGGATTGGACTTGGCTCTTATGGCTTGTCCAGCACCTTAGCGAAAGCAAAGTTTTTGGGTTCTGGGGGGAGTACGGGACGCAAGGCTG
CDW	PROK_00786	TACGTAGGGGGCGAGCGTTGTCCGGAATTACTGGGCGTAAAGCGCCTGTAGGCGGGAAGTTACGTCGGACGTGAAAGGCGGGGGCTCAACCCCCGAGCGGCGTTCGAAACGGACTTTCTTGAGGGTCGAAGAGGAGC<br>GTGGAATTCCCGGTGTAGCGGTGAAATGCGTAGAGATCGGGAGGAACACCCATGGCGAAGGCAGCGCTCTGGTCGGCATCTGACGCTGAGAGGCGAAAGCGTGGGGAGCGAACGGGATTAGATACCCCGGTAGTCCA<br>CGCCGTAAACGATGGTCACTAGGTGTCAGCGGTATCGACCCCGCTGGTGCCGCAGCTAACGCAGTAAGTGACCCGCCTGGGGAGTACGGCCGCAAGGTTG
CDW	PROK_06615	TACGAAGGGGGCTAGCGTTGCTCGGAATCACTGGGCGTAAAGGGCGCGTAGGCGGCTCGTCAAGTCGGGGGTGAAAGCCTGTGGCTCAACCACAGAATGGCCTTCGATACTGGCGGGCTTGAGACCGGAAGAGGACA<br>GCGGAACTGCGAGTGTAGAGGTGAAATTCGTAGATATTCGCAAGAACACCAGTGGCGAAGGCGGCTGTCTGGTCCGGTTCTGACGCTGAGGCGCGAAAGCGTGGGGAGCAAACAGGATTAGATACCCTGGTAGTCCAC<br>GCTGTAAACGATGAATGCTAGCCGTTGGGGTGCATGCACCGCAGTGGCGCCGCTAACGCATTAAGCATTCCGCCTGGGGAGTACGGTCGCAAGATTA
CDW	PROK_06835	GACGAACCGTGCGAACGTTATTCGGAATCACTGGGCTTAAAGCGCGTGTAGGCGGGCCGGCACGTCGACTGCTGAAAGCCCCCGGCTCAACCGGGGAACGGGCATCGATACGACCGGCCTGGAGGGGGGTAGGGGGA<br>GCCGGAACTCACGGTGGAGCGGTGAAATGCGTTGAGATCGTGAGGAACGCCCGTGGCGAAAGCGGGCTCCTGGACCCCACCTGACGCTGAGACGCGAAAGCCAGGGGAGCGAACGGGATTAGATACCCCGGTAGTCC<br>TGGCCGTAAACCATGAGCACTGGGTAGGGGGCTCGCCGATGGGCTCCCTGCCGCAGCGAAAGCGTGAAGTGCTCCGCCTGGGGAGTATGGTCGCAAGGCTG
CDW	PROK_03697	TACAGAGGGTGCGAGCGTTAATCGGAATTACTGGGCGTAAAGGGCGCGTAGGTGGTTAATTAAGTCAGATGTGAAATCCCCGGGCTTAACCTGGGAATGGCATATGAAACTGGTTGGCTAGAGTTCGGGAGAGGGGA<br>GTGGAATTTCCGGTGTAGCGGTGAAATGCGTAGATATCGGAAGGAACACCAGTGGCGAAGGCGACTTCCTGGCCCGATACTGACACTGAGGCGCGAAAGCGTGGGGAGCGAACAGGATTAGATACCCTGGTAGTCCAC<br>GCTGTAAACGATGAGAACTAGATGTTAGGTGGCTTAGACCATTTAGTATCGCAGCAAACGCGTTAAGTTCTCCGCCTGGGGAGTACGGTCGCAAGATTA
CDW	PROK_03786	TACGAAGGGGGCTAGCGTTGCTCGGAATCACTGGGCGTAAAGCGCACGTAGGCGGATTGCTAAGTCAGGGGTGAAATCCTGGAGCTCAACTCCAGAACTGCCCTTGATACTGGCGGTCTCGAGTCCGGGAGAGGTGAG<br>TGGAACTGCGAGTGTAGAGGTGAAATTCGTAGATATTCGCAAGAACACCAGTGGCGAAGGCGGCTCACTGGCCCGGTACTGACGCTGAGGTGCGAAAGCGTGGGGAGCAAACAGGATTAGATACCCTGGTAGTCCAC<br>GCCGTAAACGATGGATGCTAGCCGTTGGTGGGTTTACCCTTCAGTGGCGCAGCTAACGCATTAAGCATCCCGCCTGGGGAGTACGGTCGCAAGATTA
CDW	PROK_07457	TACGAAGGGGGCTAGCGTTGCTCGGAATCACTGGGCGTAAAGGGCGCGTAGGCGGATTTCTAAGTCGGGGGTGAAAGCCTGAGGCTCAACCTCAGAATTGCCTTCGATACTGGAAATCTTGAGACCGGAAGAGGTGAG<br>TGGAACTGCGAGTGTAGAGGTGAAATTCGTAGATATTCGCAAGAACACCAGTGGCGAAGGCGGCTCACTGGTCCGGTTCTGACGCTGAGGCGCGAAAGCGTGGGGAGCAAACAGGATTAGATACCCTGGTAGTCCACG<br>CCGTAAACGATGGATGCTAGCCGTTGGCGGGTTTACCCGTCAGTGGCGCAGCTAACGCTTTAAGCATCCCGCCTGGGGAGTACGGTCGCAAGATTA
CDW	PROK_06560	TACGAAGGGGGCGAGCGTTGTTCGGATTTACTGGGCGTAAAGGGCGCGCAGGCGGCTTGTTTTGTCAGGCGTGAAAGCCCCGGGCTTAACCTGGGAACTGCGCTTGAGACGGACGGGCTAGAGTTCGGGAGAGGAGA<br>GCGGAATTCCCAGTGTAGAGGTGAAATTCGTAGATATTGGGAAGAACACCGGTGGCGAAGGCGGCTCTCTGGACCGAGACTGACGCTGAGGCGCGAAAGCGTGGGGAGCAAACAGGATTAGATACCCTGGTAGTCCA<br>CGCTGTAAACGATGTGTGCTAGACGTTGGGGAGCCTAGCTCTTCGGTGTCGCAGCTAACGCGGTAAGCACACCGCCTGGGGAGTACGGCCGCAAGGTTA
CDW	PROK_03167	TACGTAGGGTGCGAGCGTTAATCGGAATTACTGGGCGTAAAGCGTGCGCAGGCGGTTCGTGATGTCTGGTGTGAAAGCCCCGGGCTTAACCTGGGAATTGCATTGGAAACTAGCGGGCTGGAGTGTGGCAGAGGGGG<br>GTGGAATTCCACGTGTAGCAGTGAAATGCGTAGAGATGTGGAGGAACACCGATGGCGAAGGCAGCCCCCTGGGCTAACACTGACGCTCATGCACGAAAGCGTGGGGAGCAAACAGGATTAGATACCCTGGTAGTCCAC<br>GCCCTAAACGATGGCAACTGGGTGTTGGGGAAGCGATTTCTCAGTACCGTAGCTAACGCGTGAAGTTGCCCGCCTGGGGAGTACGGCCGCAAGGTTA
CDW	PROK_02467	TACGGAGGGGGCGAGCGTTATTCGGAATTATTGGGCGTAAAGGGCGCGTAGGCGGCTATTCAAGTCAAAGGTGAAATCCCTCGGCTCAACTGAGGAACTGCCTTTGAAACTGGATGGCTTGAGGCCGGGAGGGGGTA<br>GCGGAATTCCCAGTGTAGCGGTGAAATGCGTAGATATTGGGAGGAACACCAGTGGCGAAGGCGGCTACCTGGACCGGTTCTGACGCTGATGCGCGAAAGCGTGGGTAGCAAACAGGATTAGATACCCTGGTAGTCCAC<br>GCTGTAAACGATGGGTGCTCGGTGTCGCGGGTATCGACCCCTGCGGTGCCTTAGCCAACGCGTTAAGCACCCCGCCTGGGGAGTACGGTCGCAAGGCTG
CDW	PROK_00790	TACGGGGGGGGCAAGCGTTGTTCGGAATTACTGGGCGTAAAGGGCTCGTAGGCGGCCAACTAAGTCAGACGTGAAATCCCCAGGCTCAACCTGGGAACTGCGTCTGATACTGGATGGCTTGAATCCGGGAGAGGGATG<br>CAGAATTCCAGGTGTAGCGGTGAAATGCGTAGATATCTGGAGGAATACCGGTGGCGAAGGCGGCATCCTGGACCGGCATTGACGCTGAGGAGCGAAAGCTAGGGGAGCAAACGGGATTAGATACCCCGGTAGTCCTA<br>GCCCTAAACGATGAATGCTTGGTGTAACGGGTATCGATCCCTGTTGTGCCGAAGCTAACGCATTAAGCATTCCGCCTGGGGAGTACGGTCGCAAGGCTG
CDW	PROK_02328	TACGAGGGGGGCGAGCGTTGTTCGGAATTATTGGGCGTAAAGGGCGCGTAGGCGGTGCGGTAAGTCACCTGTGAAAACTCTGGGCTCAACTCAGAGCCTGCAGGCGAAACTGCCGTGCTTGAGTGTGGGAGAGGTGC<br>GTGGAATTCCCGGTGTAGCGGTGAAATGCGTAGATATCGGGAGGAACACCTGTGGCGAAAGCGGCGCACTGGACCACAACTGACGCTGAGGCGCGAAAGCTAGGGGAGCAAACAGGATTAGATACCCTGGTAGTCCT<br>AGCCCTAAACTATGGATGCTTGGTGTGATCGGTACCCAATCCGATCGTGCCGTAGCTAACGCGTTAAGCATCCCGCCTGGGGAGTACGGTCGCAAGGCTG
CDW	PROK_02396	TACGTAGGTGGCAAGCGTTGTCCGGATTTATTGGGCGTAAAGCGAGCGCAGGCGGTTTCTTAAGTCTGATGTGAAAGCCTTCGGCTTAACCGGAGAAGTGCATCGGAAACTGGGAGACTTGAGTGCAGAAGAGGACAG<br>TGGAACTCCATGTGTAGCGGTGGAATGCGTAGATATATGGAAGAACACCAGTGGCGAAGGCGGCTGTCTAGTCTGTAACTGACGCTGAGGCTCGAAAGCATGGGTAGCAAACAGGATTAGATACCCTGGTAGTCCATG<br>CCGTAAACGATGAGTGCTAGGTGTTGGAGGGTTTCCGCCCTTCAGTGCCGCAGCTAACGCATTAAGCACTCCGCCTGGGGAGTACGACCGCAAGGTTG
CDW	PROK_03041	TACGTAGGCAGCGAGCGTTGTTCGGAGTTACTGGGCGTAAAGGGTGTGTAGGCGGTTGTTTAAGTTTGGTGTGAAATCTCCCGGCTCAACTGGGAGGGTGCGCCGAATACTGAATGACTTCGAGTGCGGGAGAGGAGA<br>GTGGAATTCCTGGTGTAGCGGTGAAATGCGTAGATATCAGGAGGAACACCGGTGGTGTAGACGGCTCTCTGGACCGTAACTGACGCTGAGACACGAAAGCGTGGGTAGCAAACAGGATTAGATACCCTGGTAGTCCAC<br>GCCCTAAACGATGCATATTTGGTGTGGGCAGTTCATTCTGTCCGTGCCGGAGCTAACGCGTTAAATATGCCGCCTGGGGAGTACAGTCGCAAGGCTG

CDW	PROK_05910	TACGTAGGGTGCAAGCGTTAATCGGAATTACTGGGCGTAAAGCGTGCGCAGGCGGTTTTGTAAGTCAGATGTGAAATCCCCGGGCTTAACCTGGGAACTGCGTTTGAAACTACAAGGCTAGAGTGTGGCAGAGGGGGGTGGAATTCCACGTGTAGCAGTGAAATGCGTAGAGATGTGGAGGAACACCGATGGCGAAGGCAGCCCCCTGGGTTAACACTGACGCTCAGGCACGAAAGCGTGGGGAGCAAACAGGATTAGATACCCTGGTAGTCCACGCCCTAAACGATGTCAACTAGTTGTCGGGTCTTAACGGACTTGGTAACGCAGCTAACGCGTGAAGTTGACCGCCTGGGGAGTACGGTCGCAAGATTA
CDW	PROK_01527	GACAGAGGGGGCAAGCGTTGCTCGGAATCACTGGGCGTAAAGGGCGCGTAGGCGGGATGGCAAGTCGGTCGTGAAATCCTTCGGCCCAACCGAAGAACGTCGGCCGATACTGCCGTTCTTGAGGGATGCAGAGGAGACTGGAATTCCCGGTGTAGCGGTGAAATGTGTAGAGATCGGGAGGAACACCGGTGGCGAAGGCGGGTCTCTGGGCATTTCCTGACGCTGAGGCGCGAAAGCGTGGGGAGCAAACGGGATTAGATACCCCGGTAGTCCACGCTGTAAACGATGACGACTAGGTGTGGGGGGGGTTAGTCCCTTCCGTGCCGCAGCTAACGCATTAAGTCGTCCGCCTGGGGAGTACGGCCGCAAGGTTG
CDW	PROK_04067	TACGAAGGGGGCTAGCGTTGTTCGGAATCACTGGGCGTAAAGGGCGCGTAGGCGGCTTGATAAGTCGGGGGTGAAAGCCTGTGGCTCAACCACAGAATTGCCTTCGATACTGTCTGGCTTGAGACCGGAAGAGGTAAGTGGAACTGCGAGTGTAGAGGTGAAATTCGTAGATATTCGCAAGAACACCAGTGGCGAAGGCGGCTTACTGGTCCGGTTCTGACGCTGAGGCGCGAAAGCGTGGGGAGCAAACAGGATTAGATACCCTGGTAGTCCACGCCGTAAACGATGAATGCCAGCCGTTGGGCAGCTTGCTGCTCAGTGGCGCAGCTAACGCTTTAAGCATTCCGCCTGGGGAGTACGGTCGCAAGATTA
CDW	PROK_05421	AACCAGCACCTCAAGTGGTCAGGATGATTATTGGGCCTAAAGCATCCGTAGCCGGCTCTGTAAGTTTTCGGTTAAATCTATGCGCTCAACGTATGGGCTGCCGAAAATACTGCAGAGATAGGGAGTGGGAGAGGTAGACGGTACTCGGTAGGAAGGGGTAAAATCCTTTGATCTATCGATGACCACCTGTGGCGAAGGCGGTCTACCAGAACACGTTCGACGGTGAGGGATGAAAGCTGGGGGAGCAAACCGGATTAGATACCCGGGTAGTCCCAGCTGTAAACGATGCAGACTCGATGATGCATTGGCATATAGCCAGTGCAGTGTCGCAGGGAAGCCGTTAAGTCTGCCGCCTGGGAAGTACGTACGCAAGTATG
CDW	PROK_06482	AACGTAGGAGGCAAGCGTTATCCGGATTCACTGGGCGTAAAGCGCGTGCAGGTGGCGCGGTAAGTTGGGCGTGAAATCTCCCGGCTCAACTGGGAGAGGTCGTCCAATACTACCGTGCTTGAGAGCAGAAGAGGAAGATGGAATTCCCGGTGTAGTGGTGAAATGCGTAGATATCGGGAGGAACACCAGTGGCGAAGGCGATCTTCTGGTCTGTTTCTGACACTCAGACGCGACAGCTAGGGTAGTAAACGGGATTAGAGACCCCGGTAATCCTAGCCGTAAACGATGTGAACTTGGCGTTGGTGGGTTAAACTCCATCAGTGCCGTAGCAAACGCGATAAGTTCACCGCCTGGGGACTACGGCCGCAAGGTTA
CDW	PROK_03014	GACGAACCGTGCTAACGTTGTTCGGAATCACTGGGCTTAAAGGGCGCGTAGGCGGGCCGGAAAGTCAGGGGTGAAAGCCCGCGGCTCAACCGCGGAAGCGCCTTTGATACTGCCGGCCTCGAGGGGGGTAGGGGCGTGCGGAACTTCCGGTGGAGCGGTGAAATGCGTAGATATCGGAAGGAACACCGGTGGCGAAAGCGGCGCGCTGGACCCCTTCTGACGCTGAGGCGCGAAAGCCAGGGGAGCAAACGGGATTAGATACCCCGGTAGTCCTGGCCCTAAACGATGGGTACTAGGTAGTGGGCTTACATGGGCTCACTGCCGAAGCAAAAGTGTTAAGTACCCCGCCTGGGGAGTATGGTCGCAAGGCTG
CDW	PROK_07778	TACGGAGGGGGCTAGCGTTGTTCGGAATTACTGGGCGTAAAGCGCACGTAGGCGGCTTTGTAAGTTAGAGGTGAAAGCCCAGGGCTCAACCCTGGAATTGCCTTTAAGACTGCATCGCTTGAACATCGGAGAGGTAAGTGGAATTCCGAGTGTAGAGGTGAAATTCGTAGATATTCGGAAGAACACCAGTGGCGAAGGCGACTTACTGGACGATTGTTGACGCTGAGGTGCGAAAGCGTGGGGAGCAAACAGGATTAGATACCCTGGTAGTCCACGCCGTAAACGATGATGACTAGCTGTCGGGGCTCTTGGAGTTTCGGTGGCGCAGCTAACGCCTTAAGTCATCCGCCTGGGGAGTACGGCCGCAAGGTTA
CDW	PROK_04965	TACGGAGGGGGCTAGCGTTGTTCGGAATTACTGGGCGTAAAGCGCACGTAGGCGGCTTTGTAAGTTAGAGGTGAAAGCCCAGGGCTCAACCCTGGAATTGCCTTTAAGACTGCATCGCTTGAACATCGGAGAGGTAAGTGGAATTCCGAGTGTAGAGGTGAAATTCGTAGATATTCGGAAGAACACCAGTGGCGAAGGCGACTTACTGGACGATTGTTGACGCTGAGGTGCGAAAGCGTGGGGAGCAAACAGGATTAGATACCCTGGTAGTCCACGCCGTAAACGATGATGACTAGCTGTCGGGGCTCTTGGAGTTTCGGTGGCGCAGCTAACGCCTTAAGTCATCCGCCTGGGGAGTACGGCCGCAAGGTTA
CDW	PROK_00856	TACGTAGGCAGCAAGCGTTGTTCGGAGTTACTGGGCGTAAAGAGTGCGTAGGCGGTTTCGCAAGTTTGGTGTGAAATCTCCCGGCTTAACTGGGAGGGTGCGCCGAAAACTGCGGGGCTGGAGTGTGGGAGAGGTAGGCGGAATTCCTGGTGTAGCGGTGAAATGCGTAGATATCAGGAGGAACACCGGTGGTGTAGACGGCTTACTGGACCATGACTGACGCTGAGGCACGAAAGCGTGGGGAGCAAACAGGATTAGATACCCTGGTAGTCCACGCCCTAAACGATGCACACTTGGTGTGAGCGATTCATTTCGTTCGTGCCGGAGCTAACGCGTTAAGTGTGCCGCCTGGGGAGTACGGTCGCAAGGCTG
CDW	PROK_07784	GACAGAGGGGGCAAGCGTTGCTCGGAATTACTGGGCGTAAAGGGCGCGTAGGCGGGATGGCAAGTCGGTCGTGAAATCCCTCGGCTCAACTGAGGAACGTCGATCGATACTGCCGTTCTTGAGGATTGCAGAGGAGACTGGAATTCCCGGTGTAGCGGTGAAATGTGTAGAGATCGGGAGGAACACCGGTGGCGAAGGCGGGTCTCTGGGCAATTCCTGACGCTGAGGCGCGAAAGCGTGGGGAGCAAACGGGATTAGATACCCCGGTAGTCCACGCTGTAAACGATGACGACTAGGTGTGGGGGGGTTCAGTCCCTTCCGTGCCGCAGCTAACGCATTAAGTCGTCCGCCTGGGGAGTACGGCCGCAAGGTTG
CDW	PROK_01520	GACGAACCGTGCGAACGTTGTTCGGAATCACTGGGCTTAAAGGGCGCGTAGGCGGCCCGCCGCGTCCGGGGTGAAATCCTCCCGCTCAACGGGAGAACGGCCTCGGATACGGGCGGGCTGGAGGGGGGTAGGGGCGGGCGGAACTGTGGGTGGAGCGGTGAAATGCGTTGAGATCCACAGGAACTCCGGTGGCGAAGGCGGCCCGCTGGACCCCATCTGACGCTGAGGCGCGAAAGCCAGGGGAGCGAACGGGATTAGATACCCCGGTAGTCCTGGCCCTAAACGTTGAGGACTAGGTAGTAGCCCGGGTATGGGGTTACTGCCGGAGCGAAAGTGCTAAGTCCTCCGCCTGGGGAGTATGGTCGCAAGGCTG
CDW	PROK_03211	TACGTAGGGTGCGAGCGTTAATCGGAATTACTGGGCGTAAAGCGTGCGCAGGCGGTTCGCTGTGTCTGTCGTGAAAGCCCCGGGCTTAACCTGGGAATGGCGATGGAAACTGGCGAGCTGGAGTGCGGCAGAGGGGGGTGGAATTCCGCGTGTAGCAGTGAAATGCGTAGAGATGCGGAGGAACACCGATGGCGAAGGCAGCCCCCTGGGTCGACACTGACGCTCAGGCACGAAAGCGTGGGGAGCAAACAGGATTAGATACCCTGGTAGTCCACGCCCTAAACCATGTCAACTGGGTGTTGGGGAAGCGATTCCCTAGTACCGTAGCTAACGCGTGAAGTTGACCGCCTGGGGAGTACGGCCGCAAGGTTA
CDW	PROK_01980	TACAGAGGGTGCAAGCGTTAATCGGAATTACTGGGCGTAAAGCGCGCGTAGGTGGTTCGTTAAGTTGGATGTGAAATCCCCGGGCTCAACCTGGGAACTGCATTCAAAACTGTCGAGCTAGAGTATGGTAGAGGGTGGTGGAATTTCCTGTGTAGCGGTGAAATGCGTAGATATAGGAAGGAACACCAGTGGCGAAGGCGACCACCTGGACTGATACTGACACTGAGGTGCGAAAGCGTGGGGAGCAAACAGGATTAGATACCCTGGTAGTCCACGCCGTAAACGATGTCAACTAGCCGTTGGGAGCCTTGAGCTCTTAGTGGCGCAGCTAACGCATTAAGTTGACCGCCTGGGGAGTACGGCCGCAAGGTTA
CDW	PROK_01076	TACGTATGTCCCGAGCGTTATCCGGATTTATTGGGCGTAAAGCGAGCGCAGACGGTTTATTAAGTCTGATGTGAAAGCCCGGAGCTCAACTCCGGAATGGCATTGGAAACTGGTTAACTTGAGTGCAGTAGAGGTAAGTGGAACTCCATGTGTAGCGGTGGAATGCGTAGATATATGGAAGAACACCAGTGGCGAAGGCGGCTTACTGGACTGCAACTGACGTTGAGGCTCGAAAGTGTGGGTAGCAAACAGGATTAGATACCCTGGTAGTCCACACCGTAAACGATGAACACTAGGTGTTAGGAGGTTTCCGCCTCTTAGTGCCGAAGCTAACGCATTAAGTGTTCCGCCTGGGGAGTACGACCGCAAGGTTG
CDW	PROK_03212	TACGGAGGGGGCTAGCGTTGTTCGGAATTACTGGGCGTAAAGCGCGCGTAGGCGGCTTTGTAAGTTAGAGGTGAAAGCCCGGAGCTCAACTCCGGAACTGCCTTTAAGACTGCATCGCTAGAATTGTGGAGAGGTGAGTGGAATTCCGAGTGTAGAGGTGAAATTCGTAGATATTCGGAAGAACACCAGTGGCGAAGGCGACTCACTGGACACATATTGACGCTGAGGTGCGAAAGCGTGGGGAGCAAACAGGATTAGATACCCTGGTAGTCCACGCCGTAAACGATGATGACTAGCTGTCGGGGCTCATGGAGTTTCGGTGGCGCAGCTAACGCGTTAAGTCATCCGCCTGGGGAGTACGGCCGCAAGGTTA

CDW	PROK_00344	TACGGAGGGTGCAAGCGTTAATCGGAATTACTGGGCGTAAAGCGCACGCAGGCGGCTTTTTAAGTCGGATGTGAAAGCCCCGGGCTCAACCTGGGAATTGCATCTGATACTGGGAAGCTAGAGTATGTGAGAGGGGGGTAGAATTCCAAGTGTAGCGGTGAAATGCGTAGAGATTTGGAGGAATACCAGTGGCGAAGGCGGCCCCCTGGCACAATACTGACGCTCAGGTGCGAAAGCGTGGGGAGCAAACAGGATTAGATACCCTGGTAGTCCACGCCGTAAACGATGTCTACTAGATGTTCGAGGTCTTGTACTTTGAGTATCGCAGCTAACGCATTAAGTAGACCGCCTGGGGAGTACGGTCGCAAGATTA
CDW	PROK_07211	TACGGAGGGGGCTAGCGTTGTTCGGAATTACTGGGCGTAAAGCGCACGTAGGCGGCTTTGTAAGTTAGAGGTGAAAGCCCGGGGCTCAACTCCGGAACTGCCTTTAAGACTGCATCGCTAGAATTGTGGAGAGGTGAGTGGAATTCCGAGTGTAGAGGTGAAATTCGTAGATATTCGGAAGAACACCAGTGGCGAAGGCGACTCACTGGACACATATTGACGCTGAGGTGCGAAAGCGTGGGGAGCAAACAGGATTAGATACCCTGGTAGTCCACGCCGTAAACGATGATGACTAGCTGTCGGGGCTCTTGGAGTTTCGGTGGCGCAGCTAACGCGTTAAGTCATCCGCCTGGGGAGTACGGCCGCAAGGTTA
GMW	EUK_0010	TTCCAGCTCCAATAGCGTATATTAAAGTTGTTGCAGTTAGAACGCTCGTAGTCGGATTTCGGGGCAGTCCGACCGGTCTGCCGATGGGTATGCACTGGCCGGAGTGTCCTTTTTGCCGGAGACCGTGCCTACTCTTAACTGAGCGGGGGCGGGAGACGGCACTTTTACTTTGAAAAAATCAGAGTGTTTCAAGCAGGCAGCTCGCTCTTGCATGGATTAGAGGGATGTTTTCACTGATCAAGAACGAAAGTTAGGGGATCGAAGATGATCAGATACCGTCGTAGTCTTAACCATAAACCATGCCGACTAGGGATTGGAGGATGTTCACTGATTGACTCCTTCAGCACCTTACGGGAAACTAAAGTCTTTGGGTTCCGGGGGGAGTATGGTCGCAAGGCTG
GMW	PROK_00122	TACGGAGGGTCCAAGCGTTATCCGGAATCATTGGGTTTAAAGGGTGCGCAGGCGGTTTATTAAGTCAGTGGTGAAATCTCGGGGCTCAACCTCGAAACTGCCATTGATACTGATAAACTTGAGTTCAGTTGGAGTAGGCGGAATGTGTAGTGTAGCGGTGAAATGCTTAGATATTACACAGAACACCGATAGCGAAGGCAGCTTACTAAACTGATACTGACGCTGAGGCACGAAAGCGTGGGGAGCGAACAGGATTAGATACCCTGGTAGTCCACGCCGTAAACTATGATTACTCGATATTGGCGATATACTGTCAGTGTCTAAGCGAAAGTGATAAGTAATCCACCTGGGGAGTACGGTCGCAAGATTG
GMW	PROK_01668	AACGTAGGTCACAAGCGTTGTCCGGAATTACTGGGTGTAAAGGGAGCGCAGGCGGGAAGACAAGTTGGAAGTGAAATCCATGGGCTCAACCCATGAACTGCTTTCAAAACTGTTTTTCTTGAGTAGTGCAGAGGTAGGCGGAATTCCCGGTGTAGCGGTGGAATGCGTAGATATCGGGAGGAACACCAGTGGCGAAGGCGGCCTACTGGGCACCAACTGACGCTGAGGCTCGAAAGTGTGGGTAGCAAACAGGATTAGATACCCTGGTAGTCCACACTGTAAACGATGATTACTAGGTGTTGGAGGATTGACCCCTTCAGTGCCGCAGTTAACACAATAAGTAATCCACCTGGGGAGTACGACCGCAAGGTTG
GMW	PROK_04471	TACAGAGGGTGCAAGCGTTAATCGGAATTACTGGGCGTAAAGCGCACGTAGGTGGTTTGGTAAGTTGGATGTGAAATCCCCGGGCTTAACCTGGGAACTGCATTCAATACTGCCTCACTAGAGTCTGGTAGAGGGAAGCGGAATTCCACATGTAGCGGTGAAATGCGTAGATATGTGGAGGAACATCAATGGCGAAGGCAGCTTCCTGGACTGATACTGACACTGAGGTGCGAAAGCGTGGGTAGCAAACAGGATTAGATACCCTGGTAGTCCACGCCCTAAACGATGAGAACTAGATGTTGGGGAGTTTATCTTCCTAGTGTCGCAGCTAACGCGTTAAGTTCTCCGCCTGGGGAGTACGGCCGCAAGGTTG
GMW	PROK_10101	TACGAAGGGGGCTAGCGTTGCTCGGAATCACTGGGCGTAAAGCGCACGTAGGCGGCTTTTTAAGTCAGGGGTGAAATCCTGGAGCTCAACTCCAGAACTGCCTTTGATACTGAGAAGCTTGAGTCCGGGAGAGGTGAGTGGAACTGCGAGTGTAGAGGTGAAATTCGTAGATATTCGCAAGAACACCAGTGGCGAAGGCGGCTCACTGGCCCGGTACTGACGCTGAGGTGCGAAAGCGTGGGGAGCAAACAGGATTAGATACCCTGGTAGTCCACGCTGTAAACGATGGATGCTAGCCGTTGTCGGGTTTACTCGTCAGTGGCGCAGCTAACGCATTAAGCATCCCGCCTGGGGAGTACGGTCGCAAGATTA
GMW	PROK_11095	TACGTAGGTGGCAAGCGTTGTCCGGATTTATTGGGTTTAAAGGGTGCGTAGGTGGTCTATTAAGTCAGTGCTGAAATATCCCGGCTTAACCGGGAGGGTGGCATTGATACTGATGGACTTGAGTGGAGTTGAGGTAGGCGGAATTGACGGTGTAGCGGTGAAATGCTTAGATATCGTCAAGAACACCAATAGCGAAGGCAGCTTACTAAGCTCTAACTGACACTGATGCACGAAAGTGTGGGGATCAAACAGGATTAGATACCCTGGTAGTCCACACTGTAAACGTTGATTACTCGATGTTGGCGATACACAGTCAGCGTCTTAGCGCAAGCGATAAGTAATCCACCTGGGGAGTACGCCGGCAACGGTG
GMW	PROK_07200	TACGAAGGGGGCTAGCGTTGCTCGGAATGACTGGGCGTAAAGGGCGCGTAGGCGGTTTGTACAGTCAGATGTGAAATTCCCGGGCTCAACCTGGGGGCTGCATTTGATACGTATGGACTTGAGTGAGGAAGAGGGTCGTGGAATTCCCAGTGTAGAGGTGAAATTCGTAGATATTGGGAAGAACACCGGTGGCGAAGGCGGCGACCTGGTCCTTTGACTGACGCTGAGGCGCGAAAGCGTGGGGAGCAAACAGGATTAGATACCCTGGTAGTCCACGCTGTAAACGATGTGCGCTGGATGTTGGGCGACCTAGTCGCTCAGTGTCGCAGCTAACGCGCTAAGCGCACCGCCTGGGGAGTACGGCCGCAAGGTTG
GMW	PROK_11193	TACAGAGGTCTCAAGCGTTGTTCGGATTCATTGGGCGTAAAGGGTGCGTAGGTGGCGCTGTAAGTCGGGTGTGAAATTTCGGAGCTTAACTCCGAAACTGCATTCGATACTGCGGTGCTTGAGGACTGGAGAGGAGACTGGAATTCATGGTGTAGCAGTGAAATGCGTAGAGATCATGAGGAAGACCAGTGGCGAAGGCGGGTCTCTGGACAGTTCCTGACGCTGAGGCACGAAGGTCAGGGGAGCAAACGGGATTAGATACCCCGGTAGTCCTGACAGTAAACGGTGCACGTTTGGTGTGGGAGGATTCGACCCCTTCTGCGCCGGAGCTAACGCGTTAAACGTGCCGCCTGGGGAGTACGGTCGCAAGATTA
GMW	PROK_00694	TACGTAGGGGGCTAGCGTTATCCGGATTTACTGGGCGTAAAGGGTGCGTAGGTGGTCTTTCAAGTCGGTGGTTAAAGGCTACGGCTCAACCGTAGTTAGCCTCCGAAACTGGAAGACTTGAGTGCAGGAGAGGAAAGTGGAATTCCCAGTGTAGCGGTGAAATGCGTAGATATTGGGAGGAACACCAGTAGCGAAGGCGGCTTTCTGGACTGCAACTGACACTGAGGCACGAAAGCGTGGGTAGCAAACAGGATTAGATACCCTGGTAGTCCACGCTGTAAACGATGAGTACTAGGTGTCGGGGGTTACCCCCCTCGGTGCCGCAGCTAACGCATTAAGTACTCCGCCTGGGGAGTACGCACGCAAGTGTG
GMW	PROK_08860	TACGTAGGGTGCGAGCGTTGTCCGGAATTACTGGGCGTAAAGAGCTCGTAGGTGGTTTGTCGCGTTGTTCGTGAAATGCCACAGCTTAACTGTGGGCGTGCGGGCGATACGGGCAGACTGGAGTACTGCAGGGGAGATTGGAATTCCTGGTGTAGCGGTGGAATGCGCAGATATCAGGAGGAACACCGGTGGCGAAGGCGGGTCTCTGGGCAGTAACTGACGCTGAGGAGCGAAAGCGTGGGGAGCGAACAGGATTAGATACCCTGGTAGTCCACGCCGTAAACGGTGGGTACTAGGTGTGGGTTTCCTTCCTTGGGATCCGTGCCGTAGCTAACGCATTAAGTACCCCGCCTGGGGAGTACGGCCGCAAGGCTA
GMW	PROK_02165	TACAGAGGTGGCAAGCGTTGTTCGGAATTACTGGGCGTAAAGGGCGCGTAGGCGGCCATCTAAGTCAGACGTGAAATCCCCCGGCTTAACCTGGGAACTGCGTCTGATACTGGACGGCTTGAGTATGGGAGAGGGATGTAGAATTCCAGGTGTAGCGGTGAAATGCGTAGATATCTGGAGGAATACCGGTGGCGAAGGCGGCATCCTGGACCATTACTGACGCTGATGCGCGAAAGCTAGGGGAGCAAACGGGATTAGATACCCCGGTAGTCCTAGCCCTAAACGATGAATGCTTGGTGTGGCGGGTATCGATCCCTGCCGTGCCGAAGCTAACGCATTAAGCATTCCGCCTGGGGAGTACGGTCGCAAGGCTG
GMW	PROK_03897	TACGTAGGTGGCAAGCGTTGTTCGGAATTATTGGGCGTAAAGGGCGCGTAGGCGGCCGGTTAAGTCAGCTGTGAAAGCCCGGGGCTCAACCCCGGAGGGTCGGCTGATACTGGCTGGCTAGAGTACGGAAGAGGTAGCTGGAATTCCCGGTGTAGCGGTGAAATGCGTAGATATCGGGAGGAACACCTGAGGCGAAGGCGGGCTACTGGGCCGATACTGACGCTGATGCGCGAAAGCCAGGGGAGCGAACGGGATTAGATACCCCGGTAGTCCTGGCCCTAAACGATGGACACTTGGTGTGTCGGGTCTTACAGTCCCGGCGTGCCGGAGCTAACGCGTTAAGTGTCCCGCCTGGGGAGTACGGTCGCAAGGCTG
TBW	PROK_00456	TACGGAGGGTCCAAGCGTTATCCGGATTCATTGGGTTTAAAGGGTGCGTAGGCGGCTTGATAAGTCAGTAGTGAAAGACTGTGGCTCAACCATAGAATTGCTATTGAAACTGTCAAGCTTGAATCTAGTTGAAGTGGGCGGAATACATCATGTAGCGGTGAAATGCATAGATATGATGTGGAACGCCGATTGCGAAGGCAGCTCACTAAGTTAGTATTGACGCTGAGGCACGAAAGCGTGGGTAGCGAACAGGATTAGATACCCTGGTAGTCCACGCCGTAAACGATGGTCACTCGCTGTTGGCGACATAATGTCAGTGGCTGAGCGAAAGCATTAAGTGACCCACCTGGGGAGTACGTTCGCAAGAATG

TBW	PROK_00103	TACGGAGGGTGCAAGCGTTATCCGGATTTATTAGGTTTAAAGGGTTCGCAGGCGGAATTTTAAGTCAGTGGTGAAAGCCTACAGCTCAACTGTAGAACTGCCATTGAAACTGATATTCTTGAGTATAGATGAAGTGGGCG<br>GAATATGTCATGTAGCGGTGAAATGCATAGATATGACATGGAACACCAATTGCGAAGGCAGCTCACTAAACTATTACTGACGCTCATGAACGAAAGCGTGGGGAGCAAACAGGATTAGATACCCTGGTAGTCCACGCCG<br>TAAACTATGATCACTAGATGTTGGCGATATATTGTCAGTGTTTTAGAGAAATCGTTAAGTGATCCACCTGGGGAGTACGTTCGCAAGAATG
TBW	PROK_00110	TACGGAGGATGCGAGCGTTATCCGGATTTATTGGGTTTAAAGGGTCCGCAGGCGGACCAATCAGTCAGTGGTGAAATCCAATCGCTTAACGATTGAACTGCCATTGATACTGTTGGTCTTGAGTCTAGTTGACGTAGGCG<br>GAATGTGACATGTAGCGGTGAAATGCATAGATATGTCACAGAACACCAATTGCGAAGGCAGCTTACGAAACTACGACTGACGCTCATGGACGAAAGCGTGGGTAGCGAACAGGATTAGATACCCTGGTAGTCCACGCC<br>GTAAACGATGATCACTCGATGTCAGCGATATACTGTTGGTGTCTTAGCGAAAGTTTTAAGTGATCCACCTGGGGAGTACGTTCGCAAGAATG
TBW	PROK_00085	TACGGAGGGTGCAAGCGTTATCCGGATTTATTAGGTTTAAAGGGTCCGTAGGCGGAAATTTAAGTCAGTGGTGAAAGCCCATAGCTCAACTATGGAACTGCCATTGAAACTGAATTTCTTGAATATGATTGAAGCAGGCG<br>GAATATGTCATGTAGCGGTGAAATGCTTAGATATGACATAGAACACCGATAGCGAAGGCAGCTTGCTAAGTCATTATTGACGCTGAGGGACGAAAGCGTGGGGAGCGAACAGGATTAGATACCCTGGTAGTCCACGCC<br>GTAAACGATGATTACTAGCTATTGGCGATATACTGTCAGTGGCAAAGCGAAAGTGTTAAGTAATCCACCTGGGGAGTACGATCGCAAGGTTG
TBW	PROK_02457	TACGGAGGGGGCTAACGTTGTTCGGAATTACTGGGCGTAAAGCGCGCGTAGGCGGATTAGACAGTTGAGGGTGAAATCCCGGAGCTTAACTCCGGAACTGCCTTCAATACTACTAATCTTGAGTTCGGAAGAGGTGAGT<br>GGAATTCCTAGTGTAGAGGTGAAATTCGTAGATATTAGGAAGAACATCAGTGGCGAAGGCGACTCACTGGTCCGATACTGACGCTGAGGTGCGAAAGCGTGGGGAGCAAACAGGATTAGATACCCTGGTAGTCCACGC<br>CGTAAACGATGGAAGCTAGTTGTCGGGCGGCATGCCGTTCGGTGACACAGCTAACGCATTAAGCTTCCCGCCTGGGGAGTACGATCGCAAGATTA
TBW	PROK_00062	TACGGAGGATCCAAGCGTTATCCGGAATTATTGGGTTTAAAGGGTCCGTAGGTGGACAATTAAGTCAGAGGTGAAATCCTGCAGCTCAACTGTAGAATTGCCTTTGAAACTGGTTGTCTTGAGTCATTATGAAGTGGTTA<br>GAATGTGTAGTGTAGCGGTGAAATGCATAGATATTACACAGAATACCAATTGCGAAGGCAGATCACTAATAATGTACTGACACTGATGGACGAAAGCGTGGGTAGCGAACAGGATTAGATACCCTGGTAGTCCACGCCG<br>TAAACGATGGTTACTAGCTGTTCGGACTTCGGTCTGAGTGGCTAAGCGAAAGTGATAAGTAACCCACCTGGGGAGTACGTTCGCAAGAATG
TBW	PROK_00649	TACATAGGGTGCAAGCGTTAATCGGAATTACTGGGCGTAAAGCGTTCGCAGGCTGTTTTACAAGTCAGATGTGAAATCCCCGAGCTCAACTTGGGAACTGCGTTTGAAACTGTAAGACTAGAGTGTGTCAGAGGGGGGT<br>AGAATTCCACGTGTAGCAGTGAAATGCGTAGATATGTGGAGGAATACCAATGGCGAAGGCAGCCCCCTGGGATAACACTGACGCTCATGAACGAAAGCGTGGGGAGCAAACAGGATTAGATACCCTGGTAGTCCACGC<br>CCTAAACGATGTCTACTAGTTGTTGGTGGAGTAAAATCCATGAGTAACGCAGCTAACGCGTGAAGTAGACCGCCTGGGGAGTACGGTCGCAAGATTA
TBW	PROK_00316	TACGGAGGATGCAAGCGTTATCCGGATTTATTGGGTTTAAAGGGTCCGCAGGCGGATCAATAAGTCAGTGGTGAAAGCCCATAGCTCAACTATGGAACTGCCATTGAAACTGTTGATCTTGAGTCTAGTTGACGTAGGCG<br>GAATGTGACATGTAGCGGTGAAATGCATAGATATGTCACAGAACACCAATTGCGAAGGCAGCTTACGAAACTAGACTGACGCTCATGGACGAAAGCGTGGGTAGCGAACAGGATTAGATACCCTGGTAGTCCACGCCGT<br>AAACGATGATCACTCGATGTCGGCGATATACTGTCGGTGTCCAAGCGAAAGTATTAAGTGATCCACCTGGGGAGTACGTTCGCAAGAATG
TBW	PROK_03146	TACGGGGAGAGTGAGCGTTATTCAGAATGACTGGGCGTAAAGGGTACGTAGGCTGTTTTTTAGGTTGAAGGTGTAAGTTTCAAGCTTTACTTGAAGAATATTTTCAAAACCAAAAATCTAGAGCTTGAGTGAGGAGAGT<br>AGAATTTTTAGTGGAGAGGTGAAATTCGTAGAGATTAAAAGGAATGTCAAGGCGAAAGCAACTCTCTGAATCAAAGCTGACGCTGAGGTACGAAAGCGTAGTTAGCAAGTGGGATTAGAGACCCCATTAGTCTACGCAG<br>TCAACGATGCCGGTTCATCCTTGCGTTAAAAATGCAGGGGTTTAGCTAACGCGTCAAACCGGCCGCCTGAGGACTATGGCCGCAAGGTTA
TBW	PROK_00036	TACGGAGGGTGCAAGCGTTAATCGGAATTACTGGGCGTAAAGCGTGCGTAGGCGGTTTATTAAGTCAGATGTGAAAGCCCCGGGCTTAACCTGGGAACTGCATTTGAAACTGGTCAACTAGAGTATGGTAGAGGAAAG<br>TGGAATTTCTGGTGTAGCGGTGAAATGCGTAGATATCAGAAGGAACATCAATGGCGAAGGCAACTTTCTGGACCAATACTGACGCTGAGGTACGAAAGCGTGGGTAGCAAACAGGATTAGATACCCTGGTAGTCCACGC<br>CGTAAACGATGATAACTAGCCGTTGGGGGGATTTACCCCTTAGTGGCGAAGCTAACGCGTTAAGTTATCCGCCTGGGGAGTACGGCCGCAAGGTTA
TBW	PROK_06319	TACGGAGGGGGTTAGCGTTGTTCGGAATTACTGGGCGTAAAGCGCACGTAGGCGGATTAGTAAGTGAGGGGTGAAATCCCGAGGCTCAACCTCGGAACTGCCTTTCATACTGCTAGTCTAGAGTTCGAGAGAGGTGAG<br>TGGAATTCCGAGTGTAGAGGTGAAATTCGTAGATATTCGGAGGAACACCAGTGGCGAAGGCGGCTCACTGGCTCGATACTGACGCTGAGGTGCGAAAGCGTGGGGAGCAAACAGGATTAGATACCCTGGTAGTCCACG<br>CCGTAAACGATGAGAGCTAGTCGTCGGGGGGCATGCCCTTCGGTGACGCAGTTAACGCATTAAGCTCTCCGCCTGGGGAGTACGGTCGCAAGATTA
TBW	PROK_00232	TACGGAGGGTGCAAGCGTTAATCGGAATTACTGGGCGTAAAGCGCGCGTAGGCGGTTCGGTAAGTTGGATGTGAAAGCCCAGGGCTCAACCTTGGAACTGCATTCAAAACTGCCAGGCTAGAGTACGGTAGAGGGGG<br>GTAGAATTCCACGTGTAGCGGTGAAATGCGTAGAGATGTGGAGGAATACCAGTGGCGAAGGCGGCCCCCTGGATCGATACTGACGCTGAGGTGCGAAAGCGTGGGGAGCAAACAGGATTAGATACCCTGGTAGTCCA<br>CGCTGTAAACGATGTCAACTAGCCGTTGGAGGGGTAATCCCTTTAGTGGCGCAGCTAACGCAATAAGTTGACCGCCTGGGGAGTACGGTCGCAAGATTA
TBW	EUK_0014	TTCCAGCTCTAATAGCGTATATTAAAGTTGTTGCAGTTAAAAAGCTCGTAGTCGGATGTCGGGCTCGGGCAGGCTGTCGGCTTCGGTCGGACGGCAGGCTCGGGTCTTTCTGCCTGAGGATCCATTTGCACTTTATTGTGG<br>AGGTGGGGACGCAGGCCGTTTACTTTGAAAAAATTAGAGTGTTCAAAGCAGGCCTACGCTTGAATACATTAGCATGGAACAAGGATGTTTTCATTGATCAAGAACGAAAGTTAGGGGATCGAAGACGATCAGATACCGT<br>CGTAGTCTTAACCATAAACTATGCCGACTAGGGATCAGTGGATGTCAATTTGCGACTCCATTGGCACCTTGTGAGAAATCAAAGTTTTTGGGTTCCGGGGGGAGTATGGTCGCAAGGCTG
TBW	PROK_01050	TACGAGGGGTGCAAGCGTTGTCCGGAATCATTGGGCGTAAAGGGTCCGTAGGCGTTTCAATAAGTCATTTGTGAAAGACACCGGCTCAACCGGTGGCCCGCATTCGAAACTGTTGAAATAGAGTATAGGAGAGGAAAG<br>TGGAATTCCTGGTGTAGCGGTGACATGCGTAGATATCAGGAGGAACACCAATGGCGAAGGCAGCTTTCTGGCCTATTACTGACGCTGAGGGACGAAAGCGTGGGGAGCGAACAGGATTAGATACCCTGGTAGTCCACG<br>CCGTAAACGATGAGTACTTGGTGTCGGGGGAATCGACCCCCTCGGTACCGGAGCTAACGCATTAAGTACTCCGCCTGGGAACTACGTCCGCAAGGATG
TWW	PROK_01014	TACGTAGGGTGCGAGCGTTAATCGGAATTACTGGGCGTAAAGCGTGCGCAGGCGGTTTTGTAAGCCAGATGTGAAATCCCCGGGCTTAACCTGGGAATGGCATTTGGGACTGCAAGGCTGGAGTGCGGCAGAGGAGAC<br>TGGAATTCCTGGTGTAGCAGTGAAATGCGTAGATATCAGGAGGAACACCGATGGCGAAGGCAGGTCTCTGGGCTGACACTGACGCTCATGCACGAAAGCGTGGGGAGCAAACAGGATTAGATACCCTGGTAGTCCACG<br>CCCTAAACGATGTCGACTGGTTGTTGGGGGTTTGACACTCTCAGTAACGAAGCTAACGCGTGAAGTCGACCGCCTGGGGAGTACGGCCGCAAGGTTA
TWW	PROK_02182	TACGTAGGGTGCAAGCGTTAATCGGAATTACTGGGCGTAAAGCGTGCGCAGGCGGTTTCGTAAGTCTGTCGTGAAATCCCCGGGCTTAACCTGGGAATGGCGATGGAGACTGCGAGGCTAGAGTTTGGCAGAGGGGG<br>GTAGAATTCCACGTGTAGCAGTGAAATGCGTAGAGATGTGGAGGAACACCGATGGCGAAGGCAGCCCCCTGGGTCAAAACTGACGCTCATGCACGAAAGCGTGGGGAGCAAACAGGATTAGATACCCTGGTAGTCCAC<br>GCCCTAAACGATGTCTACTAGTTGTTGGGTCTTAATTGACTTAGTAACGCAGCTAACGCGTGAAGTAGACCGCCTGGGGAGTACGGTCGCAAGATTA

TWW	PROK_01064	TACGAAGGGTGCAAGCGTTAATCGGAATTACTGGGCGTAAAGGGTGCGTAGGCGGTTAGTTAAGTCTGTTGTGAAATCCCCGGGCTCAACCTGGGAATGGCAATGGATACTGGCTAGCTAGAGTGTGTCAGAGGATGG TGGAATTTCCGGTGTAGCGGTGAAATGCGTAGAGATCGGAAGGAACATCAGTGGCGAAGGCGGCCATCTGGGACAACACTGACGCTGAAGCACGAAAGCGTGGGGAGCAAACAGGATTAGATACCCTGGTAGTCCAC GCCCTAAACGATGCGAACTGGATGTTGGTCTCAACTCGGAGATCAGTGTCGAAGCTAACGCGTTAAGTTCGCCGCCTGGGGAGTACGGTCGCAAGACTG

Supplementary Table 7. DNA sequences of the core microbes.

Water Mass	ASVs	DNA Sequence
CDW	PROK_00001	TACGAAGGGGGCTAGCGTTGCTCGGAATCACTGGGCGTAAAGGGCGCGTAGGCGGCGTTTTAAGTCGGGGGTGAAAGCCTGTGGCTCAACCACAGAATGGCCTTCGATACTGGGACGCTTGAGTATGGTAGAGGTTGGTGGAACTGCGAGTGTAGAGGTGAAATTCGTAGATATTCGCAAGAACACCGGTGGCGAAGGCGGCCAACTGGACCATTACTGACGCTGAGGCGCGAAAGCGTGGGGAGCAAACAGGATTAGATACCCTGGTAGTCCACGCCGTAAACGATGAATGCCAGCTGTTGGGGTGCTTGCACCGCAGTAGCGCAGCTAACGCTTTGAGCATTCCGCCTGGGGAGTACGGTCGCAAGATTA
CDW	PROK_00011	TACGTAGGTGGCAAGCGTTATCCGGAATTATTGGGCGTAAAGCGCGCGTAGGCGGTTTTTTAAGTCTGATGTGAAAGCCCACGGCTCAACCGTGGAGGGTCATTGGAAACTGGAAAACTTGAGTGCAGAAGAGGAAAGTGGAATTCCATGTGTAGCGGTGAAATGCGCAGAGATATGGAGGAACACCAGTGGCGAAGGCGACTTTCTGGTCTGTAACTGACGCTGATGTGCGAAAGCGTGGGGATCAAACAGGATTAGATACCCTGGTAGTCCACGCCGTAAACGATGAGTGCTAAGTGTTAGGGGGTTTCCGCCCCTTAGTGCTGCAGCTAACGCATTAAGCACTCCGCCTGGGGAGTACGACCGCAAGGTTG
CDW	PROK_00046	TACGGAGGGTGCAAGCGTTAATCGGAATTACTGGGCGTAAAGCGCACGCAGGCGGTTTGTTAAGTCAGATGTGAAATCCCCGGGCTCAACCTGGGAACTGCATCTGATACTGGCAAGCTTGAGTCTCGTAGAGGGGGGTAGAATTCCAGGTGTAGCGGTGAAATGCGTAGAGATCTGGAGGAATACCGGTGGCGAAGGCGGCCCCCTGGACGAAGACTGACGCTCAGGTGCGAAAGCGTGGGGAGCAAACAGGATTAGATACCCTGGTAGTCCACGCCGTAAACGATGTCGACTTGGAGGTTGTGCCCTTGAGGCGTGGCTTCCGGAGCTAACGCGTTAAGTCGACCGCCTGGGGAGTACGGCCGCAAGGTTA
CDW	PROK_00094	TACGTAGGGTGCAAGCGTTAATCGGAATTACTGGGCGTAAAGCGTGCGCAGGCGGTTTTGTAAGTCTGTCGTGAAAGCCCCGGGCTCAACCTGGGAATTGCGATGGAGACTGCAAGGCTTGAATCTGGCAGAGGGGGGTAGAATTCCACGTGTAGCAGTGAAATGCGTAGAGATGTGGAGGAACACCGATGGCGAAGGCAGCCCCCTGGGTCAAGATTGACGCTCATGCACGAAAGCGTGGGGAGCAAACAGGATTAGATACCCTGGTAGTCCACGCCCTAAACGATGTCTACTAGTTGTCGGGTTTTAATTAACTTGGTAACGCAGCTAACGCGTGAAGTAGACCGCCTGGGGAGTACGGTCGCAAGATTA
CDW	PROK_00156	TACGTAGGGTGCAAGCGTTAATCGGAATTACTGGGCGTAAAGCGTGCGCAGGCGGTTTTGTAAGTCTGACGTGAAATCCCCGGGCTCAACCTGGGAATTGCGTTGGAGACTGCAAGGCTGGAGTCTGGCAGAGGGGGGTAGAATTCCACGTGTAGCAGTGAAATGCGTAGAGATGTGGAGGAACACCGATGGCGAAGGCAGCCCCCTGGGTCAAGACTGACGCTCATGCACGAAAGCGTGGGGAGCAAACAGGATTAGATACCCTGGTAGTCCACGCCCTAAACGATGTCTACTAGTTGTCGGGTCTTAATTGACTTGGTAACGCAGCTAACGCGTGAAGTAGACCGCCTGGGGAGTACGGTCGCAAGATTA
CDW	PROK_00173	TACGTAGGGTGCAAGCGTTAATCGGAATTACTGGGCGTAAAGCGTGCGCAGGCGGTTTTGTAAGTCTGTCGTGAAATCCCCGGGCTCAACCTGGGAATTGCGATGGAGACTGCAAGGCTGGAGTCTGGCAGAGGGGGGTAGAATTCCACGTGTAGCAGTGAAATGCGTAGAGATGTGGAGGAACACCGATGGCGAAGGCAGCCCCCTGGGTCAAGACTGACGCTCATGCACGAAAGCGTGGGGAGCAAACAGGATTAGATACCCTGGTAGTCCACGCCCTAAACGATGTCTACTAGTTGTCGGGTCTTAATTGACTTGGTAACGCAGCTAACGCGTGAAGTAGACCGCCTGGGGAGTACGGTCGCAAGATTA
TWW	PROK_00001	TACGAAGGGGGCTAGCGTTGCTCGGAATCACTGGGCGTAAAGGGCGCGTAGGCGGCGTTTTAAGTCGGGGGTGAAAGCCTGTGGCTCAACCACAGAATGGCCTTCGATACTGGGACGCTTGAGTATGGTAGAGGTTGGTGGAACTGCGAGTGTAGAGGTGAAATTCGTAGATATTCGCAAGAACACCGGTGGCGAAGGCGGCCAACTGGACCATTACTGACGCTGAGGCGCGAAAGCGTGGGGAGCAAACAGGATTAGATACCCTGGTAGTCCACGCCGTAAACGATGAATGCCAGCTGTTGGGGTGCTTGCACCGCAGTAGCGCAGCTAACGCTTTGAGCATTCCGCCTGGGGAGTACGGTCGCAAGATTA
TWW	PROK_00046	TACGGAGGGTGCAAGCGTTAATCGGAATTACTGGGCGTAAAGCGCACGCAGGCGGTTTGTTAAGTCAGATGTGAAATCCCCGGGCTCAACCTGGGAACTGCATCTGATACTGGCAAGCTTGAGTCTCGTAGAGGGGGGTAGAATTCCAGGTGTAGCGGTGAAATGCGTAGAGATCTGGAGGAATACCGGTGGCGAAGGCGGCCCCCTGGACGAAGACTGACGCTCAGGTGCGAAAGCGTGGGGAGCAAACAGGATTAGATACCCTGGTAGTCCACGCCGTAAACGATGTCGACTTGGAGGTTGTGCCCTTGAGGCGTGGCTTCCGGAGCTAACGCGTTAAGTCGACCGCCTGGGGAGTACGGCCGCAAGGTTA
TWW	PROK_00094	TACGTAGGGTGCAAGCGTTAATCGGAATTACTGGGCGTAAAGCGTGCGCAGGCGGTTTTGTAAGTCTGTCGTGAAAGCCCCGGGCTCAACCTGGGAATTGCGATGGAGACTGCAAGGCTTGAATCTGGCAGAGGGGGGTAGAATTCCACGTGTAGCAGTGAAATGCGTAGAGATGTGGAGGAACACCGATGGCGAAGGCAGCCCCCTGGGTCAAGATTGACGCTCATGCACGAAAGCGTGGGGAGCAAACAGGATTAGATACCCTGGTAGTCCACGCCCTAAACGATGTCTACTAGTTGTCGGGTTTTAATTAACTTGGTAACGCAGCTAACGCGTGAAGTAGACCGCCTGGGGAGTACGGTCGCAAGATTA
TWW	PROK_00156	TACGTAGGGTGCAAGCGTTAATCGGAATTACTGGGCGTAAAGCGTGCGCAGGCGGTTTTGTAAGTCTGACGTGAAATCCCCGGGCTCAACCTGGGAATTGCGTTGGAGACTGCAAGGCTGGAGTCTGGCAGAGGGGGGTAGAATTCCACGTGTAGCAGTGAAATGCGTAGAGATGTGGAGGAACACCGATGGCGAAGGCAGCCCCCTGGGTCAAGACTGACGCTCATGCACGAAAGCGTGGGGAGCAAACAGGATTAGATACCCTGGTAGTCCACGCCCTAAACGATGTCTACTAGTTGTCGGGTCTTAATTGACTTGGTAACGCAGCTAACGCGTGAAGTAGACCGCCTGGGGAGTACGGTCGCAAGATTA
GMW	PROK_00001	TACGAAGGGGGCTAGCGTTGCTCGGAATCACTGGGCGTAAAGGGCGCGTAGGCGGCGTTTTAAGTCGGGGGTGAAAGCCTGTGGCTCAACCACAGAATGGCCTTCGATACTGGGACGCTTGAGTATGGTAGAGGTTGGTGGAACTGCGAGTGTAGAGGTGAAATTCGTAGATATTCGCAAGAACACCGGTGGCGAAGGCGGCCAACTGGACCATTACTGACGCTGAGGCGCGAAAGCGTGGGGAGCAAACAGGATTAGATACCCTGGTAGTCCACGCCGTAAACGATGAATGCCAGCTGTTGGGGTGCTTGCACCGCAGTAGCGCAGCTAACGCTTTGAGCATTCCGCCTGGGGAGTACGGTCGCAAGATTA
GMW	PROK_00003	TACGTAGGGTGCAAGCGTTAATCGGAATTACTGGGCGTAAAGCGTGCGCAGGCGGTTTTGTAAGTCTGTCGTGAAATCCCCGGGCTCAACCTGGGAATTGCGATGGAGACTGCAAGGCTGGAGTCTGGCAGAGGGGGGTAGAATTCCACGTGTAGCAGTGAAATGCGTAGAGATGTGGAGGAACACCGATGGCGAAGGCAGCCCCCTGGGTCAAGACTGACGCTCATGCACGAAAGCGTGGGGAGCAAACAGGATTAGATACCCTGGTAGTCCACGCCCTAAACGATGTCTACTAGTTGTCGGGTCTTAATTGACTTGGTAACGCAGCTAACGCGTGAAGTAGACCGCCTGGGGAGTACGGTCGCAAGATTA
GMW	PROK_00020	TACGGAGGGTCCAAGCGTTATCCGGAATCATTGGGTTTAAAGGGTCCGTAGGCGGGCTATTAAGTCAGAGGTGAAAGTTTGCAGCTCAACTGTAAAATTGCCTTTGAAACTGGTAGTCTTGAATTATTATGAAGTGGTTAGAATAAGTAGTGTAGCGGTGAAATGCATAGATATTACTTAGAATACCAATTGCGAAGGCAGATCACTAATAATATATTGACGCTGAGGGACGAAAGCGTGGGGAGCGAACAGGATTAGATACCCTGGTAGTCCACGCCGTAAACGATGGATACTAGCTGTTCGGATTTATCTGAGTGGCTAAGCGAAAGTGATAAGTATCCCACCTGGGGAGTACGTTCGCAAGAATG

GMW	PROK_00024	TACGGAGGGTCCAAGCGTTATCCGGATTTATTGGGTTTAAAGGGTCCGTAGGCGGGTCATTAAGTCAGTGGTGAAATCCTACAGCTCAACTGTAGAACTGCCATTGAAACTGGTGACCTTGAATATAATTGAAGTAGGCGGAATGTGTCATGTAGCGGTGAAATGCTTAGATATGCCACAGAACACCGATTGCGAAGGCAGCTTACTAAGTTATTATTGACGCTGAGGGACGAAAGCGTGGGTAGCGAACAGGATTAGATACCCTGGTAGTCCACGCCGTAAACGATGTATACTCGTTGCTAGCGATACACAGTTAGTGACCAAGCGAAAGTATTAAGTATACCACCTGGGGAGTACGATCGCAAGATTG
GMW	PROK_00032	TACGGAGGGTCCGAGCGTTATCCGGAATCATTGGGTTTAAAGGGTCCGCAGGCGGTCTATTAAGTCAGAGGTGAAATCCCATAGCTCAACTATGGAACTGCCTTTGATACTGGTTGACTTGAGTCATATGGAAGTGGATAGAATGTGTAGTGTAGCGGTGAAATGCATAGATATTACACAGAATACCGATTGCGAAGGCAGTCCACTACGTATGTACTGACGCTGAGGGACGAAAGCGTGGGGAGCGAACAGGATTAGATACCCTGGTAGTCCACGCCGTAAACGATGGATACTAGTTGTTGGGATTTATCTCAGTGACTAAGCGAAAGTGATAAGTATCCCACCTGGGGAGTACGGTCGCAAGACTG
GMW	PROK_00043	TACGGAGGGTCCAAGCGTTATCCGGATTTATTGGGTTTAAAGGGTCCGTAGGCGGGAGAATAAGTCAGTGGTGAAAGCCTACAGCTCAACTGTAGAACTGCCATTGAAACTGTTTTTCTTGAATTTAGATGAAGTGGGCGGAATATATCATGTAGCGGTGAAATGCATAGATATGATATAGAACACCGATTGCGAAGGCAGCTCACTAAACTAATATTGACGCTGAGGGACGAAAGTGTGGGGAGCGAACAGGATTAGATACCCTGGTAGTCCACACCGTAAACGATGATTACTCGATGCTAGCGATATACTGTTAGTGTCTAAGCGAAAGCGATAAGTAATCCACCTGGGGAGTACGGTCGCAAGATTG
AASW	PROK_00001	TACGAAGGGGGCTAGCGTTGCTCGGAATCACTGGGCGTAAAGGGCGCGTAGGCGGCGTTTTAAGTCGGGGGTGAAAGCCTGTGGCTCAACCACAGAATGGCCTTCGATACTGGGACGCTTGAGTATGGTAGAGGTTGGTGGAACTGCGAGTGTAGAGGTGAAATTCGTAGATATTCGCAAGAACACCGGTGGCGAAGGCGGCCAACTGGACCATTACTGACGCTGAGGCGCGAAAGCGTGGGGAGCAAACAGGATTAGATACCCTGGTAGTCCACGCCGTAAACGATGAATGCCAGCTGTTGGGGTGCTTGCACCGCAGTAGCGCAGCTAACGCTTTGAGCATTCCGCCTGGGGAGTACGGTCGCAAGATTA
AASW	PROK_00003	TACGTAGGGTGCAAGCGTTAATCGGAATTACTGGGCGTAAAGCGTGCGCAGGCGGTTTTGTAAGTCTGTCGTGAAATCCCCGGGCTCAACCTGGGAATTGCGATGGAGACTGCAAGGCTGGAGTCTGGCAGAGGGGGGTAGAATTCCACGTGTAGCAGTGAAATGCGTAGAGATGTGGAGGAACACCGATGGCGAAGGCAGCCCCCTGGGTCAAGACTGACGCTCATGCACGAAAGCGTGGGGAGCAAACAGGATTAGATACCCTGGTAGTCCACGCCCTAAACGATGTCTACTAGTTGTCGGGTCTTAATTGACTTGGTAACGCAGCTAACGCGTGAAGTAGACCGCCTGGGGAGTACGGTCGCAAGATTA
AASW	PROK_00015	TACGGAGGGTGCAAGCGTTAATCGGAATTACTGGGCGTAAAGCGCGCGTAGGTGGTTTGTTAAGTGGAATGTGAAAGCCCTGGGCTCAACCTAGGAATTGCATCCCAAACTGGCAGACTAGAGTACAAGAGAGGGGTGTGGAATTTCCTGTGTAGCGGTGAAATGCGTAGATATAGGAAGGAACATCAGTGGCGAAGGCGACGCCCTGGCTTGATACTGACACTGAGGTGCGAAAGCGTGGGGAGCAAACAGGATTAGATACCCTGGTAGTCCACGCCGTAAACGATGTCTACTAGCCGTTGGGGGACTTGATCCCTTAGTGGCGAAGCTAACGCGATAAGTAGACCGCCTGGGGAGTACGGTCGCAAGATTA
TBW	PROK_00001	TACGAAGGGGGCTAGCGTTGCTCGGAATCACTGGGCGTAAAGGGCGCGTAGGCGGCGTTTTAAGTCGGGGGTGAAAGCCTGTGGCTCAACCACAGAATGGCCTTCGATACTGGGACGCTTGAGTATGGTAGAGGTTGGTGGAACTGCGAGTGTAGAGGTGAAATTCGTAGATATTCGCAAGAACACCGGTGGCGAAGGCGGCCAACTGGACCATTACTGACGCTGAGGCGCGAAAGCGTGGGGAGCAAACAGGATTAGATACCCTGGTAGTCCACGCCGTAAACGATGAATGCCAGCTGTTGGGGTGCTTGCACCGCAGTAGCGCAGCTAACGCTTTGAGCATTCCGCCTGGGGAGTACGGTCGCAAGATTA
TBW	PROK_00003	TACGTAGGGTGCAAGCGTTAATCGGAATTACTGGGCGTAAAGCGTGCGCAGGCGGTTTTGTAAGTCTGTCGTGAAATCCCCGGGCTCAACCTGGGAATTGCGATGGAGACTGCAAGGCTGGAGTCTGGCAGAGGGGGGTAGAATTCCACGTGTAGCAGTGAAATGCGTAGAGATGTGGAGGAACACCGATGGCGAAGGCAGCCCCCTGGGTCAAGACTGACGCTCATGCACGAAAGCGTGGGGAGCAAACAGGATTAGATACCCTGGTAGTCCACGCCCTAAACGATGTCTACTAGTTGTCGGGTCTTAATTGACTTGGTAACGCAGCTAACGCGTGAAGTAGACCGCCTGGGGAGTACGGTCGCAAGATTA
TBW	PROK_00015	TACGGAGGGTGCAAGCGTTAATCGGAATTACTGGGCGTAAAGCGCGCGTAGGTGGTTTGTTAAGTGGAATGTGAAAGCCCTGGGCTCAACCTAGGAATTGCATCCCAAACTGGCAGACTAGAGTACAAGAGAGGGGTGTGGAATTTCCTGTGTAGCGGTGAAATGCGTAGATATAGGAAGGAACATCAGTGGCGAAGGCGACGCCCTGGCTTGATACTGACACTGAGGTGCGAAAGCGTGGGGAGCAAACAGGATTAGATACCCTGGTAGTCCACGCCGTAAACGATGTCTACTAGCCGTTGGGGGACTTGATCCCTTAGTGGCGAAGCTAACGCGATAAGTAGACCGCCTGGGGAGTACGGTCGCAAGATTA
TBW	PROK_00020	TACGGAGGGTCCAAGCGTTATCCGGAATCATTGGGTTTAAAGGGTCCGTAGGCGGGCTATTAAGTCAGAGGTGAAAGTTTGCAGCTCAACTGTAAAATTGCCTTTGAAACTGGTAGTCTTGAATTATTATGAAGTGGTTAGAATAAGTAGTGTAGCGGTGAAATGCATAGATATTACTTAGAATACCAATTGCGAAGGCAGATCACTAATAATATATTGACGCTGAGGGACGAAAGCGTGGGGAGCGAACAGGATTAGATACCCTGGTAGTCCACGCCGTAAACGATGGATACTAGCTGTTCGGATTTATCTGAGTGGCTAAGCGAAAGTGATAAGTATCCCACCTGGGGAGTACGTTCGCAAGAATG
TBW	PROK_00024	TACGGAGGGTCCAAGCGTTATCCGGATTTATTGGGTTTAAAGGGTCCGTAGGCGGGTCATTAAGTCAGTGGTGAAATCCTACAGCTCAACTGTAGAACTGCCATTGAAACTGGTGACCTTGAATATAATTGAAGTAGGCGGAATGTGTCATGTAGCGGTGAAATGCTTAGATATGCCACAGAACACCGATTGCGAAGGCAGCTTACTAAGTTATTATTGACGCTGAGGGACGAAAGCGTGGGTAGCGAACAGGATTAGATACCCTGGTAGTCCACGCCGTAAACGATGTATACTCGTTGCTAGCGATACACAGTTAGTGACCAAGCGAAAGTATTAAGTATACCACCTGGGGAGTACGATCGCAAGATTG
TBW	PROK_00027	TACGGAGGGTGCAAGCGTTAATCGGAATTACTGGGCGTAAAGCGCGCGTAGGTGGTTTGTTAAGTGGAATGTGAAAGCCCTGGGCTCAACCTAGGAATTGCATCCCAAACTGGCAAACTAGAGTACAAGAGAGGGGTGTGGAATTTCCTGTGTAGCGGTGAAATGCGTAGATATAGGAAGGAACATCAGTGGCGAAGGCGACGCCCTGGCTTGATACTGACACTGAGGTGCGAAAGCGTGGGGAGCAAACAGGATTAGATACCCTGGTAGTCCACGCCGTAAACGATGTCTACTAGCCGTTGGGGGACTTGATCCCTTAGTGGCGAAGCTAACGCGATAAGTAGACCGCCTGGGGAGTACGGTCGCAAGATTA
TBW	PROK_00032	TACGGAGGGTCCGAGCGTTATCCGGAATCATTGGGTTTAAAGGGTCCGCAGGCGGTCTATTAAGTCAGAGGTGAAATCCCATAGCTCAACTATGGAACTGCCTTTGATACTGGTTGACTTGAGTCATATGGAAGTGGATAGAATGTGTAGTGTAGCGGTGAAATGCATAGATATTACACAGAATACCGATTGCGAAGGCAGTCCACTACGTATGTACTGACGCTGAGGGACGAAAGCGTGGGGAGCGAACAGGATTAGATACCCTGGTAGTCCACGCCGTAAACGATGGATACTAGTTGTTGGGATTTATCTCAGTGACTAAGCGAAAGTGATAAGTATCCCACCTGGGGAGTACGGTCGCAAGACTG
TBW	PROK_00036	TACGGAGGGTGCAAGCGTTAATCGGAATTACTGGGCGTAAAGCGTGCGTAGGCGGTTTATTAAGTCAGATGTGAAAGCCCCGGGCTTAACCTGGGAACTGCATTTGAAACTGGTCAACTAGAGTATGGTAGAGGAAAGTGGAATTTCTGGTGTAGCGGTGAAATGCGTAGATATCAGAAGGAACATCAATGGCGAAGGCAACTTTCTGGACCAATACTGACGCTGAGGTACGAAAGCGTGGGTAGCAAACAGGATTAGATACCCTGGTAGTCCACGCCGTAAACGATGATAACTAGCCGTTGGGGGGATTTACCCCTTAGTGGCGAAGCTAACGCGTTAAGTTATCCGCCTGGGGAGTACGGCCGCAAGGTTA
TBW	PROK_00045	TACGGAGGATCCAAGCGTTATCCGGAATCATTGGGTTTAAAGGGTCCGTAGGCGGTCTTTTAAGTCAGAGGTGAAATCCTACAGCTCAACTGTAGAATTGCCTTTGATACTGAAAGACTTGAGTTATTGTGAAGTAGTTAGAATGTGTGGTGTAGCGGTGAAATGCATAGAGATCACACAGAATACCGATTGCGAAGGCAGATTACTAACAATATACTGACGCTGAGGGACGAAAGCGTGGGTAGCGAACAGGATTAGATACCCTGGTAGTCCACGCCGTAAACGATGGATACTAGCTGTTCGACCAACATTGGTTGGTTGAGTGGCTAAGCGAAAGTGATAAGTATCCCACCTGGGGAGTACGGTCGCAAGACTG

TBW	PROK_00059	TACGAAGGGACCTAGCGTAGTTCGGAATTACTGGGCTTAAAGAGTTCGTAGGTGGTTGAAAAAGTTGGTGGTGAAATCCCAGAGCTTAACTCTGGAACTGCCATCAAAACTTTTCAGCTAGAGTATGATAGAGGAAAGCAGAATTTCTAGTGTAGAGGTGAAATTCGTAGATATTAGAAAGAATACCAATTGCGAAGGCAGCTTTCTGGATCATTACTGACACTGAGGAACGAAAGCATGGGTAGCGAAGAGGATTAGATACCCTCGTAGTCCATGCCGTAAACGATGTGTGTTAGACGTTGGAAATTTATTTTCAGTGTCGCAGGGAAACCGATAAACACACCGCCTGGGGAGTACGACCGCAAGGTTA
TBW	PROK_00094	TACGTAGGGTGCAAGCGTTAATCGGAATTACTGGGCGTAAAGCGTGCGCAGGCGGTTTTGTAAGTCTGTCGTGAAAGCCCCGGGCTCAACCTGGGAATTGCGATGGAGACTGCAAGGCTTGAATCTGGCAGAGGGGGGTAGAATTCCACGTGTAGCAGTGAAATGCGTAGAGATGTGGAGGAACACCGATGGCGAAGGCAGCCCCCTGGGTCAAGATTGACGCTCATGCACGAAAGCGTGGGGAGCAAACAGGATTAGATACCCTGGTAGTCCACGCCCTAAACGATGTCTACTAGTTGTCGGGTTTTAATTAACTTGGTAACGCAGCTAACGCGTGAAGTAGACCGCCTGGGGAGTACGGTCGCAAGATTA

Supplementary Table 8. Different metrics of co-occurrence networks calculated for each of the water masses considered in this study.

	Nodes	Edges	Average distance	Modularity index	Average degree	Average clustering	Average density
**General**	384	776	2.1798	0.5855	4.0417	0.4535	0.0105
**AASW**	137	722	2.1404	0.3465	10.5401	0.5767	0.0775
**GMW**	112	849	2.0343	0.2233	15.1607	0.6081	0.1366
**TBW**	264	748	2.1587	0.4298	5.6667	0.4704	0.0215
**TWW**	176	138	1.3067	0.9579	1.5682	0.4912	0.0089
**CDW**	42	83	2.5034	0.5784	3.9524	0.4071	0.0964

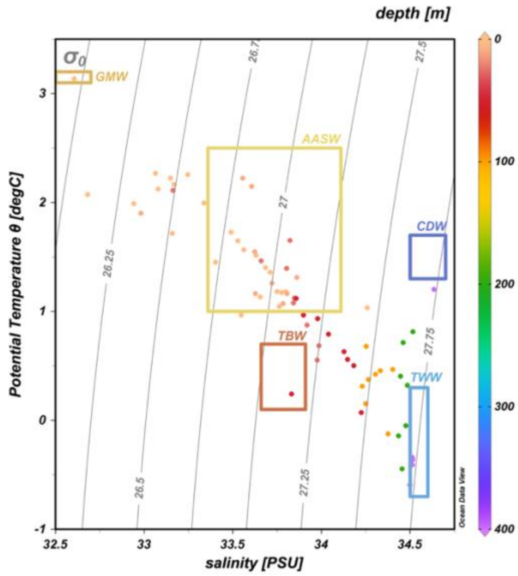

Supplementary Figure 23. Temperature-Salinity (*T-S*) diagram with water mass cores included. Details about water masses are included in Supplementary Table 2. Triangles applied: GMW-AASW-TBW; AASW-TBW-TWW; TBW-TWW-CDW.

Supplementary Table 9. Formulas used for calculating alpha diversity. For the Shannon index: *pi* is the relative abundance of species *i*. For the Simpson index: *ni* is the number of reads belonging to species *i* and *N* is the total number of reads. For the Pielou index: *pi* is the proportion of the total sample represented by species *i* and *S* is the number of species in a sample.

**Shannon index (H')**	**Simpson index (D')**	**Pielou index (J')**
$H' = -\Sigma(p_i \ln(p_i))$	$D' = \Sigma n_i(n_i-1)/N(N-1)$	$J' = -\Sigma p_i \ln(p_i)/\ln(S)$
